# Discovery of novel 2,3,4,5-tetrahydrospiro[benzo[*c*]azepine-1,1’-cyclohexan]-5-ol derivatives as PARP-1 inhibitors

**DOI:** 10.1186/s13065-023-01060-8

**Published:** 2023-10-27

**Authors:** Ling Yu, Jian-hui Li, Ju Zhu, You-de Wang, Zhi-wei Yan, Li-ying Zhang, Shuai Li

**Affiliations:** 1 Department of Pharmacy, Anorectal Hospital of Chengde Medical University, Chengde, 067000 P. R. China; 2 Department of Preventive Medicine, Chengde Medical University, Chengde, 067000 P. R. China; 3 School of Pharmacy, China Medical University, 77 Puhe Road, North New Area, Shenyang, 110122 China; 4 Key Laboratory of Traditional Chinese Medicine Research and Development of Hebei Province, Hebei Key Laboratory of Nerve Injury and Repair, Institute of Traditional Chinese Medicine, Chengde Medical University, Anyuan Road, Chengde, 067000 P. R. China

**Keywords:** PARP-1 inhibitor, Antitumor, Apoptosis, Drug discovery

## Abstract

As an essential marker of cancer treatment, PARP-1 inhibitors could effectively kill tumor cells through a mechanism known as synthetic lethality and are used to treat a variety of cancers. In order to explore novel PARP-1 inhibitors, a series of 22 novel erythrina derivatives were reported and preliminarily explored their mechanism of action. The antitumor activities against four human cancer cell lines including A549, OVCAR-3, HCT-116, and MCF-7 were evaluated, and the preliminary SARs were summarized. Among them, compound **11b** exhibited better anti-proliferative effects against A549 cells (IC_50_ = 1.95 µM). The SI results showed that compound **11b** had low toxicity. Moreover, compound **11b** displayed excellent PARP-1 inhibitory activities with IC_50_ values of 19.24 nM. In addition, molecular docking studies provided the rational binding modes of compound **11b** in complexes with PARP-1. The flow cytometry assays revealed that compound **11b** could induce apoptosis of A549 cells (P < 0.001). Simultaneously, compound **11b** could effectively reduce the formation of PAR (P < 0.001). The ADMET prediction results indicated compound **11b** had similar properties to rucaparib. Collectively, compound **11b** has potential research value for further investigation.

**Supplementary Information:**

The online version contains supplementary material available at 10.1186/s13065-023-01060-8.

## Introduction

Cancer is a large group of diseases characterized by the uncontrollable growth of abnormal cells [1]. To date, the family of poly (ADP-ribose) polymerase (PARP) proteins has 18 members that share structural and functional similarities while constituting a diverse and remarkable group of proteins [2]. Among this family, PARP-1 is the most abundant and specific isoform, exerting more than 90% of the PARP enzyme activity [3, 4]. Therefore, PARP-1 is considered an ideal target for cancer chemotherapy.

**Fig. 1:**
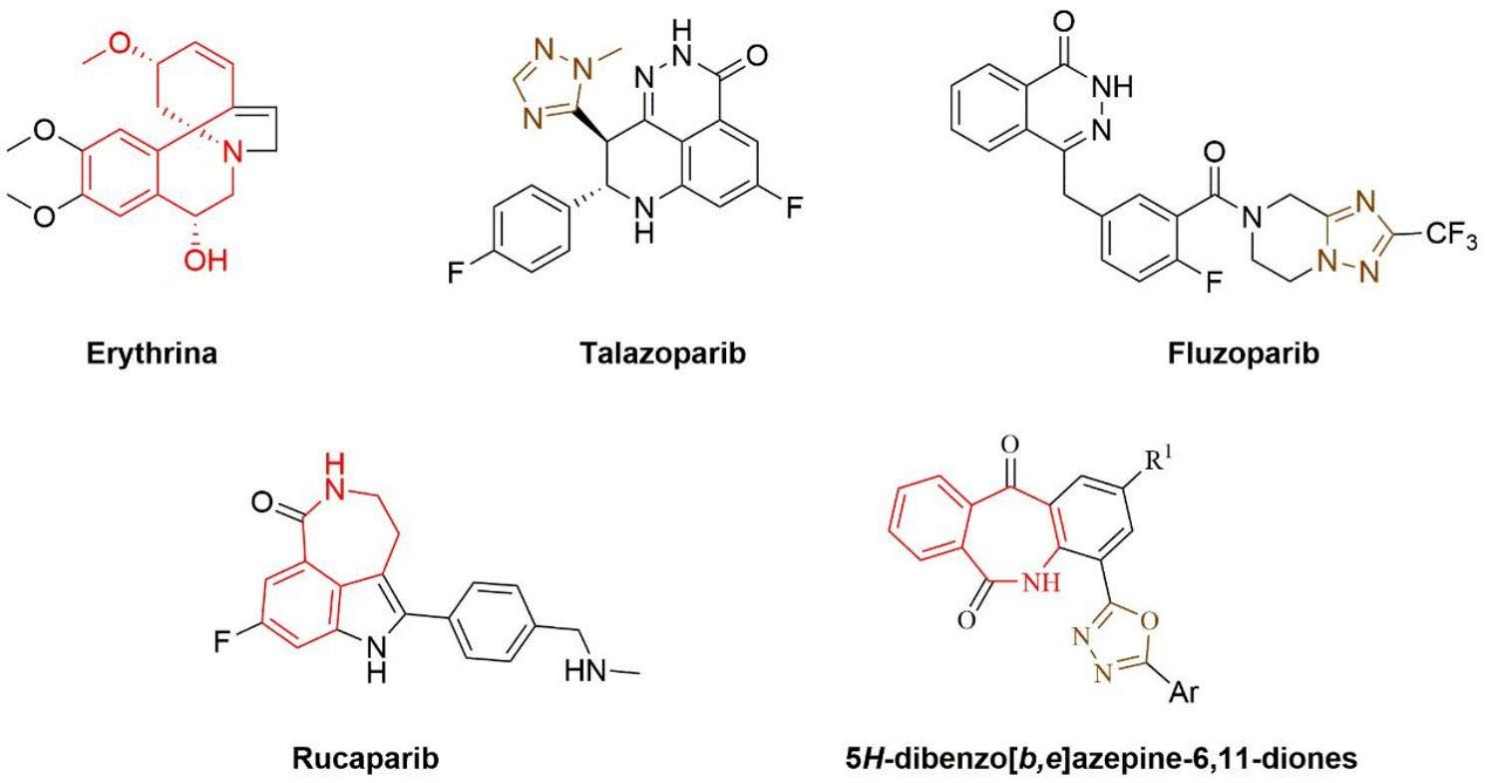
The structures of erythrina, talazoparib, fluzoparib, rucaparib and 5* H*-dibenzo[*b,e*]azepine-6,11-dione derivatives

Natural products are now explored as new alternatives for cancer treatment [5]. As a member of the Fabaceae family, erythrina has been reported to have potential antitumor activities (Fig. 1) [6–10]. In addition, we are very interested in the unique structure of the benzo-spiral ring. Rucaparib is a PARP-1 inhibitor approved by the Food and Drug Administration (FDA) for cancer treatment in 2016 [11, 12]. 5* H*-dibenzo[*b,e*]azepine-6,11-dione derivatives are the novel PARP-1 inhibitors we reported previously (Fig. 1) [13]. Furthermore, we found that they have structural similarities (red parts in Fig. 1). Therefore, we attempted to explore novel antitumor drugs targeting PARP-1 using erythrina and 5* H*-dibenzo[*b,e*]azepine-6,11-dione derivatives as the lead compounds.

As a key enzyme involved in DeoxyriboNucleic Acid (DNA) damage repair and recombination, PARP-1 shows excellent potential in developing antitumor drugs [14–16]. In addition, several novel PARP-1 inhibitors have been approved by the FDA in recent years [17, 18]. Among them, talazoparib and fluzoparib were approved for cancer treatment in 2018 and 2020, respectively (Fig. 1) [19–22]. We found that the structure of talazoparib and fluzoparib both contain a triazole group (brown parts in Fig. 1). Triazole groups are not merely passive linkers; they readily bind to biological targets through hydrogen bonding and dipole interactions. Simultaneously, the triazole group is a privileged building block in the discovery of novel antitumor agents, and some of its derivatives have already been applied in clinics to treat cancer [23–25]. So, a triazole group was designed in the target structure to improve the compounds’ activity further.

**Fig. 2:**
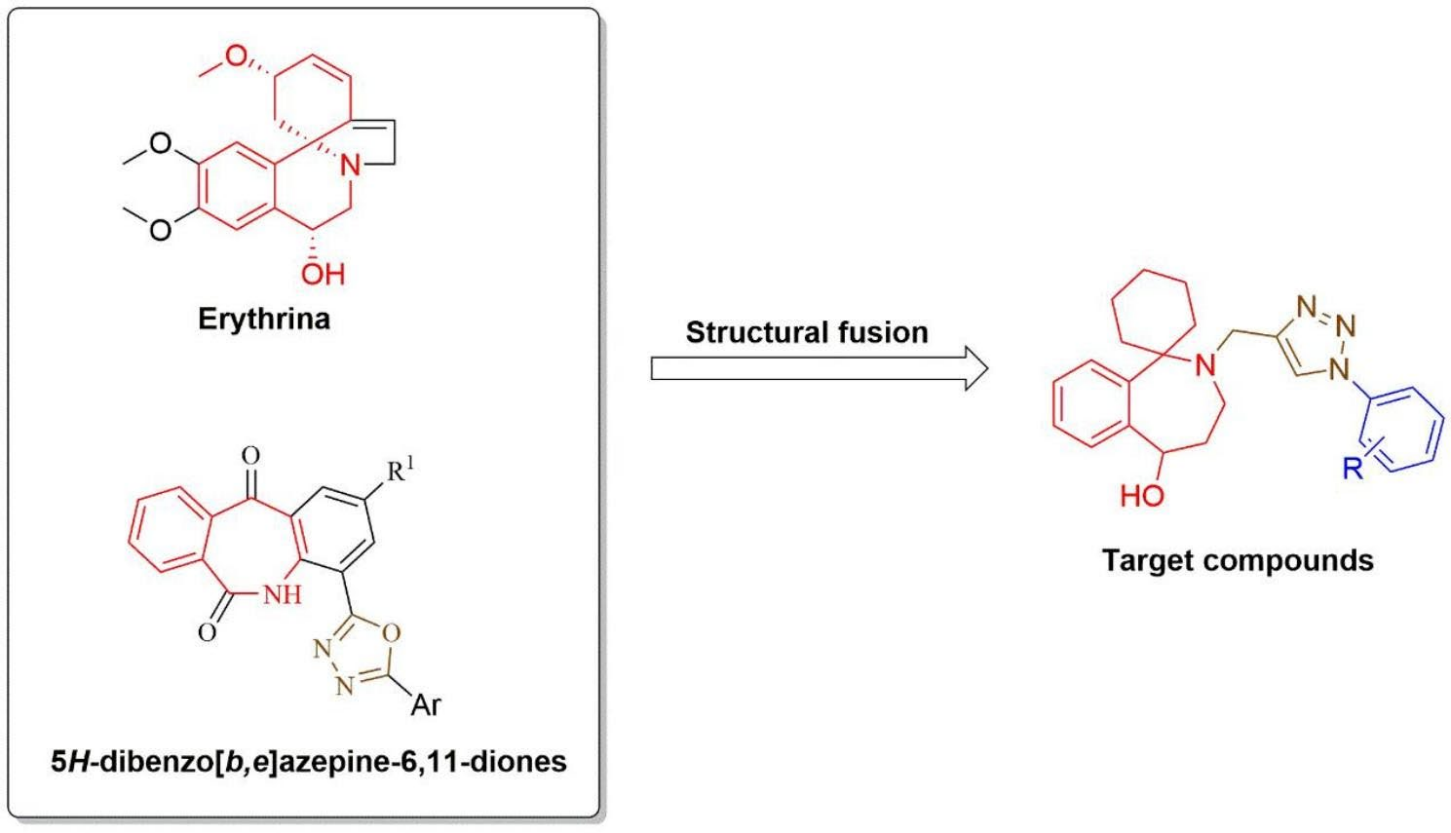
Design strategy of the target compounds

Herein, we designed and synthesized a series of erythrina derivatives as potential PARP-1 inhibitors (Fig. 2**)**. First, we performed 3-(4,5-dimethyl-2-thiazolyl)-2,5-diphenyltetrazolium bromide (MTT) assay of target compounds, and found that compound **11b** had better inhibitory activity on A549 (IC_50_ = 1.95 µM). Compound **11b** was then investigated for selectivity index (HPAEpiC: SI = 15.38) and PARP-1/2 inhibition (PARP-1: IC_50_ = 19.24 nM; PARP-2: IC_50_ = 32.58 nM). Flow cytometry and western blot indicated that compound **11b** could effectively induce the apoptosis of A549 cells, and reduce the biosynthesis of poly (ADP-ribose) (PAR). Next, the molecular docking and molecular dynamics results further elucidated the binding mode and stability of compound **11b** to PARP-1. The prediction results of ADMET indicated that compound **11b** and rucaparib had similar pharmacokinetic characteristics. All these findings indicated that compound **11b** could provide new scaffolds for developing novel PARP-1 inhibitors applicable to cancer therapy.

## Results and discussion

### Chemistry

**Scheme 1:**
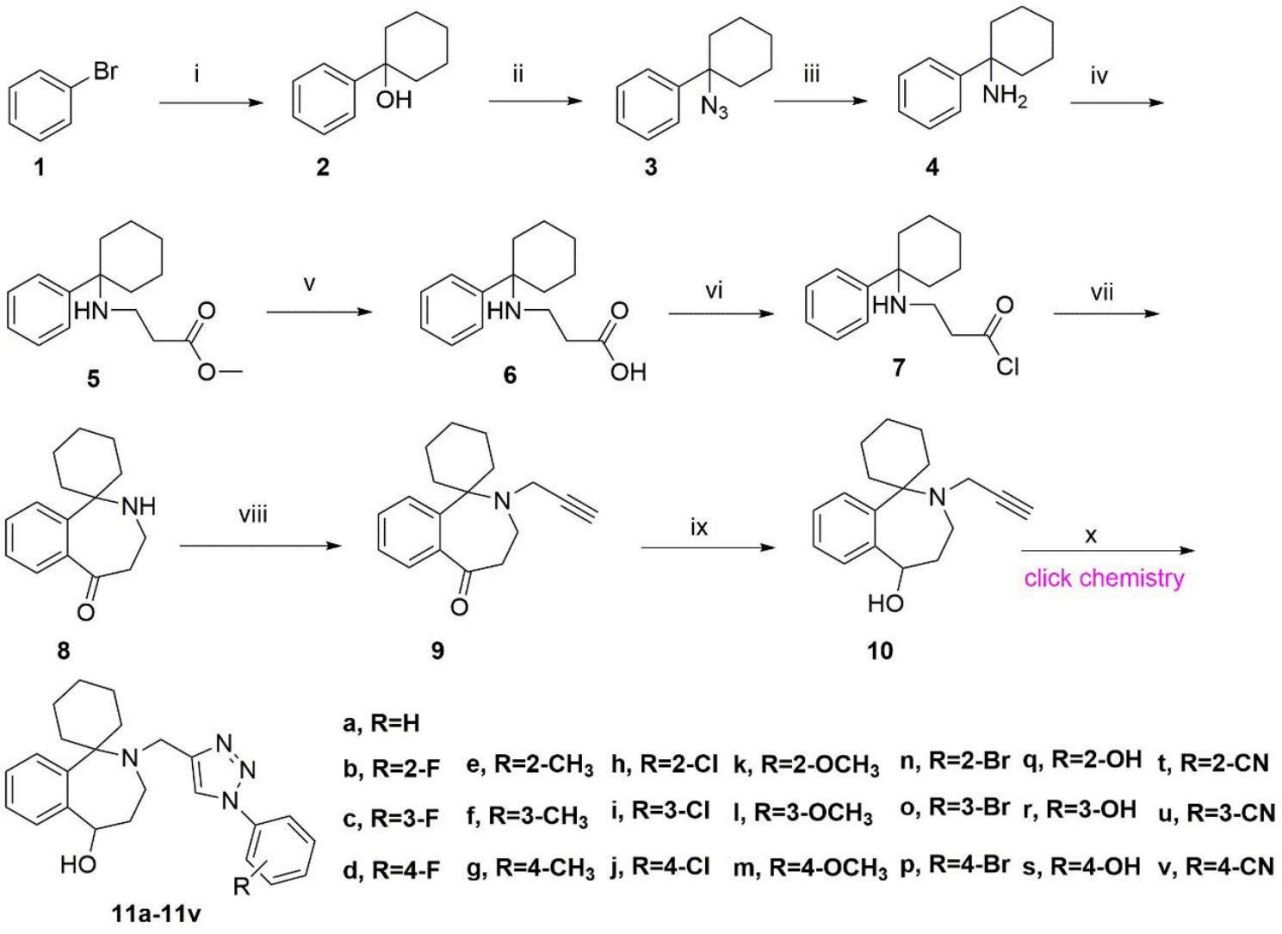
Reactions and conditions: (i) Mg, cyclohexanone, ethoxyethane, 35℃, 3 h; (ii) NaN_3_, 20℃, 12 h; (iii) LiAlH_4_, 40℃, 12 h; (iv) Methyl-acrylate, no solvent required, 40℃, 12 h; (v) NaOH, water, 50℃, 2 h; (vi) SOCl_2_, 35℃, 2 h; (vii) AlCl_3_, 25℃, 24 h; (viii) 3-bromoprop-1-yne, 70℃, 12 h; (ix) LiAlH_4_, 60℃, 12 h; (x) Corresponding azidebenzene, 35℃, 9 min, 71.91-84.00%

The synthetic routes adopted for preparing target compounds (**11a-11v**) were depicted in Scheme 1.

Compound **9** was obtained according to the method reported by our previous study [26]. Next, compound **10** was obtained by the reduction reaction with compound **9**. Finally, target compounds were obtained by the click chemistry of compound **10** with the corresponding azidebenzene. The triazole ring is a crucial five-membered heterocycle structure for designing novel bioactive molecules. This electron-rich heterocycle easily binds to various types of enzymes and receptors. Therefore, triazole compounds have a broad spectrum of biological activities [27, 28]. Meanwhile, we used the microwave reaction generator to accelerate the reaction speed and increase the reaction yield in the reaction process. ^1^ H NMR, ^13^ C NMR and HRMS spectroscopy confirmed the structures of target compounds.

### Biological evaluation

#### MTT assay and structure-activity relationship (SAR) analysis

The anti-proliferative effects on A549, OVCAR-3, HCT-116 and MCF-7 cells were evaluated by MTT assay, and rucaparib as a positive control. (Table 1)

**Table 1:** Anti-proliferative effects of target compounds against tumor cell lines

Compounds	IC_50_^[a]^(µM)						
	A549 ^[b]^		OVCAR-3 ^[c]^		HCT-116 ^[d]^		MCF-7 ^[e]^
**11a**	9.50 ± 1.23		13.61 ± 2.45		10.33 ± 2.74		15.46 ± 2.53
**11b**	**1.95 ± 0.33**		**4.02 ± 0.24**		**7.45 ± 1.98**		**9.21 ± 2.54**
**11c**	4.29 ± 0.56		7.65 ± 2.56		12.57 ± 2.43		7.60 ± 1.96
**11d**	4.88 ± 1.18		17.14 ± 1.50		8.76 ± 3.11		16.16 ± 3.88
**11e**	15.61 ± 2.80		7.95 ± 3.35		9.22 ± 4.57		9.33 ± 4.08
**11f**	11.83 ± 1.07		21.65 ± 8.94		12.21 ± 2.01		8.94 ± 3.17
**11 g**	6.84 ± 1.68		4.64 ± 0.99		9.81 ± 2.89		7.96 ± 1.10
**11 h**	15.14 ± 1.18		4.44 ± 0.10		8.97 ± 3.16		6.50 ± 1.78
**11i**	13.59 ± 2.47		4.28 ± 0.77		10.76 ± 2.95		6.34 ± 3.17
**11j**	18.82 ± 2.67		5.79 ± 1.25		17.93 ± 1.95		5.27 ± 0.92
**11k**	12.55 ± 5.91		10.50 ± 0.09		13.05 ± 2.03		7.54 ± 1.05
**11 L**	11.05 ± 2.98		6.14 ± 0.56		11.72 ± 4.88		8.54 ± 1.65
**11 m**	8.70 ± 4.10		6.00 ± 1.83		5.87 ± 0.54		9.33 ± 1.33
**11n**	17.92 ± 3.94		12.61 ± 1.55		15.67 ± 1.98		10.60 ± 0.85
**11o**	7.40 ± 1.75		4.20 ± 0.48		10.82 ± 2.11		7.99 ± 2.53
**11p**	9.58 ± 2.53		12.69 ± 1.43		9.93 ± 3.01		16.41 ± 4.37
**11q**	10.62 ± 1.20		13.18 ± 4.09		> 50		8.72 ± 0.71
**11r**	11.86 ± 5.74		3.90 ± 0.26		12.08 ± 0.98		> 50
**11s**	> 50		> 50		> 50		> 50
**11t**	> 50		> 50		> 50		> 50
**11u**	> 50		> 50		> 50		> 50
**11v**	> 50		> 50		> 50		> 50
**Rucaparib**	4.91 ± 0.11		2.41 ± 0.13		13.51 ± 0.17		12.98 ± 0.28

[a] IC_50_ values reported as an average ≥ 3 determinations with standard deviation (SD) reported (IC_50_ = Mean ± SD); [b] human lung adenocarcinoma cell line; [c] human ovarian cancer cell line; [d] human colon cancer cell line; [e] human breast cancer cell line.

These results showed that compound **11b** had excellent anti-proliferative activity against A549 cells (IC_50_ = 1.95 ± 0.33 µM). The compounds **11t-11v** (CN substitution) showed the worst anti-proliferative activity. We believed that it was related to the massive steric hindrance of the CN. Then, we found that compound **11b-11d** (F substitution) exhibited better proliferative inhibitory activity than compound **11 h-11j** (Cl substitution) and compound **11n-11p** (Br substitution) when R was replaced by halogen, especially against A549 cells.

Simultaneously, when R was replaced by CH_3_ and OCH_3_, we found that the proliferative inhibitory activity of the para-substituted compounds (**11 g, 11 m**) was better than that of the ortho-substituted (**11e, 11k**) and meso-substituted compounds (**11f, 11 L**). When R was OH (**11q-11s**), the proliferation inhibition activity of the compounds was weaker than that of CH_3_ (**11e-11 g**) and OCH_3_ (**11k-11 m**). In conclusion, these findings provide new insight for the future design of novel PARP-1 inhibitors.

#### The SI of compound **11b**

MTT results showed compound **11b** had the most prominent effect among the target compounds. We then further evaluated the low toxicity of compound **11b** by the SI, and the results were indicated in Table 2. Compound **11b** had a better effect for HPAEpiC (SI = 15.38), and the value was better than rucaparib.

**Table 2:** The SI of compound **11b**

Compounds	SI ^[f]^			
	HPAEpiC ^[g]^	IOSE80 ^[h]^	HIEC ^[i]^	HTB-125 ^[j]^
**11b**	15.38 ± 0.97	10.57 ± 2.01	6.12 ± 1.76	7.06 ± 1.32
**Rucaparib**	8.32 ± 1.43	11.86 ± 1.88	3.98 ± 0.89	4.32 ± 1.51

[f] SI = CC_50_/IC_50_; Values are presented as means ± SD of at least three independent determinations; [g] Human normal alveolar epithelial cells; [h] Human normal ovarian epithelial cells; [i] Human normal intestinal epithelial cells; [j] Human normal breast cell

#### In vitro inhibitory activity against PARP-1

Considering that the compound **11b** had excellent anti-proliferative activities and low toxicity at the cellular level, we evaluated the ability of compound **11b** to inhibit PARP-1/2 enzyme activity in vitro. In addition, rucaparib served as a control.

The results indicated that compound **11b** had a slightly better inhibitory effect on PARP-1 than rucaparib, and compound **11b** had a selective inhibitory effect between PARP-1/2 (Table 3).

**Table 3:** In vitro inhibitory activity of compound **11b** against PARP-1/2.

Compounds	IC_50_^[a]^ (nM)	
	PARP-1	PARP-2
**11b**	19.24 ± 1.63	32.58 ± 1.97
**Rucaparib**	23.88 ± 2.90	25.79 ± 3.17

[a] IC_50_ values reported as an average ≥ 3 determinations with standard deviation (SD) reported (IC_50_ = Mean ± SD)

#### Molecular docking and molecular dynamics of compound **11b**

In order to more directly observe the vital structural characteristics and binding stability of compound **11b** and PARP-1, molecular docking studies (PDB code: 4BJC) and molecular dynamics were carried out.

**Fig. 3:**
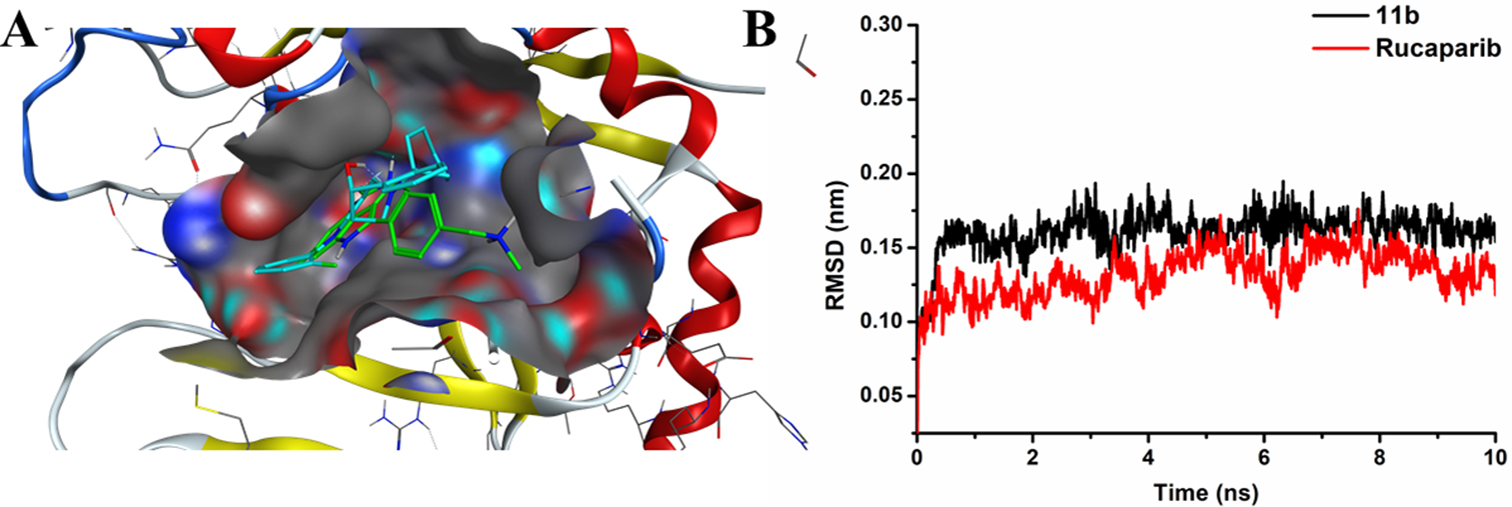
Molecular docking and molecular dynamics of rucaparib and compound **11b**. **A**: Superimposition of the binding models of compound **11b** and rucaparib with PARP-1 (Blue: compound **11b**; Green: rucaparib). **B**: The molecular dynamics results of compound **11b** and rucaparib were expressed by RMSD value

The molecular docking studies showed that compound **11b** could enter the active pocket, and fully occupy the active pocket (Fig. 3A). The molecular dynamics results indicated that compound **11b** binds stably at the active site. The Root Mean Square Deviation (RMSD) value of compound **11b** fluctuates less than rucaparib (Fig. 3B).

#### Effects of compound **11b** on the apoptosis in A549 cells

**Fig. 4:**
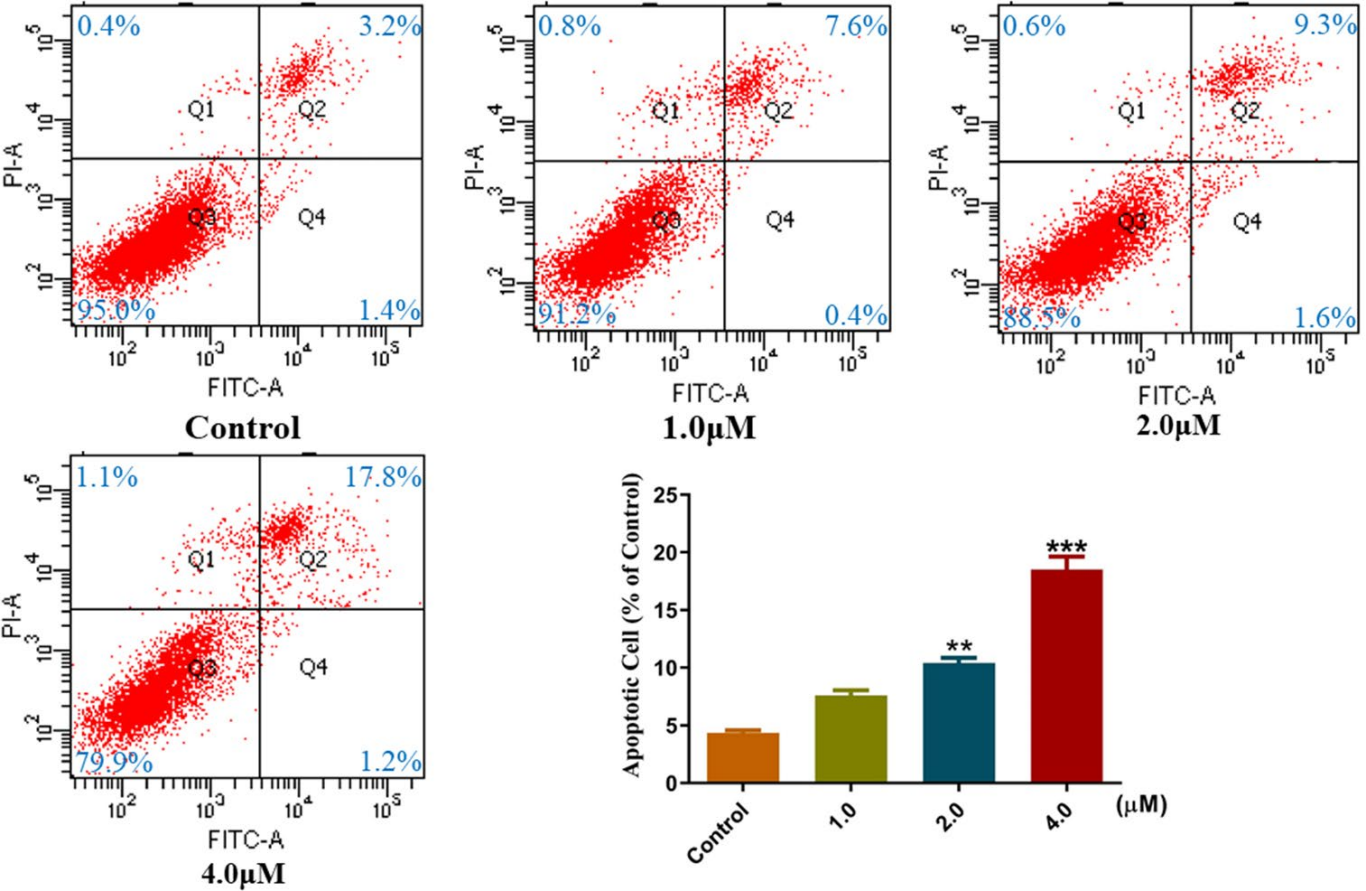
Effects of compound 11b on the apoptosis in A549 cells. Cell apoptosis analysis was assessed in A549 cells during incubation with different concentrations for 48 h. Q1: Necrotic cell; Q2: Late apoptotic cell; Q3: Early apoptotic cell; Q4: Normal cell. Data were expressed as an average ≥ 3 determinations with standard deviation reported (**P < 0.01 and ***P < 0.001. Compound **11b**-treated group VS control)

PARP-1 inhibitors have been reported to induce further apoptosis of cancer cells [29, 30], which was also confirmed by our previous studies [26, 31, 32]. An annexin-V/PI binding assay was conducted in A549 cells to evaluate the capacity of compound **11b** to induce apoptosis in cancer cells. Flow cytometry analysis showed that the apoptosis rates of A549 cells treated with different concentrations of compound **11b** were 4.6%, 8.0%, 10.9% and 19.0%, respectively. (Fig. 4).

This suggested that compound **11b** could induce apoptosis in a dose-dependent manner. And we found that the effect of late apoptosis was better. The result is significantly different (P < 0.001).

#### Effects of compound **11b** on PAR and the expression of apoptosis-related protein in A549 cells

**Fig. 5:**
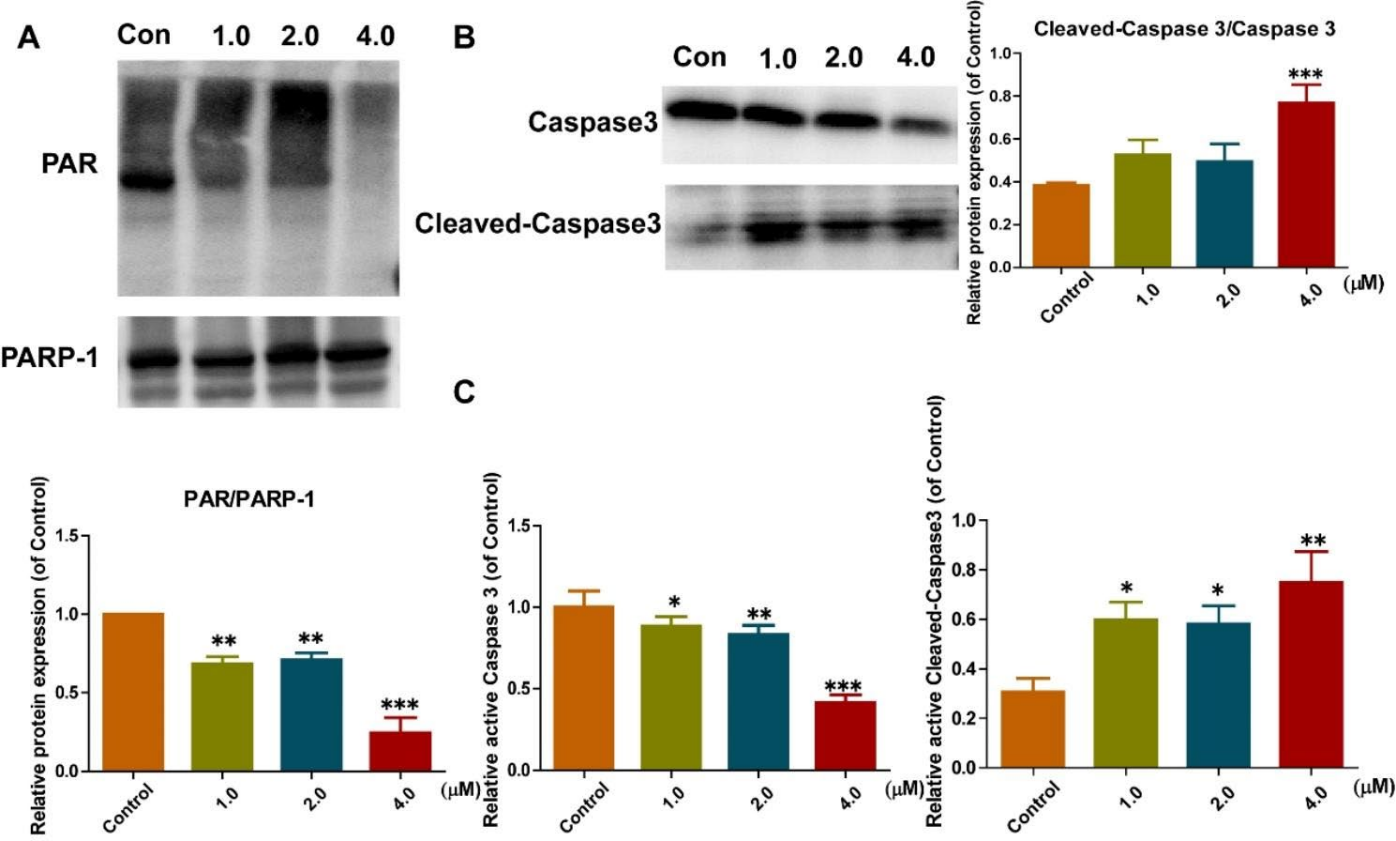
Western blot analysis: **A**: Effects of compound **11b** on PAR in A549 cells. **B, C**: Effect of compound **11b** on Caspase 3 and Cleaved-Caspase 3 in A549 cells. Data were expressed as an average ≥ 3 determinations with standard deviation reported (*P < 0.05; **P < 0.01 and ***P < 0.001. Compound **11b** -treated group VS control)

PARylation is a critical post-translational modification in which ADP-ribose units are added to a wide array of target proteins by PARP-1 [33–35]. PAR is the active product of PARP-1, and the biosynthesis of PAR will be reduced while PARP-1 is inhibited. Here, we used the western blot to evaluate PAR levels and PARP-1 expression. As shown in Fig. 5A, compound **11b** could reduce the biosynthesis of PAR, and had the best effect at 4.0 µM.

Meanwhile, we also detected the expression of Cleaved-Caspase 3 and Caspase 3 after different treatment concentrations to explore the mechanism of compound **11b** inducing cell apoptosis. The results showed that the ratio of Cleaved-Caspase 3/Caspase 3 was best at 4.0 µM. The expression of Caspase 3 decreased with the increase of compound **11b** concentration **(**Fig. 5B, C**)**.

These results demonstrated that compound **11b** could reduce the biosynthesis of PAR, and induce apoptosis in A549 cells. The result is significantly different (P < 0.001).

#### Prediction of ADMET bioactivity of compound **11b**

With the progress of science and technology, the application of computers makes the related research of drug metabolism more convenient and efficient [36]. So, we evaluated the ADMET properties of compound **11b** using relevant websites, such as AdmetSAR and SwissADME, and summarized the results in Table 4.

The prediction results indicated compound **11b** and rucaparib had similar pharmacokinetic characteristics. The reference values of corresponding indexes were relatively close. Therefore, the above results also provide theoretical support for further study of the activity of compound **11b** in vivo.

**Table 4:** Pharmacokinetic prediction of compound **11b**

Compound	Water solubility ^[k]^	Plasma protein binding ^[l]^	Acute oral toxicity ^[m]^	Tetrahymena pyriformis ^[n]^	Bioavailability Score
**11b**	**-3.606**	**0.781**	**2.752**	**1.979**	**0.55**
**Rucaparib**	**-3.088**	**0.643**	**2.673**	**1.429**	**0.55**

[k] Unit: logS; [l] Unit: 100%; [m] Unit: log(1/(mol/kg)); [n] Unit: pIGC_50_ (µg/L)

## Conclusion

This study designed and identified a series of novel 2,3,4,5-tetrahydrospiro[benzo[*c*]azepine-1,1’-cyclohexan]-5-ol derivatives as potent PARP-1 inhibitors. We performed the MTT assay on 22 target compounds and found that compound **11b** had better anti-proliferation activity and SI value on lung cancer cells. In addition, compound **11b** had better PARP-1/2 enzyme activity than rucaparib. Moreover, molecular docking and molecular dynamics studies showed that compound **11b** could fully occupy the active pocket and bind stably at the active site. Mechanistically, flow cytometry indicated that compound **11b** could effectively induce apoptosis of A549 cells. Western blot analysis demonstrated that compound **11b** could reduce the biosynthesis of PAR, and up-regulate the ratio of Cleaved-Caspase 3/Caspase 3. Afterward, the results of pharmacokinetic prediction showed that compound **11b** had similar properties to rucaparib, which provided theoretical support for further study.

In conclusion, compound **11b** could offer a significant guiding effect for further research on novel PARP-1 inhibitors and might open new horizons for discovering more potent PARP-1 inhibitors.

## Experimental procedures

### General methods

All solvents and reagents were commercially available, and were used without further purification unless stated. The progress of the reactions was monitored by thin-layer chromatography on a glass plate coated with silica gel with a fluorescent indicator (GF254, Qingdao Ocean Chemicals, China). The melting point of target compounds were detected on the RD-1 melting apparatus (Tianjin Guoming Medical Equipment Co., LTD, China). The ^1^ H and ^13^ C nuclear magnetic resonance (NMR) spectra were recorded on a model 600 Bruker Avance spectrometer (Bruker, Germany) at 600 and 150 MHz, respectively. Chemical shifts are given in parts per million (*δ*) referenced to DMSO-*d*_*6*_ at *δ* 2.50 for ^1^ H and *δ* 39.5 for ^13^ C. High-resolution mass spectra (HRMS) of target compounds were performed by a Waters Q-TOF Premier spectrometer (Waters, USA).

#### *The synthesis of 2-(prop-2-yn-1-yl)-3,4-dihydrospiro[benzo[c]azepine-1,1’-cyclohexan]-5(2 H)-one (*compound **9***)*

The preparation conditions of compound **9** had been reported in our previous study [26]. At the same time, we also gave a detailed description of it in the supplementary materials 1.

#### *The synthesis of 2-(prop-2-yn-1-yl)-2,3,4,5-tetrahydrospiro[benzo[c]azepine-1,1’-cyclohexan]-5-ol (*compound **10***)*

Compound **9** (2.00 g, 0.007 mol) and lithium aluminum hydride (0.57 g, 0.012 mol) were added to tetrahydrofuran at -5℃, and then heated to 60℃ for 12 h to obtain compound **10**. The preparation process mainly refers to the treatment method of compound **4** [26]. Detailed information was presented in the supplementary materials 1.

#### The synthesis of target compounds **11a-11v**

The preparation process of the target compounds **11a-11v** was similar to the method reported in our previous study [26, 31]. And they were also explained in detail in the supplementary materials 1.

##### 2-((1-phenyl-1 H-1,2,3-triazol-4-yl)methyl)-2,3,4,5-tetrahydrospiro[benzo[c]azepine-1,1’-cyclohexan]-5-ol (**11a**)

A white solid, yield: 75.91%. Mp: 164.0-164.7℃. ^1^ H NMR (600 MHz, DMSO-*d*_*6*_) *δ* 7.95 (s, 1 H), 7.89 (dd, *J* = 8.6, 1.0 Hz, 1 H), 7.61–7.53 (m, 3 H), 7.46 (m, 3 H), 7.38–7.32 (m, 1 H), 7.25–7.17 (m, 1 H), 3.33 (s, 1 H), 2.89 (s, 2 H), 2.73 (s, 2 H), 1.64 (d, *J* = 11.5 Hz, 2 H), 1.45 (d, *J* = 10.9 Hz, 2 H), 1.22 (m, 8 H); ^13^ C NMR (150 MHz, DMSO-*d*_*6*_) *δ* 147.62, 142.27, 138.46, 137.23, 134.55, 130.26, 128.81, 126.53, 123.60, 121.77, 121.21, 120.31, 69.94, 65.25, 62.45, 60.23, 40.40, 40.26, 40.12, 39.98, 39.84, 39.70, 39.56, 36.24, 31.22, 26.43, 14.55. ESI-HRMS calcd. for C_24_H_29_N_4_O [M + H]^+^ 389.2263, found: 389.2324.

##### 2-((1-(2-fluorophenyl)-1 H-1,2,3-triazol-4-yl)methyl)-2,3,4,5-tetrahydrospiro[benzo[c]azepine-1,1’-cyclohexan]-5-ol (**11b**)

A brown solid, yield: 77.93%. Mp: 166.6-166.9℃. ^1^ H NMR (600 MHz, DMSO-*d*_*6*_) *δ* 7.82–7.79 (m, 3 H), 7.42 (td, *J* = 7.9, 1.2 Hz, 3 H), 7.36–7.34 (m, 3 H), 2.88 (s, 1 H), 2.73–2.72 (m, 2 H), 1.98 (s, 2 H), 1.63 (d, *J* = 12.1 Hz, 2 H), 1.48 (m, 3 H), 1.29–1.15 (m, 7 H); ^13^ C NMR (150 MHz, DMSO-*d*_*6*_) *δ* 161.22 (d, *J* = 238.9 Hz), 155.05, 153.39, 147.18, 146.20, 143.14, 126.53, 126.35, 125.96, 125.94, 125.11, 125.09, 117.62, 117.49, 71.00, 66.30, 62.45, 60.23, 29.70, 26.41, 22.25, 14.55. ESI-HRMS calcd. for C_24_H_28_FN_4_O [M + H]^+^ 407.2149, found: 407.2254.

##### 2-((1-(3-fluorophenyl)-1 H-1,2,3-triazol-4-yl)methyl)-2,3,4,5-tetrahydrospiro[benzo[c]azepine-1,1’-cyclohexan]-5-ol (**11c**)

A brown solid, yield: 79.81%. Mp: 167.1-167.8℃. ^1^ H NMR (600 MHz, DMSO-*d*_*6*_) *δ* 7.88–7.75 (m, 2 H), 7.63 (td, *J* = 8.3, 6.4 Hz, 2 H), 7.39–7.27 (m, 2 H), 7.22 (s, 3 H), 5.33 (s, 1 H), 3.34 (s, 2 H), 1.99 (s, 2 H), 1.64 (dd, *J* = 16.8, 9.4 Hz, 3 H), 1.48 (m, 4 H), 1.32–1.13 (m, 5 H); ^13^ C NMR (150 MHz, DMSO-*d*_*6*_) *δ* 162.91 (d, *J* = 244.8 Hz), 147.81, 138.50, 138.43, 132.25, 132.19, 126.55, 121.99, 116.18, 116.16, 115.57, 115.43, 107.82, 107.65, 62.46, 60.23, 26.43, 22.26, 21.92, 21.23, 19.13, 14.56. ESI-HRMS calcd. for C_24_H_28_FN_4_O [M + H]^+^ 407.2149, found: 407.2247.

##### 2-((1-(4-fluorophenyl)-1 H-1,2,3-triazol-4-yl)methyl)-2,3,4,5-tetrahydrospiro[benzo[c]azepine-1,1’-cyclohexan]-5-ol (**11d**)

A brown solid, yield: 80.10%. Mp: 166.2-166.8℃. ^1^ H NMR (600 MHz, DMSO-*d*_*6*_) *δ* 7.97–7.91 (m, 3 H), 7.42–7.37 (m, 3 H), 7.32 (d, *J* = 8.3 Hz, 1 H), 7.19 (d, *J* = 6.4 Hz, 2 H), 2.87 (s, 1 H), 2.72 (s, 2 H), 1.97 (s, 2 H), 1.62 (d, *J* = 11.5 Hz, 2 H), 1.45 (m, 3 H), 1.28–1.12 (m, 7 H); ^13^ C NMR (150 MHz, DMSO-*d*_*6*_) *δ* 161.91 (d, *J* = 245.7 Hz), 147.66, 145.23, 143.12, 133.81, 133.79, 126.52, 122.56, 122.50, 121.94, 117.08, 116.92, 62.46, 60.21, 36.20, 31.18, 26.43, 22.27, 21.15, 14.49. ESI-HRMS calcd. for C_24_H_28_FN_4_O [M + H]^+^ 407.2149, found: 407.2234.

##### 2-((1-(o-tolyl)-1 H-1,2,3-triazol-4-yl)methyl)-2,3,4,5-tetrahydrospiro[benzo[c]azepine-1,1’-cyclohexan]-5-ol (**11e**)

An off-white solid, yield: 76.19%. Mp: 165.2–166.0℃. ^1^ H NMR (600 MHz, DMSO-*d*_*6*_) *δ* 7.48–7.43 (m, 3 H), 7.38 (m, 4 H), 7.21 (s, 2 H), 2.89 (s, 1 H), 2.73 (s, 2 H), 2.13 (s, 2 H), 1.99 (s, 3 H), 1.63 (d, *J* = 11.8 Hz, 2 H), 1.45 (s, 2 H), 1.29–1.15 (m, 8 H); ^13^ C NMR (150 MHz, DMSO-*d*_*6*_) *δ* 162.77, 154.71, 146.63, 143.37, 140.10, 136.95, 135.52, 133.43, 131.74, 130.03, 128.89, 127.37, 126.43, 125.27, 73.04, 69.93, 62.46, 60.23, 36.25, 31.23, 26.43, 17.89, 14.55. ESI-HRMS calcd. for C_25_H_31_N_4_O [M + H]^+^ 403.2420, found: 403.2481.

##### 2-((1-(m-tolyl)-1 H-1,2,3-triazol-4-yl)methyl)-2,3,4,5-tetrahydrospiro[benzo[c]azepine-1,1’-cyclohexan]-5-ol (**11f**)

An off-white solid, yield: 78.23%. Mp: 167.3-168.2℃. ^1^ H NMR (600 MHz, DMSO-*d*_*6*_) *δ* 7.74–7.67 (m, 3 H), 7.41 (t, *J* = 7.8 Hz, 2 H), 7.33 (d, *J* = 7.9 Hz, 1 H), 7.22 (m, 3 H), 2.87 (s, 1 H), 2.73 (s, 2 H), 2.38 (s, 2 H), 1.97 (s, 3 H), 1.63 (d, *J* = 10.9 Hz, 2 H), 1.46 (m, 2 H), 1.30–1.12 (m, 8 H); ^13^ C NMR (150 MHz, DMSO-*d*_*6*_) *δ* 161.75, 154.93, 146.57, 144.03, 142.22, 138.98, 136.25, 132.61, 129.01, 128.36, 125.53, 120.65, 119.70, 116.39, 68.95, 64.05, 61.48, 59.25, 35.24, 30.22, 25.49, 20.38, 13.54. ESI-HRMS calcd. for C_25_H_31_N_4_O [M + H]^+^ 403.2420, found: 403.2480.

##### 2-((1-(p-tolyl)-1 H-1,2,3-triazol-4-yl)methyl)-2,3,4,5-tetrahydrospiro[benzo[c]azepine-1,1’-cyclohexan]-5-ol (**11 g**)

An off-white solid, yield: 77.22%. Mp: 164.3-165.2℃. ^1^ H NMR (600 MHz, DMSO-*d*_*6*_) *δ* 7.76 (d, *J* = 8.4 Hz, 1 H), 7.34 (t, *J* = 9.0 Hz, 4 H), 7.20 (s, 4 H), 2.87 (s, 1 H), 2.74–2.71 (m, 2 H), 2.35 (s, 3 H), 1.98 (s, 2 H), 1.63 (d, *J* = 11.6 Hz, 2 H), 1.46 (m, 3 H), 1.29–1.13 (m, 7 H); ^13^ C NMR (150 MHz, DMSO-*d*_*6*_) *δ* 161.72, 154.63, 151.99, 146.46, 143.42, 141.39, 137.30, 133.99, 129.54, 125.48, 120.56, 119.14, 69.13, 64.76, 61.40, 59.19, 35.20, 30.19, 25.41, 19.98, 13.51. ESI-HRMS calcd. for C_25_H_31_N_4_O [M + H]^+^ 403.2420, found: 403.2492.

##### 2-((1-(2-chlorophenyl)-1 H-1,2,3-triazol-4-yl)methyl)-2,3,4,5-tetrahydrospiro[benzo[c]azepine-1,1’-cyclohexan]-5-ol (**11 h**)

A yellow solid, yield: 80.17%. Mp: 167.9-168.3℃. ^1^ H NMR (600 MHz, DMSO*-d*_*6*_) *δ* 7.37–7.26 (m, 5 H), 7.20 (s, 2 H), 6.84 (dd, *J* = 8.1, 1.4 Hz, 2 H), 2.88 (s, 1 H), 2.72 (s, 2 H), 1.98 (s, 2 H), 1.64 (d, *J* = 11.7 Hz, 2 H), 1.47 (m, 2 H), 1.20 (m, 8 H); ^13^ C NMR (150 MHz, DMSO*-d*_*6*_) *δ* 162.77, 158.88, 147.48, 146.06, 143.18, 138.22, 131.11, 126.52, 121.73, 119.03, 115.76, 110.71, 107.24, 103.45, 70.17, 65.78, 62.44, 60.23, 36.25, 31.23, 26.43, 14.55. ESI-HRMS calcd. for C_24_H_28_ClN_4_O [M + H]^+^ 423.1873, found: 423.1952.

##### 2-((1-(3-chlorophenyl)-1 H-1,2,3-triazol-4-yl)methyl)-2,3,4,5-tetrahydrospiro[benzo[c]azepine-1,1’-cyclohexan]-5-ol (**11i**)

A yellow solid, yield: 81.23%. Mp: 168.1-168.8℃. ^1^ H NMR (600 MHz, DMSO*-d*_*6*_) *δ* 7.97–7.90 (m, 3 H), 7.61 (t, *J* = 8.1 Hz, 2 H), 7.54 (m, 2 H), 7.39–7.33 (m, 1 H), 7.22 (m, 1 H), 2.89 (s, 1 H), 2.75–2.71 (m, 2 H), 1.99 (s, 2 H), 1.66 (d, *J* = 12.0 Hz, 2 H), 1.48 (s, 3 H), 1.30–1.15 (m, 7 H); ^13^ C NMR (150 MHz, DMSO*-d*_*6*_) *δ* 162.78, 158.75, 154.61, 151.73, 147.81, 143.71, 138.26, 134.62, 132.03, 128.62, 126.55, 122.02, 120.04, 118.87, 70.17, 65.26, 62.46, 60.23, 36.26, 31.24, 26.43, 14.56. ESI-HRMS calcd. for C_24_H_28_ClN_4_O [M + H]^+^ 423.1873, found: 423.1960.

##### 2-((1-(4-chlorophenyl)-1 H-1,2,3-triazol-4-yl)methyl)-2,3,4,5-tetrahydrospiro[benzo[c]azepine-1,1’-cyclohexan]-5-ol (**11j**)

A yellow solid, yield: 81.91%. Mp: 165.6-166.3℃. ^1^ H NMR (600 MHz, DMSO*-d*_*6*_) *δ* 7.98–7.91 (m, 3 H), 7.64–7.58 (m, 3 H), 7.36–7.30 (m, 1 H), 7.24–7.17 (m, 2 H), 2.87 (s, 1 H), 2.73 (s, 2 H), 1.97 (s, 2 H), 1.62 (d, *J* = 11.9 Hz, 2 H), 1.43 (m, 4 H), 1.30–1.12 (m, 6 H); ^13^ C NMR (150 MHz, DMSO*-d*_*6*_) *δ* 162.71, 159.30, 153.54, 147.82, 145.01, 142.96, 136.02, 133.03, 130.15, 126.50, 121.87, 121.77, 62.43, 60.20, 54.89, 50.19, 36.20, 31.17, 26.44, 14.49. ESI-HRMS calcd. for C_24_H_28_ClN_4_O [M + H]^+^ 423.1873, found: 423.1948.

##### 2-((1-(2-methoxyphenyl)-1 H-1,2,3-triazol-4-yl)methyl)-2,3,4,5-tetrahydrospiro[benzo[c]azepine-1,1’-cyclohexan]-5-ol (**11k**)

A light yellow solid, yield: 82.38%. Mp: 171.1-171.9℃. ^1^ H NMR (600 MHz, DMSO*-d*_*6*_) *δ* 7.61–7.57 (m, 2 H), 7.53–7.49 (m, 2 H), 7.37–7.33 (m, 1 H), 7.29 (dd, *J* = 8.4, 0.9 Hz, 1 H), 7.21 (d, *J* = 3.3 Hz, 1 H), 7.13 (dd, *J* = 7.7, 1.1 Hz, 2 H), 3.83–3.80 (m, 2 H), 2.89 (s, 1 H), 2.73 (d, *J* = 0.4 Hz, 2 H), 1.99 (s, 3 H), 1.68–1.61 (m, 3 H), 1.48 (m, 4 H), 1.31–1.15 (m, 5 H); ^13^ C NMR (150 MHz, DMSO*-d*_*6*_) *δ* 162.76, 151.96, 146.31, 143.41, 139.02, 134.86, 130.89, 126.50, 126.39, 126.05, 125.49, 121.30, 116.92, 113.43, 63.56, 62.43, 60.23, 56.54, 36.24, 26.43, 22.25, 21.22, 14.55. ESI-HRMS calcd. for C_25_H_31_N_4_O_2_ [M + H]^+^ 419.2369, found: 419.2440.

##### 2-((1-(3-methoxyphenyl)-1 H-1,2,3-triazol-4-yl)methyl)-2,3,4,5-tetrahydrospiro[benzo[c]azepine-1,1’-cyclohexan]-5-ol (**11 L**)

A light yellow solid, yield: 83.44%. Mp: 172.3-174.1℃. ^1^ H NMR (600 MHz, DMSO*-d*_*6*_) *δ* 7.50–7.42 (m, 3 H), 7.34 (d, *J* = 8.7 Hz, 2 H), 7.20 (d, *J* = 4.3 Hz, 2 H), 7.01 (m, 2 H), 3.83 (s, 3 H), 2.87 (s, 1 H), 2.72 (s, 2 H), 1.97 (s, 2 H), 1.63 (d, *J* = 12.2 Hz, 2 H), 1.46 (m, 2 H), 1.28–1.13 (m, 8 H); ^13^ C NMR (150 MHz, DMSO*-d*_*6*_) *δ* 161.75, 159.64, 153.62, 146.55, 142.73, 137.32, 133.64, 130.16, 125.53, 120.86, 116.78, 113.43, 111.29, 104.94, 63.74, 61.48, 59.23, 55.03, 35.23, 30.21, 25.45, 20.20, 13.53. ESI-HRMS calcd. for C_25_H_31_N_4_O_2_ [M + H]^+^ 419.2369, found: 419.2435.

##### 2-((1-(4-methoxyphenyl)-1 H-1,2,3-triazol-4-yl)methyl)-2,3,4,5-tetrahydrospiro[benzo[c]azepine-1,1’-cyclohexan]-5-ol (**11 m**)

A light yellow solid, yield: 83.66%. Mp: 170.2-172.3℃. ^1^ H NMR (600 MHz, DMSO*-d*_*6*_) *δ* 7.79 (d, *J* = 9.0 Hz, 1 H), 7.36–7.33 (m, 3 H), 7.21 (d, *J* = 4.0 Hz, 4 H), 7.10 (d, *J* = 9.0 Hz, 1 H), 3.82 (s, 3 H), 2.89 (s, 2 H), 2.73 (s, 2 H), 1.98 (s, 1 H), 1.68–1.61 (m, 2 H), 1.46 (s, 2 H), 1.30–1.14 (m, 8 H); ^13^ C NMR (150 MHz, DMSO*-d*_*6*_) *δ* 162.76, 159.49, 152.60, 150.11, 141.50, 138.31, 130.71, 129.13, 124.73, 121.96, 117.57, 115.22, 65.49, 62.43, 60.23, 55.99, 36.24, 31.22, 19.13, 14.55, 14.02. ESI-HRMS calcd. for C_25_H_31_N_4_O_2_ [M + H]^+^ 419.2369, found: 419.2410.

##### 2-((1-(2-bromophenyl)-1 H-1,2,3-triazol-4-yl)methyl)-2,3,4,5-tetrahydrospiro[benzo[c]azepine-1,1’-cyclohexan]-5-ol (**11n**)

A brown solid, yield: 79.22%. Mp: 168.2-169.8℃. ^1^ H NMR (600 MHz, DMSO-*d*_*6*_) *δ* 7.87 (d, J = 7.7 Hz, 1 H), 7.64–7.49 (m, 4 H), 7.40–7.33 (m, 2 H), 7.27–7.18 (m, 2 H), 3.47 (s, 1 H), 3.37 (s, 2 H), 1.98 (s, 2 H), 1.64 (d, J = 11.7 Hz, 2 H), 1.47 (s, 2 H), 1.22 (m, 8 H); ^13^ C NMR (150 MHz, DMSO-*d*_*6*_) *δ* 146.61, 144.75, 142.43, 139.05, 136.91, 134.02, 132.10, 129.30, 129.05, 126.50, 125.74, 122.79, 120.45, 119.23, 70.09, 67.01, 62.48, 60.21, 26.47, 22.29, 21.18, 14.52. ESI-HRMS calcd. for C_24_H_28_BrN_4_O [M + H]^+^ 467.1368, found: 467.1435.

##### 2-((1-(3-bromophenyl)-1 H-1,2,3-triazol-4-yl)methyl)-2,3,4,5-tetrahydrospiro[benzo[c]azepine-1,1’-cyclohexan]-5-ol (**11o**)

A brown solid, yield: 80.17%. Mp: 169.3-170.2℃. ^1^ H NMR (600 MHz, DMSO-*d*_*6*_) *δ* 7.80–7.75 (m, 2 H), 7.69–7.63 (m, 3 H), 7.39–7.32 (m, 2 H), 7.26–7.17 (m, 2 H), 2.89 (s, 1 H), 2.73 (s, 2 H), 1.99 (s, 2 H), 1.65 (d, J = 9.6 Hz, 2 H), 1.47 (s, 2 H), 1.30–1.15 (m, 8 H); ^13^ C NMR (150 MHz, DMSO-*d*_*6*_) *δ* 162.76, 147.81, 138.34, 136.02, 133.14, 132.23, 131.53, 130.22, 126.54, 122.89, 122.75, 121.97, 119.24, 118.84, 69.64, 65.78, 62.45, 60.23, 36.25, 31.23, 21.23, 14.55. ESI-HRMS calcd. for C_24_H_28_BrN_4_O [M + H]^+^ 467.1368, found: 467.1442.

##### 2-((1-(4-bromophenyl)-1 H-1,2,3-triazol-4-yl)methyl)-2,3,4,5-tetrahydrospiro[benzo[c]azepine-1,1’-cyclohexan]-5-ol (**11p**)

A brown solid, yield: 79.86%. Mp: 167.1-166.3℃. ^1^ H NMR (600 MHz, DMSO*-d*_*6*_) *δ* 7.95 (s, 1 H), 7.88 (d, *J* = 8.8 Hz, 2 H), 7.76 (d, *J* = 8.8 Hz, 2 H), 7.37–7.30 (m, 2 H), 7.25–7.16 (m, 2 H), 2.88 (s, 1 H), 2.72 (s, 2 H), 1.98 (s, 2 H), 1.63 (d, *J* = 12.5 Hz, 2 H), 1.45 (s, 3 H), 1.28–1.12 (m, 7 H); ^13^ C NMR (150 MHz, DMSO*-d*_*6*_) *δ* 162.75, 147.83, 143.00, 138.28, 136.42, 133.13, 132.20, 130.21, 126.53, 122.19, 121.80, 121.38, 69.95, 65.54, 62.44, 60.22, 36.24, 31.22, 26.44, 14.54. ESI-HRMS calcd. for C_24_H_28_BrN_4_O [M + H]^+^ 467.1368, found: 467.1447.

##### 2-((1-(2-hydroxyphenyl)-1 H-1,2,3-triazol-4-yl)methyl)-2,3,4,5-tetrahydrospiro[benzo[c]azepine-1,1’-cyclohexan]-5-ol (**11q**)

A brown solid, yield: 83.42%. Mp: 156.8-157.2℃. ^1^ H NMR (600 MHz, DMSO*-d*_*6*_) *δ* 7.51 (m, 2 H), 7.37–7.34 (m, 2 H), 7.30 (dd, *J* = 8.4, 0.9 Hz, 2 H), 7.21 (s, 1 H), 7.13 (m, 2 H), 3.83 (s, 1 H), 2.69 (s, 2 H), 1.99 (s, 2 H), 1.64 (d, *J* = 12.3 Hz, 2 H), 1.48 (m, 3 H), 1.22 (m, 7 H); ^13^ C NMR (150 MHz, DMSO*-d*_*6*_) *δ* 162.78, 158.46, 151.99, 146.30, 140.84, 135.62, 130.92, 126.51, 126.38, 126.09, 125.53, 121.31, 118.21, 113.45, 66.32, 62.43, 60.23, 56.56, 36.26, 31.24, 26.42, 14.56. ESI-HRMS calcd. for C_24_H_29_N_4_O_2_ [M + H]^+^ 405.2212, found: 405.2276.

##### 2-((1-(3-hydroxyphenyl)-1 H-1,2,3-triazol-4-yl)methyl)-2,3,4,5-tetrahydrospiro[benzo[c]azepine-1,1’-cyclohexan]-5-ol (**11r**)

A brown solid, yield: 82.94%. Mp: 157.4-158.6℃. ^1^ H NMR (600 MHz, DMSO*-d*_*6*_) *δ* 7.37–7.23 (m, 5 H), 7.23–7.10 (m, 4 H), 2.89 (s, 1 H), 2.73 (s, 2 H), 1.99 (s, 2 H), 1.65 (m, 2 H), 1.27–1.16 (m, 6 H), 0.93–0.83 (m, 4 H); ^13^ C NMR (150 MHz, DMSO*-d*_*6*_) *δ* 162.77, 150.59, 143.14, 140.09, 138.43, 132.00, 129.14, 127.23, 121.37, 121.35, 115.93, 113.53, 113.47, 109.09, 74.12, 65.51, 65.49, 56.61, 36.25, 31.23, 30.47, 19.13. ESI-HRMS calcd. for C_24_H_29_N_4_O_2_ [M + H]^+^ 405.2212, found: 405.2278.

##### 2-((1-(4-hydroxyphenyl)-1 H-1,2,3-triazol-4-yl)methyl)-2,3,4,5-tetrahydrospiro[benzo[c]azepine-1,1’-cyclohexan]-5-ol (**11s**)

A brown solid, yield: 84.00%. Mp: 171.1-172.3℃. ^1^ H NMR (600 MHz, DMSO*-d*_*6*_) *δ* 7.68–7.64 (m, 3 H), 7.38–7.32 (m, 3 H), 7.21 (s, 3 H), 6.95–6.89 (m, 3 H), 2.89 (s, 1 H), 2.75–2.71 (m, 2 H), 1.99 (s, 2 H), 1.64 (d, *J* = 11.9 Hz, 2 H), 1.47 (m, 5 H), 1.27 (m, 5 H); ^13^ C NMR (150 MHz, DMSO*-d*_*6*_) *δ* 157.95, 153.51, 147.22, 143.41, 138.28, 133.26, 129.44, 126.51, 122.17, 121.64, 119.26, 116.40, 66.00, 62.42, 60.23, 54.05, 36.25, 26.43, 22.26, 14.56. ESI-HRMS calcd. for C_24_H_29_N_4_O_2_ [M + H]^+^ 405.2212, found: 405.2290.

##### 2-(4-((5-hydroxy-4,5-dihydrospiro[benzo[c]azepine-1,1’-cyclohexan]-2(3 H)-yl)methyl)-1 H-1,2,3-triazol-1-yl)benzonitrile (**11t**)

A brown solid, yield: 71.91%. Mp: 161.1-162.3℃. ^1^ H NMR (600 MHz, DMSO*-d*_*6*_) *δ* 8.10 (dd, *J* = 7.8, 1.0 Hz, 2 H), 7.96–7.91 (m, 4 H), 7.86 (d, *J* = 7.9 Hz, 1 H), 7.73 (t, *J* = 7.6 Hz, 2 H), 2.88 (s, 1 H), 2.72 (s, 2 H), 1.97 (s, 2 H), 1.63 (d, *J* = 11.6 Hz, 2 H), 1.47 (m, 4 H), 1.22 (m, 4 H); ^13^ C NMR (150 MHz, DMSO*-d*_*6*_) *δ* 162.75, 154.38, 147.59, 143.18, 138.48, 135.19, 135.11, 132.81, 130.41, 126.54, 125.99, 124.66, 119.80, 116.41, 107.29, 66.30, 62.47, 60.22, 56.18, 36.23, 31.21, 26.43, 14.53. ESI-HRMS calcd. for C_26_H_27_N_5_O [M + H]^+^ 424.2216, found: 414.2278.

##### 3-(4-((5-hydroxy-4,5-dihydrospiro[benzo[c]azepine-1,1’-cyclohexan]-2(3 H)-yl)methyl)-1 H-1,2,3-triazol-1-yl)benzonitrile (**11u**)

A brown solid, yield: 72.72%. Mp: 165.4-166.3℃. ^1^ H NMR (600 MHz, DMSO*-d*_*6*_) *δ* 7.95 (s, 1 H), 7.88 (d, *J* = 7.7 Hz, 3 H), 7.74 (t, *J* = 8.0 Hz, 3 H), 7.31 (d, *J* = 7.8 Hz, 2 H), 2.87 (s, 2 H), 2.72 (s, 2 H), 1.96 (s, 1 H), 1.63 (d, *J* = 11.1 Hz, 2 H), 1.46 (m, 3 H), 1.20 (m, 7 H); ^13^ C NMR (150 MHz, DMSO*-d*_*6*_) *δ* 162.68, 155.89, 148.03, 145.22, 143.17, 141.06, 137.61, 132.20, 131.54, 126.50, 124.62, 123.38, 121.88, 118.28, 113.20, 62.43, 60.19, 56.40, 52.75, 26.45, 22.28, 21.10, 14.45. ESI-HRMS calcd. for C_26_H_27_N_5_O [M + H]^+^ 424.2216, found: 414.2270.

##### 4-(4-((5-hydroxy-4,5-dihydrospiro[benzo[c]azepine-1,1’-cyclohexan]-2(3 H)-yl)methyl)-1 H-1,2,3-triazol-1-yl)benzonitrile (**11v**)

A brown solid, yield: 75.06%. Mp: 172.6-173.2℃. ^1^ H NMR (600 MHz, DMSO*-d*_*6*_) *δ* 8.15 (d, *J* = 8.7 Hz, 3 H), 8.07 (d, *J* = 8.8 Hz, 3 H), 7.36–7.32 (m, 3 H), 7.21 (s, 3 H), 2.89 (s, 1 H), 2.72 (d, *J* = 12.7 Hz, 2 H), 1.98 (s, 2 H), 1.64 (d, *J* = 11.7 Hz, 2 H), 1.47 (m, 2 H), 1.21 m, 8 H); ^13^ C NMR (150 MHz, DMSO*-d*_*6*_) *δ* 153.33, 147.12, 142.66, 139.06, 136.15, 133.63, 129.94, 125.51, 121.00, 119.61, 117.60, 114.07, 110.09, 61.42, 59.18, 54.06, 47.85, 30.15, 25.38, 21.22, 13.50. ESI-HRMS calcd. for C_26_H_27_N_5_O [M + H]^+^ 424.2216, found: 414.2289.

### Cell lines and cell culture

A549, OVCAR-3, HCT-116, and MCF-7 were purchased from American type culture collection (ATCC, USA). All cell lines were cultured in RPMI-1640 (Beijing Thermo Fisher Scientific Company, China), supplemented with 10% FBS at 37 °C and 5% CO_2_. And all cell lines used in the experiment were tested for mycoplasma contamination every two weeks.

### MTT assay

A549, OVCAR-3, HCT-116, and MCF-7 cells were seeded in 96-well plates, and then treated with target compounds for 72 h. Then, 20 µL MTT (5 mg/mL, in PBS) was added into each well, and dissolved in 150 µL DMSO after incubation for 4 h. Finally, the absorbance was measured with microplate reader (490 nm).

### Enzyme inhibitory activity assay

The PARP-1 and PARP-2 inhibition assays were performed by the colorimetric 96-well PARP assay kits provided by BPS Bioscience, USA. (Catalog No. 80,580, 80,581)

### Molecular docking and molecular dynamics study

Molecular docking studies of compound **11b** were carried out as previously reported [31, 32]. The three-dimensional structure of PARP-1 was retrieved from the Protein Data Bank (PDB code: 4BJC).

In the docking results, the binding conformations from the molecular docking results were extracted, and preserved for further molecular dynamics (MD) simulation. We then analyzed the binding affinity and stability in Groningen Machine for Chemicals Simulations (GROMACS) and VMD (visual molecular dynamics, version 1.9.3) [36].

### Apoptosis analysis

A549 cells were cultured in 6-well plates (3.0 × 10^5^/well), and treated with DMSO (1%) or compound **11b** for 48 h. The cells were then collected, washed with PBS, and stained with FITC-Annexin-V and PI (Promega Corporation, USA). Finally, the apoptosis of A549 cells was detected by flow cytometry (Becton Dickinson and Company, USA; FACS Calibur).

### Western blotting

The cells were plated in 6-well plates and incubated with different concentrations of compound **11b** for a specified time. The cells were then collected, and tested using a standard western blot as described before. [26, 31, 32]

### Prediction of ADMET properties

The properties of compound **11b** were predicted and analyzed using the admetSAR and SwissADME prediction website (http://lmmd.ecust.edu.cn/admetsar2/, and http://www.swissadme.ch/) [36].

### Statistical analysis

All results were presented as the means ± SD. The statistical significance of differences was determined using Student’s t-test, and one-way analysis of variance (ANOVA) was used. And, Tukey’s in post hoc analysis was applied. Data were analyzed with Prism 9.0 (Graph Pad Software, San Diego, CA, USA). P < 0.05 were considered statistically significant.

### Electronic supplementary material

Below is the link to the electronic supplementary material.

Supplementary Material 1

## Data Availability

The datasets used and/or analyzed during the current study are available from the corresponding author on reasonable request.
