# Supplementary material for: Discovery of novel 2,3,4,5-tetrahydrospiro[benzo[c]azepine-1,1’-cyclohexan]-5-ol derivatives as PARP-1 inhibitors
Source: BMC Chem. 2023 Oct 27;17(1):147. doi: 10.1186/s13065-023-01060-8 (PMC10612255; doi:10.1186/s13065-023-01060-8)
Supplement: Supplementary file 1 — Supplementary Material 1 [file 13065_2023_1060_MOESM1_ESM.docx]

**Supplementary Data**

**Discovery of novel 2,3,4,5-tetrahydrospiro[benzo[*c*]azepine-1,1'-cyclohexan]-5-ol derivatives as PARP-1 inhibitors**

Ling Yu^1^, Jian-hui Li^2^, Ju Zhu^3^, You-de Wang^4^, Zhi-wei Yan^4^, Li-ying Zhang^4^ and Shuai Li^4,^*

**^1^** Department of Pharmacy, Anorectal Hospital of Chengde Medical University, Chengde 067000, P. R. China

**^2^** Department of Preventive Medicine, Chengde Medical University, Chengde 067000, P. R. China

**^3^** School of Pharmacy, China Medical University, 77 Puhe Road, North New Area, Shenyang 110122, China

**^4^** Key Laboratory of Traditional Chinese Medicine Research and Development of Hebei Province, Hebei Key Laboratory of Nerve Injury and Repair, Institute of Traditional Chinese Medicine, Chengde Medical University, Chengde 067000, P. R. China

**^*^** Corresponding author: Dr. Shuai Li, Chengde Medical University, Anyuan Road, Chengde 067000, P. R. China

E-mail: [cmuyhls@163.com](mailto:cmuyhls@163.com)

**1. Graphical abstract**

**
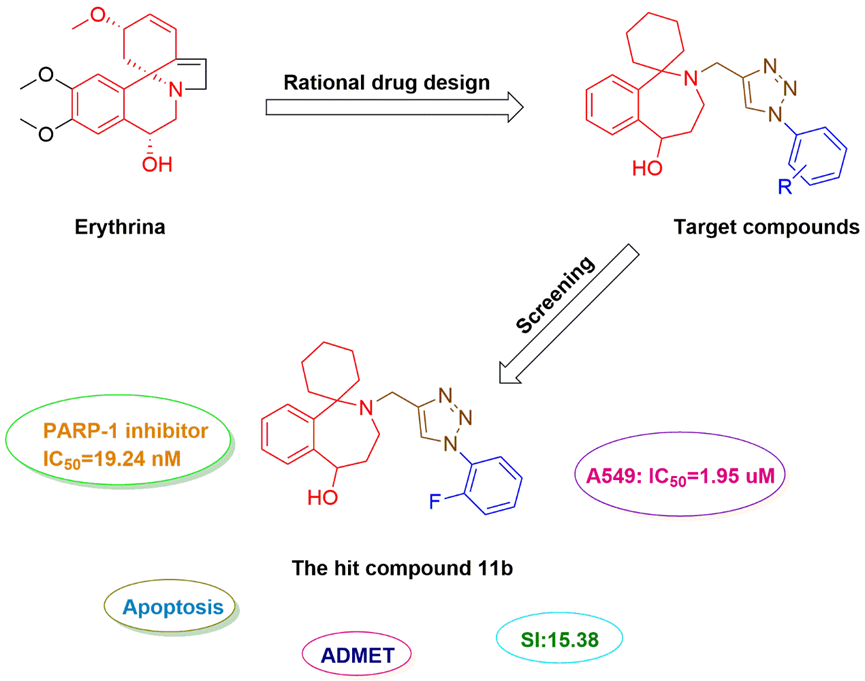
**

**Fig. S1. The Graphical abstract of this study**.

**2. Synthesis section**

*The synthesis of 1-phenylcyclohexan-1-ol.*

Mg (8.13 g, 0.33 mol), catalyst amount of I_2_, and compound **1** (10.00 g, 0.064 mol) were added to the diethyl ether at room temperature, and the mixture was heated to 35℃. Then, the solution of compound **1** (40.00 g, 0.25 mol) in 50 mL of diethyl ether was dropped into the above mixture over 0.5 h, and then cyclohexanone (34.38 g, 0.35 mol) was added to the reaction system. The mixture was stirred at room temperature for 3 h. This series of experimental operations needed to be carried out under anhydrous and anaerobic conditions. After the reaction was completed, the mixture was quenched with saturated aqueous ammonium chloride (100 mL). Next, the mixture was extracted with Et2O (3 × 100 mL), and the combined extracts were dried (MgSO_4_). Evaporation of the solvent to give crude compound **2** as yellow oil. The crude product was purified by column chromatography. Yield: 95.50%.

*The synthesis of 1-phenylcyclohexan-1-amine.*

Sodium azide (8.11 g, 0.12 mol) and trifluoroacetic acid (25.88 g, 0.23 mol) were added to the solution of compound **2** (20.00 g, 0.11 mol) in DCM, and this step was carried out at -5℃. The reaction was stirred at room temperature for 12 h. Evaporation the solvent and dissolved it with tetrahydrofuran. Note that a large amount of saturated anhydrous sodium carbonate solution should be added before the solvent was evaporated to destroy the formed hydrazoic acid and perform the liquid separation. The mixture was then added to lithium aluminum hydride (4.77 g, 0.13 mol) dissolved in tetrahydrofuran at -5℃. The reaction was stirred at 40℃ for 12 h. After the reaction was completed, water was added dropwise to the reaction system at -5℃ to achieve the purpose of quenching lithium aluminum hydride. The precipitate was then suction filtered, and the solvent was evaporated in vacuo. Next, the pH of the mixture was adjusted to 2-3. And then the mixture was extracted with Et2O (3 × 100 mL) to remove impurities. After that, the pH of the aqueous layer should be adjusted to 9-10. The mixture was extracted with DCM (3 × 100 mL), and the combined extracts were dried (MgSO_4_). Evaporation of the solvent to give compound **4** as white oil. Yield: 89.43%.

*The synthesis of methyl 3-((1-phenylcyclohexyl) amino) propanoate.*

The compound **4** (20.00 g, 0.11 mol), DBU (8.69 g, 0.057 mol) and methyl acrylate (10.81 g, 0.13 mol) were added to a 250 mL reaction flask at 40℃ for 12 h to give crude compound **5** as white oil. The crude product was purified by column chromatography. Yield: 79.5%. Note that no solvent was needed for this experimental procedure.

*The synthesis of 3-((1-phenylcyclohexyl)amino)propanoic acid.*

To a solution of compound **5** (15.00 g, 0.057 mol) in water, sodium hydroxide (4.59 g, 0.115 mol) was added, and the reaction was stirred at 50℃ for 2 h. As the reaction progresses, compound **5** gradually dissolved in the form of sodium salt in water. Then we adjusted the pH of the solution to 6 and evaporated the solvent to give compound **6** as white oil, which was used directly in the next step. Yield: 97.15%.

*The synthesis of 3,4-dihydrospiro[benzo[c]azepine-1,1'-cyclohexan]-5(2H)-one.*

To a solution of compound **6** (20.00 g, 0.081 mol) in DCM, thionyl chloride (19.24 g, 0.162 mol) was added, and the reaction was stirred at 35℃ for 2 h. Evaporation of the solvent and dissolved it with DCM. Then we added anhydrous aluminum trichloride (12.94 g, 0.097 mol) to the reaction system. The reaction was stirred at 25℃ for 24 h to give crude compound **8** as white oil. During the post-treatment, the reaction solution was slowly poured into ice water and continuously stirred, and then liquid separation was carried out. Finally, the organic layer was alkalized for extraction. The crude product was purified by column chromatography. Yield: 35.2%.

*The synthesis of 2-(prop-2-yn-1-yl)-3,4-dihydrospiro[benzo[c]azepine-1,1'-cyclohexan]-5(2H)-one.*

To a solution of compound **8** (1.00 g, 0.004 mol) in acetonitrile, 3-bromoprop-1-yne (0.57 g, 0.005 mol) was added, and the reaction was stirred at 70℃ for 12 h to give crude compound **9** as yellow powder. This step required the addition of an equal amount of K_2_CO_3_ as an acid-binding agent. The crude product was purified by column chromatography. Yield: 89.96%.

*General procedure for the synthesis of compounds* ***11a-11v***

Hydrogen chloride (2.68 g, 0.028 mol), sodium nitrite (1.63 g, 0.024 mol) and sodium azide (1.54 g, 0.024 mol) were sequentially added to the corresponding aqueous solution of aniline (2.00 g, 0.021 mol) every 0.5 h, and the reaction was stirred at 0℃ for 5 h to give the corresponding azidobenzene. Then, to a solution of compound **10** (0.50 g, 0.004 mol) in DMF, corresponding azidobenzene, anhydrous copper sulfate (0.20 g), and Vitamin C (0.20 g) were added. The reaction was placed in a microwave reactor and kept stirring at 35℃ for 9 minutes to give crude compounds **11a-11v.** The crude product was purified by column chromatography.

**3. Western blot analysis section**

Since we conducted several Western blot analysis experiments and select the best results to present in the manuscript, the gel and the blotting were cropped in this experiment. However, the above procedures did not affect our experimental results, and we demonstrated the results of uncropped gel and blotting **(Fig. S2-S5)**. All data are true and valid.


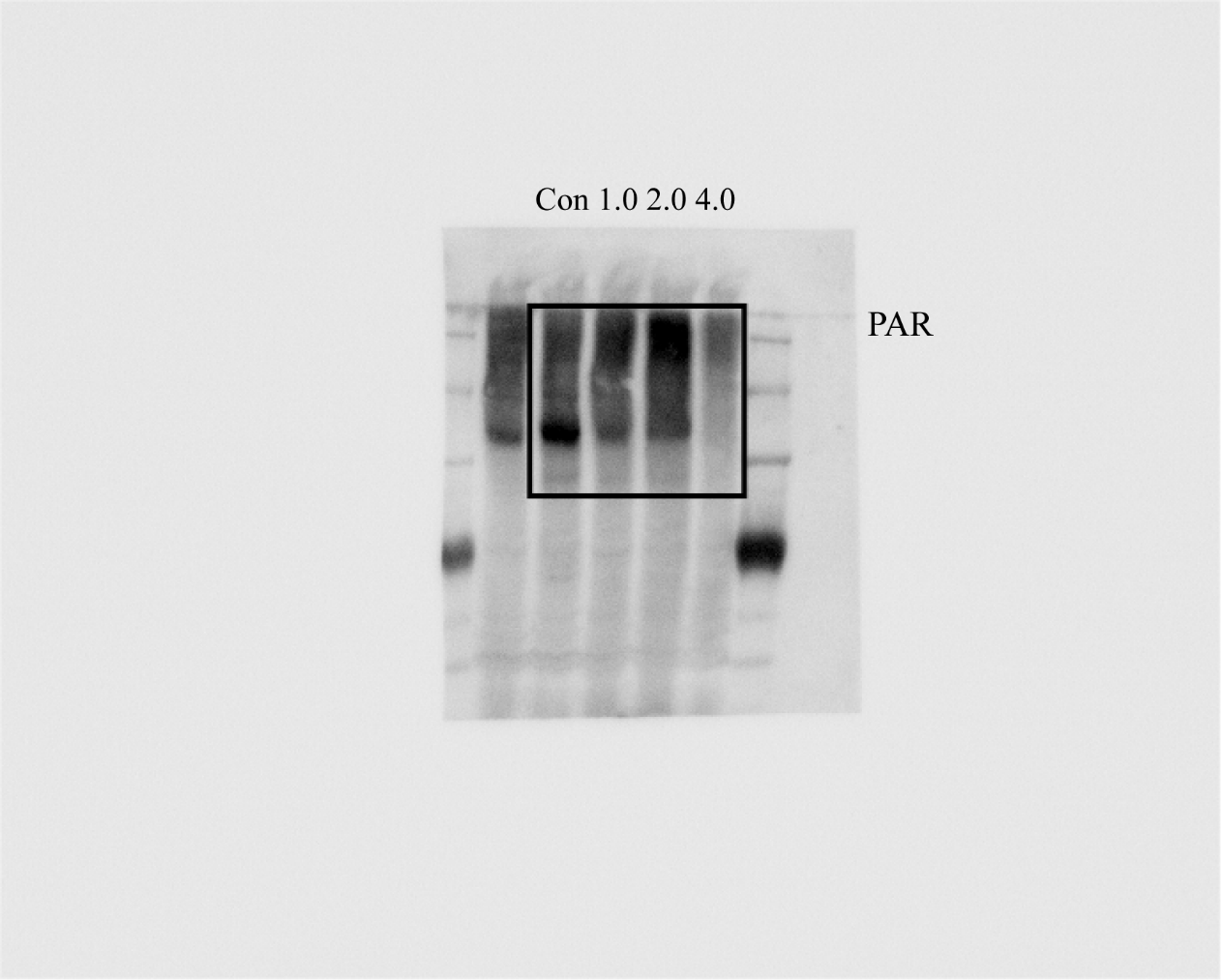


**Fig. S2.** Uncropped data from the PAR analysis experiment.


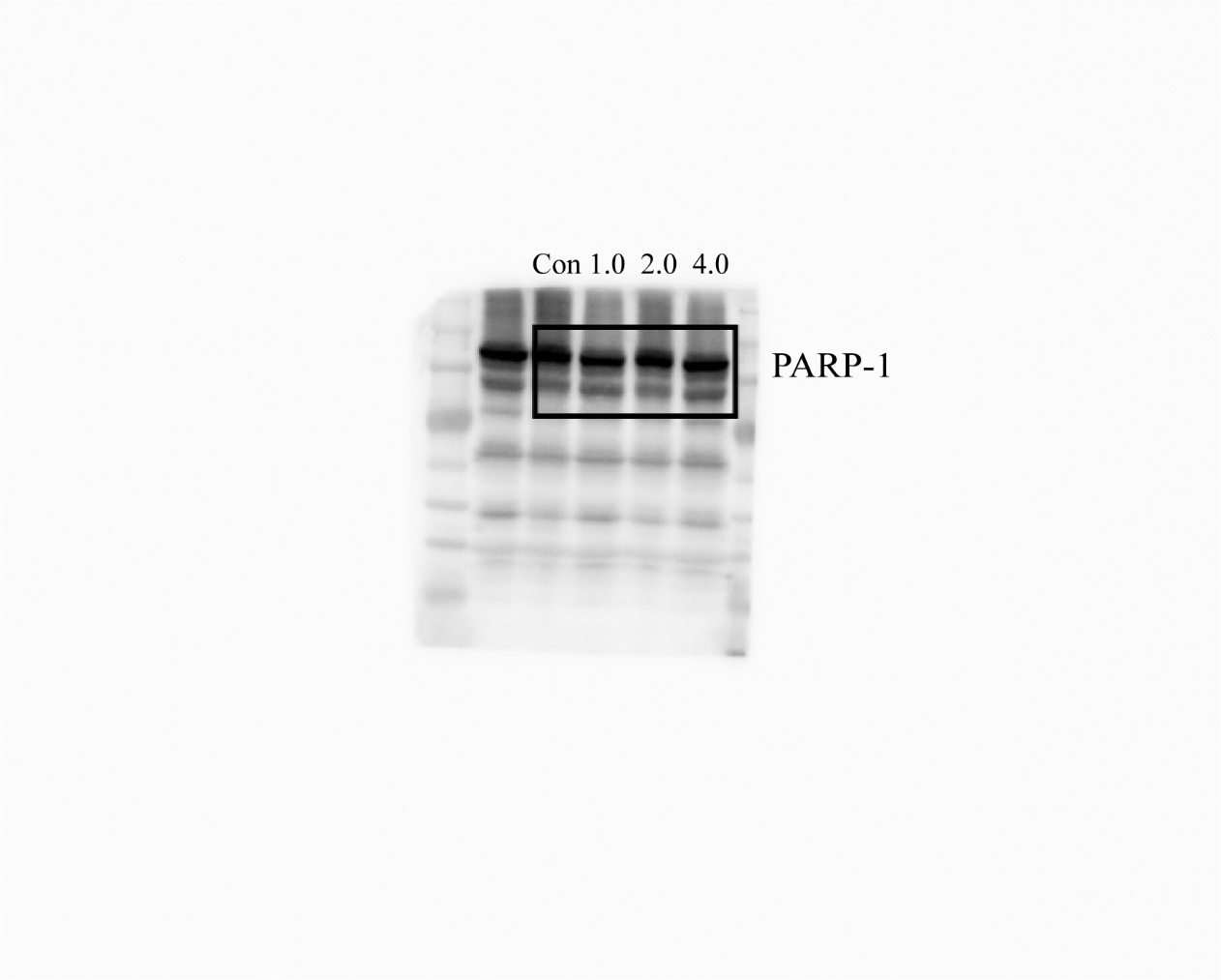


**Fig. S3.** Uncropped data from the PARP-1 analysis experiment.


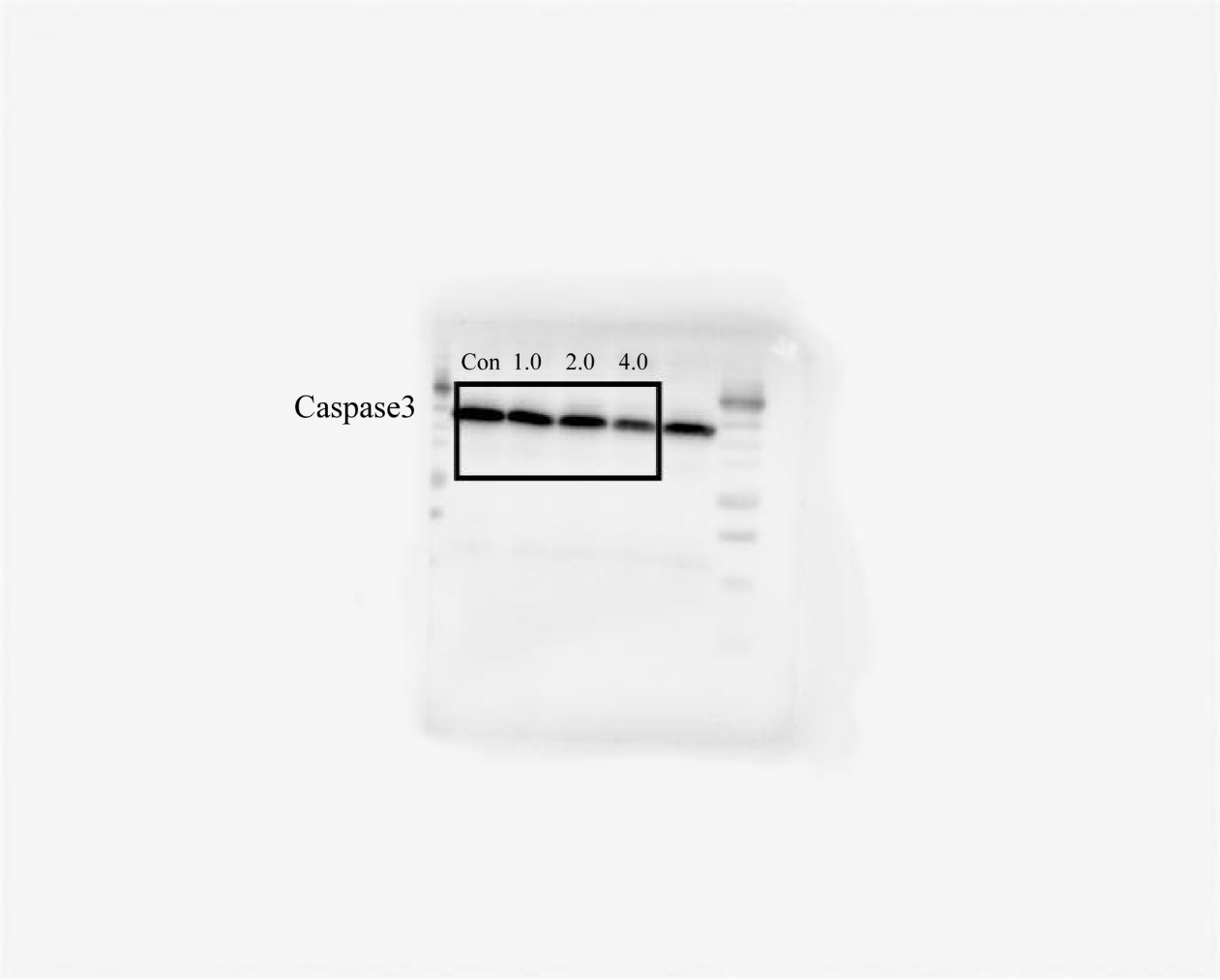


**Fig. S4.** Uncropped data from the Caspase3 analysis experiment.


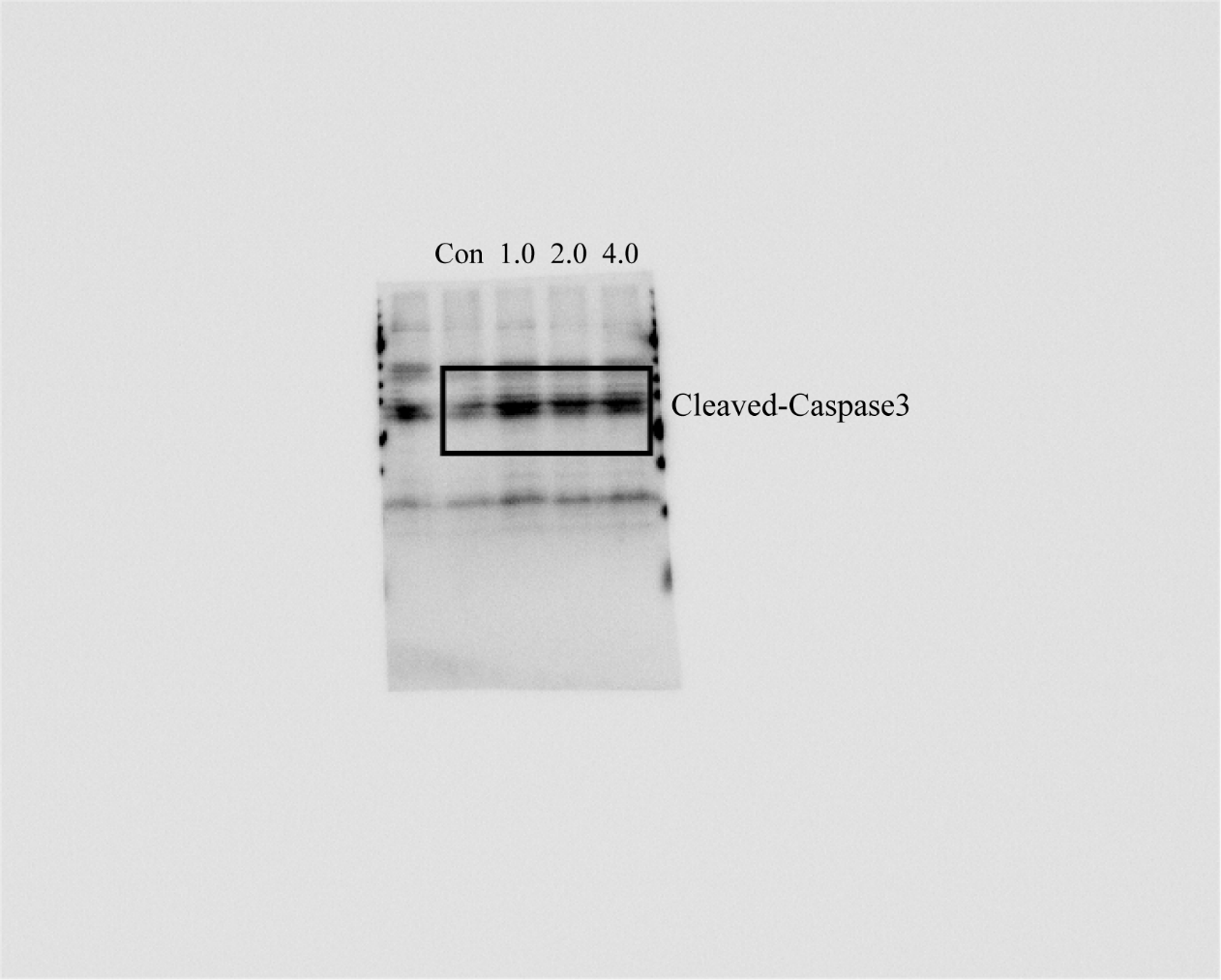


**Fig. S5.** Uncropped data from the Cleaved-Caspase3 analysis experiment.

**4. The results of ^1^H NMR, ^13^C NMR and HRMS spectra of the target compounds**

**11a**


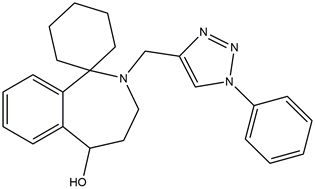


**
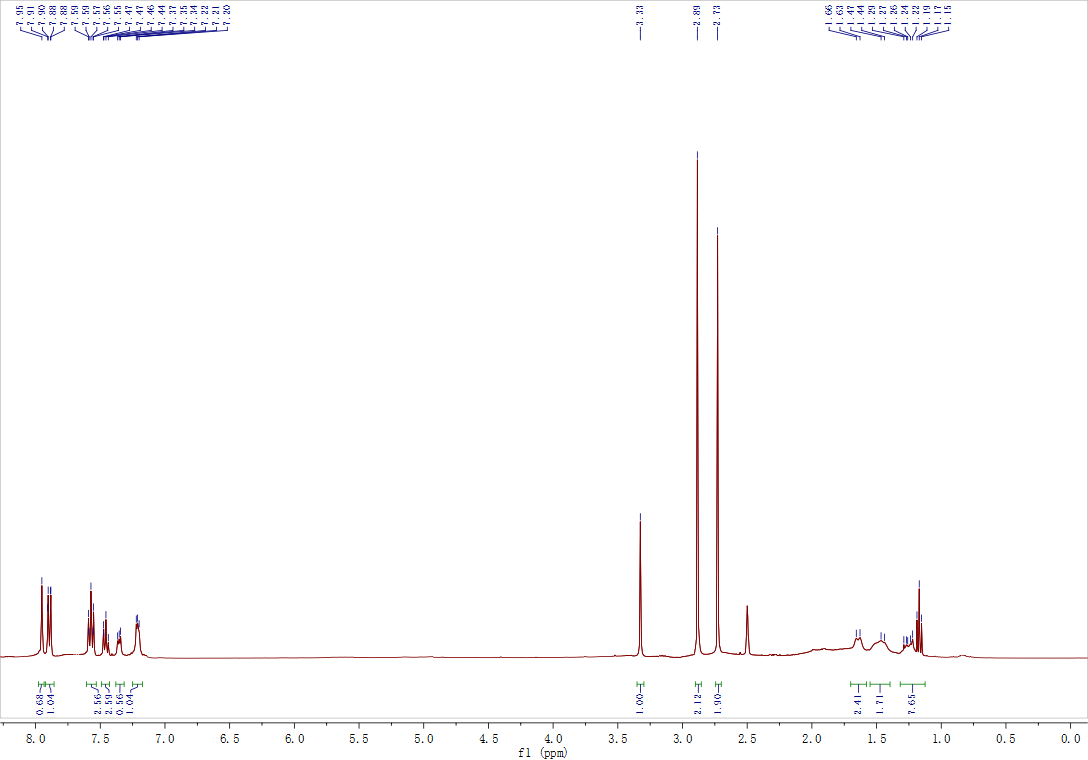
**

**
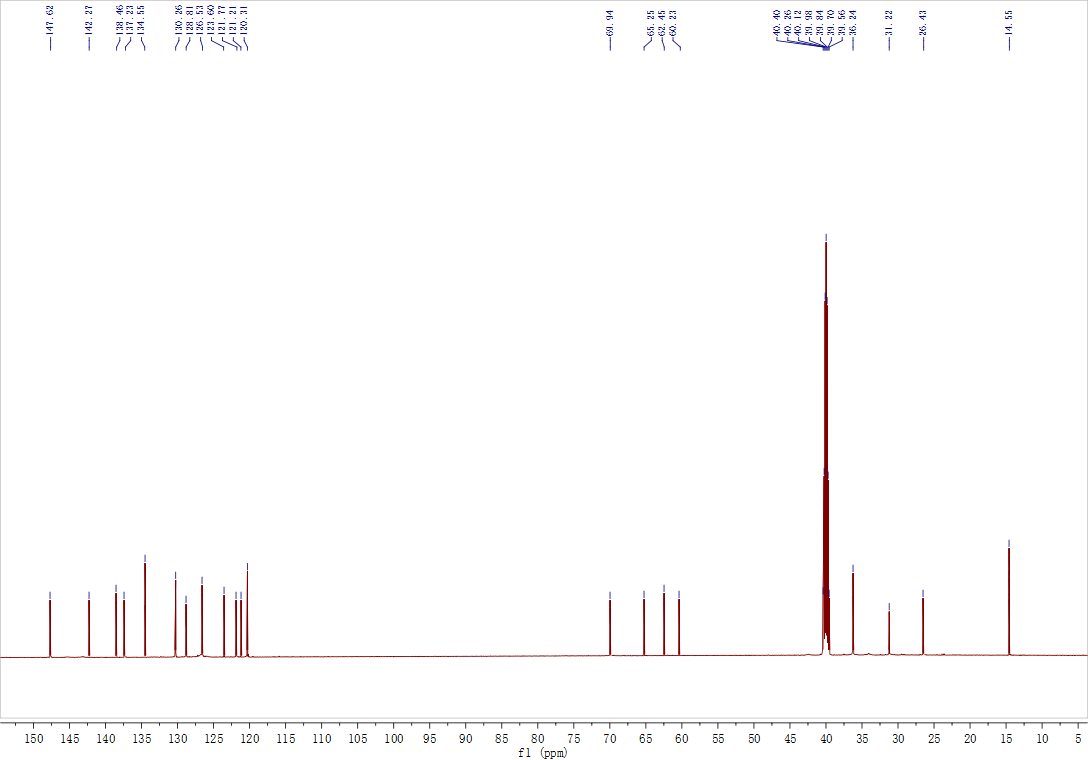
**

**
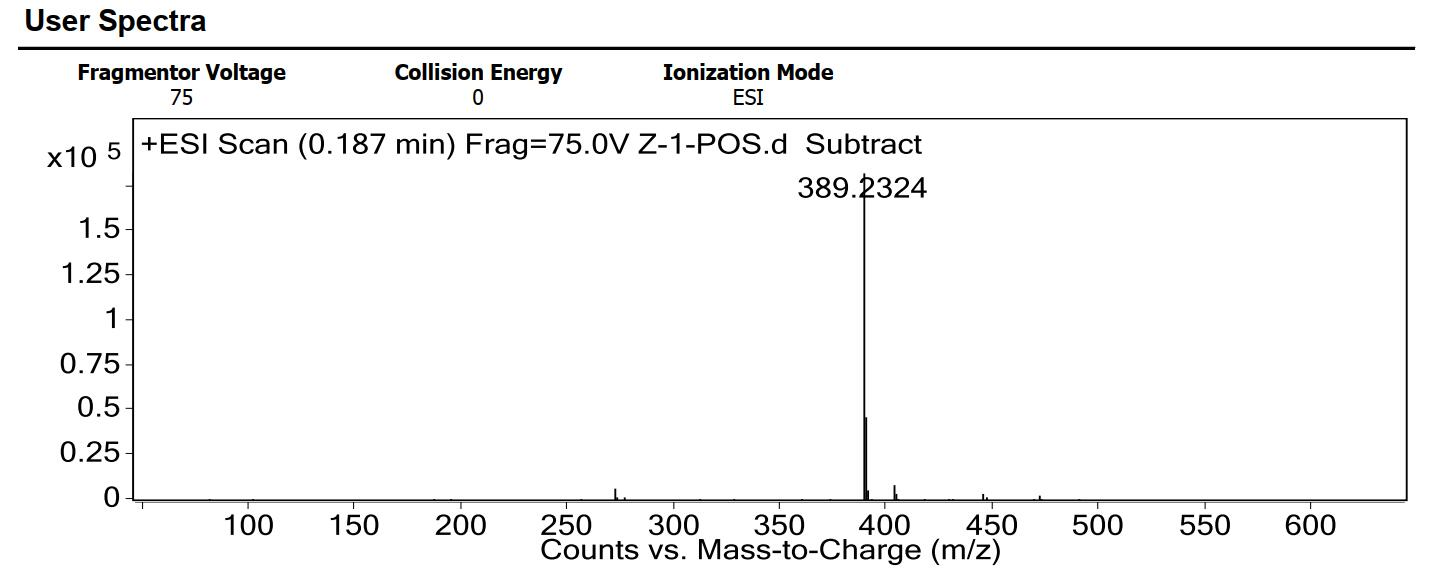
**

**11b**

**
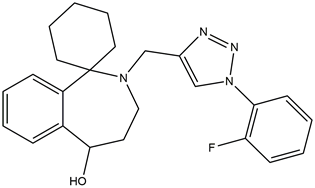
**

**
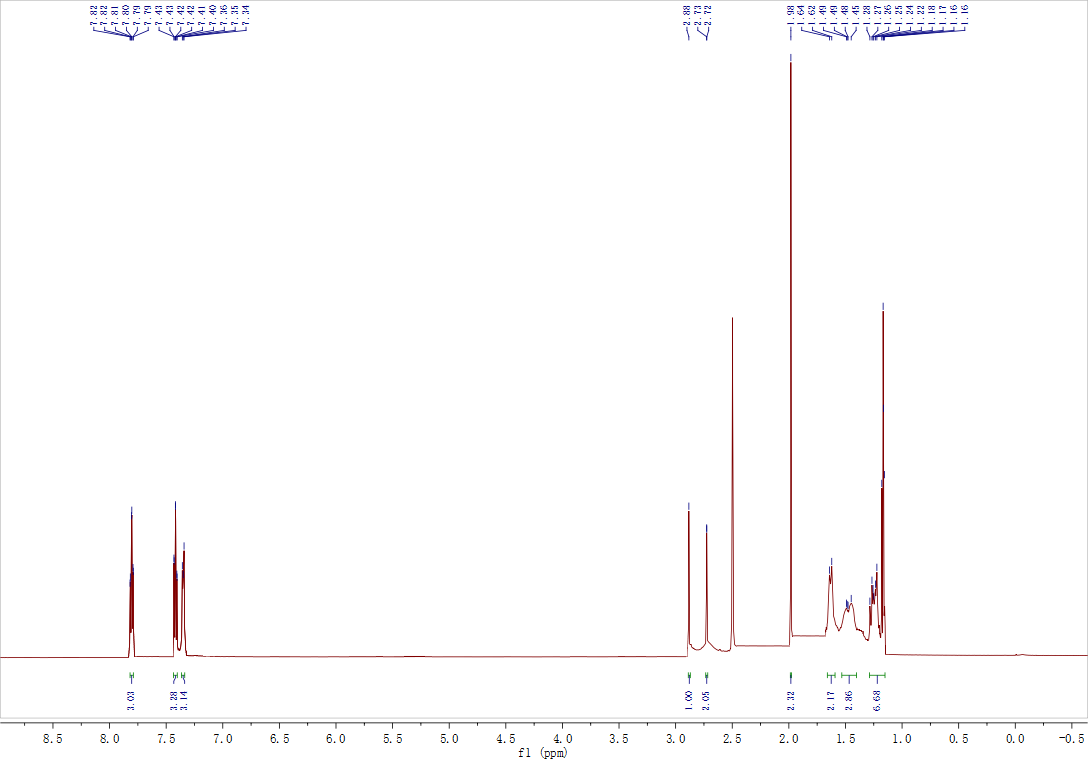
**

**
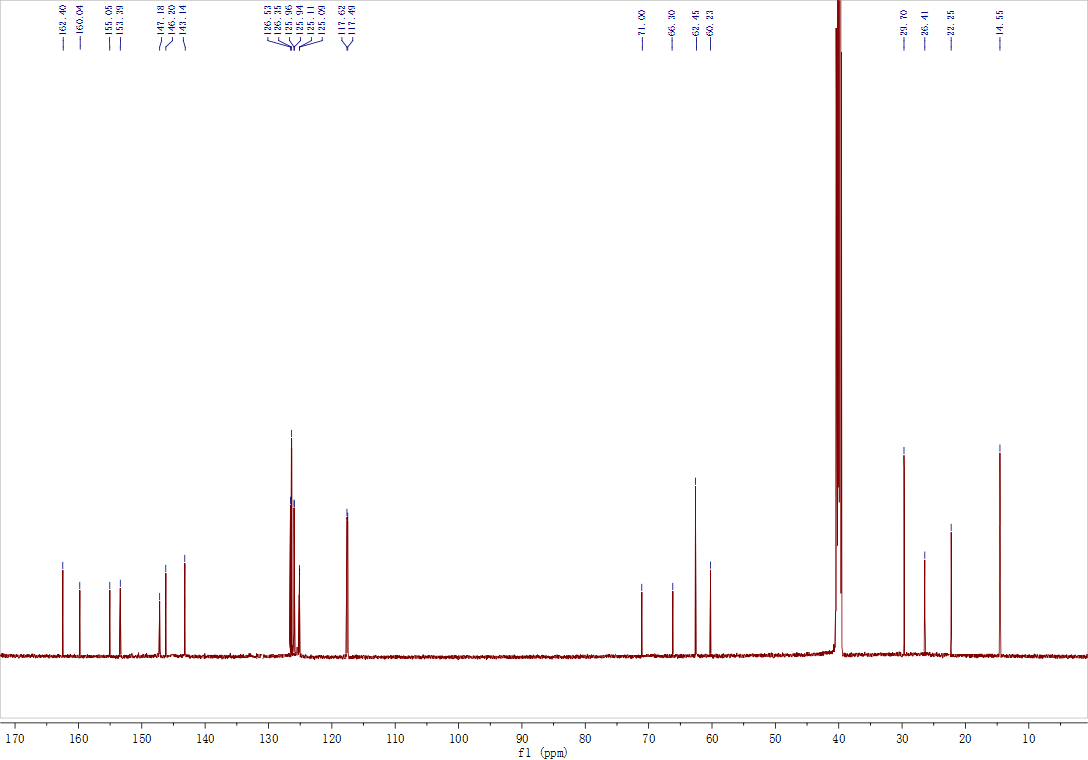
**

**
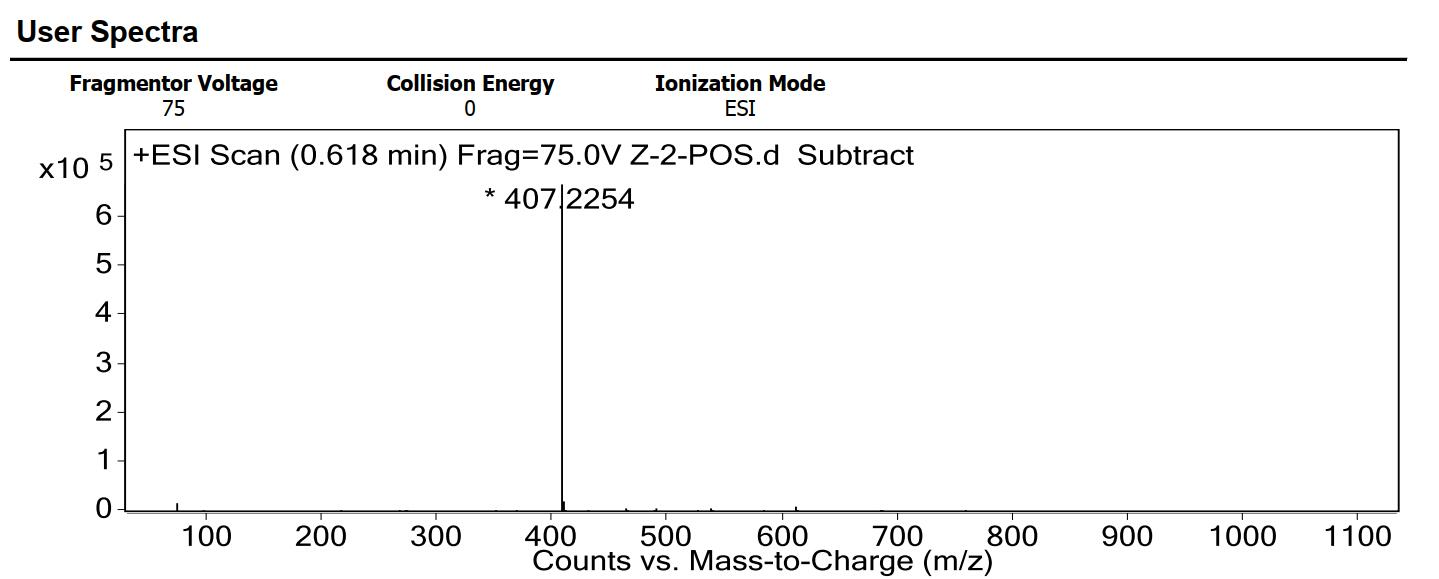
**

**11c**

**
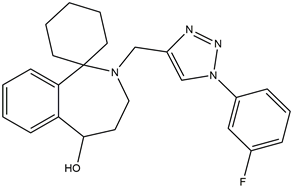
**

**
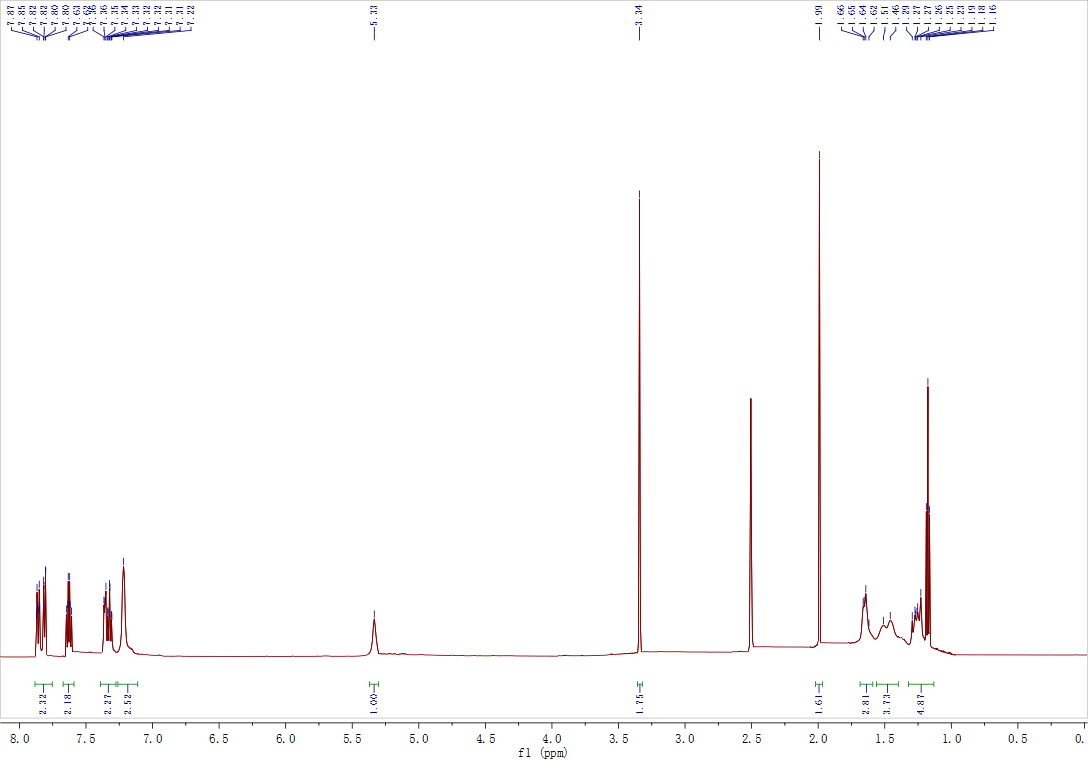
**

**
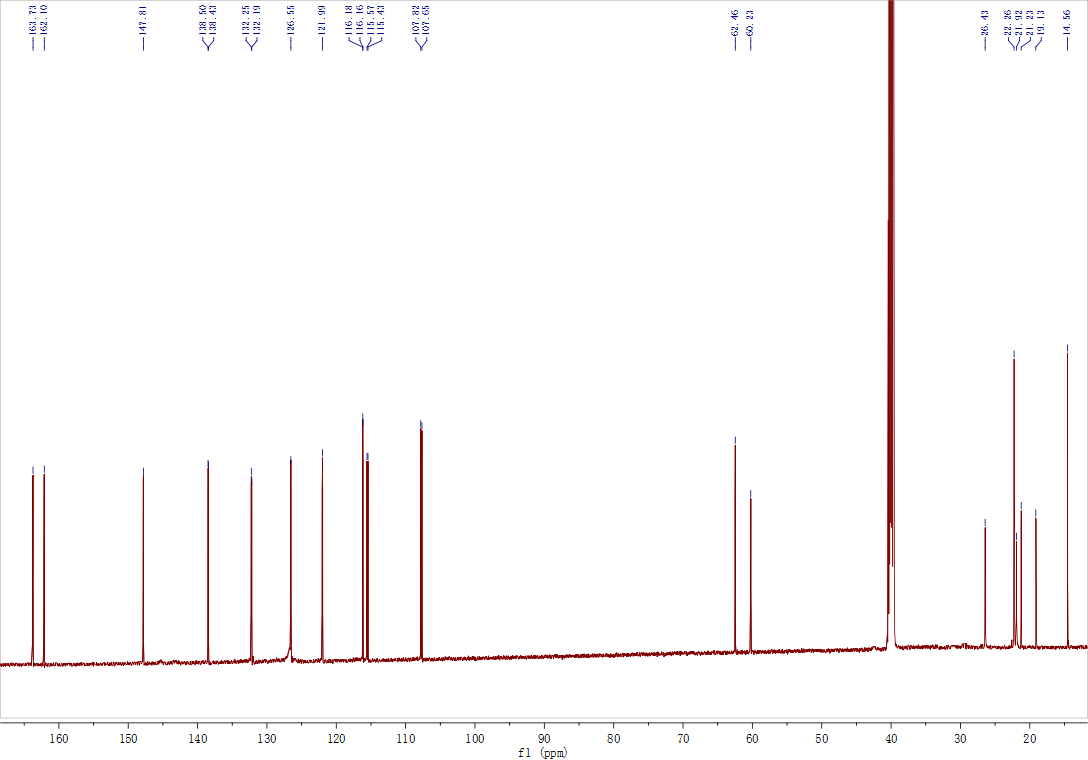
**

**
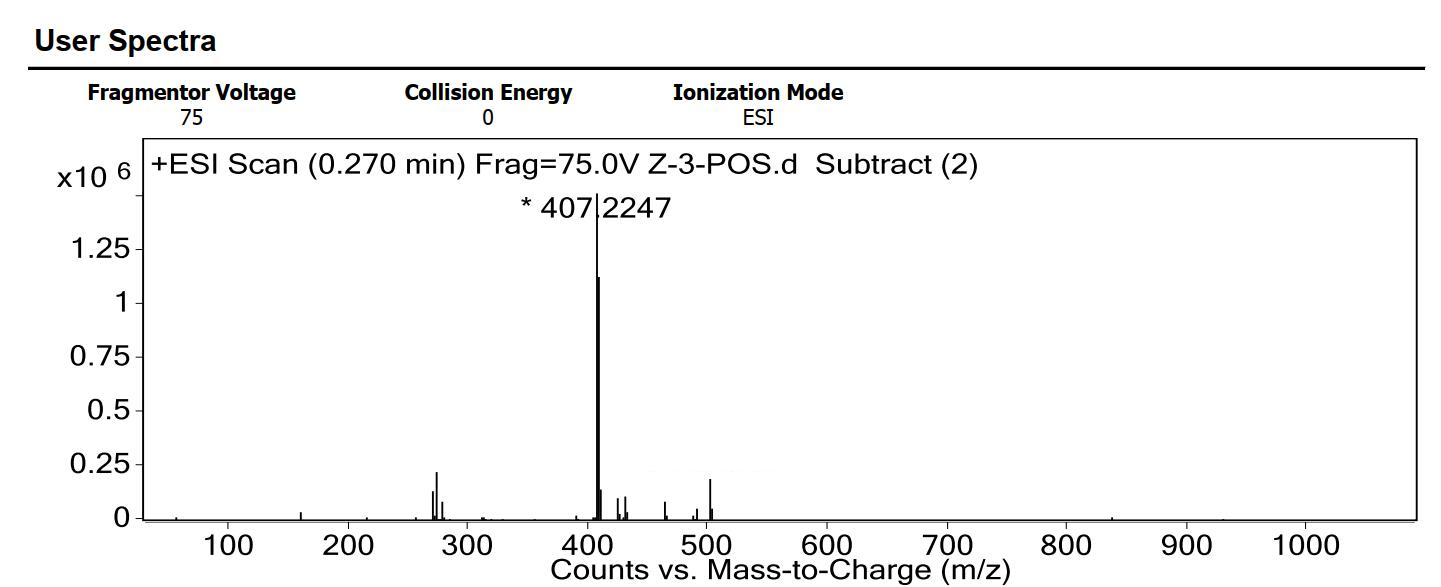
**

**11d**

**
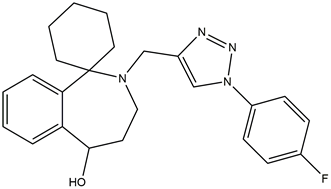
**

**
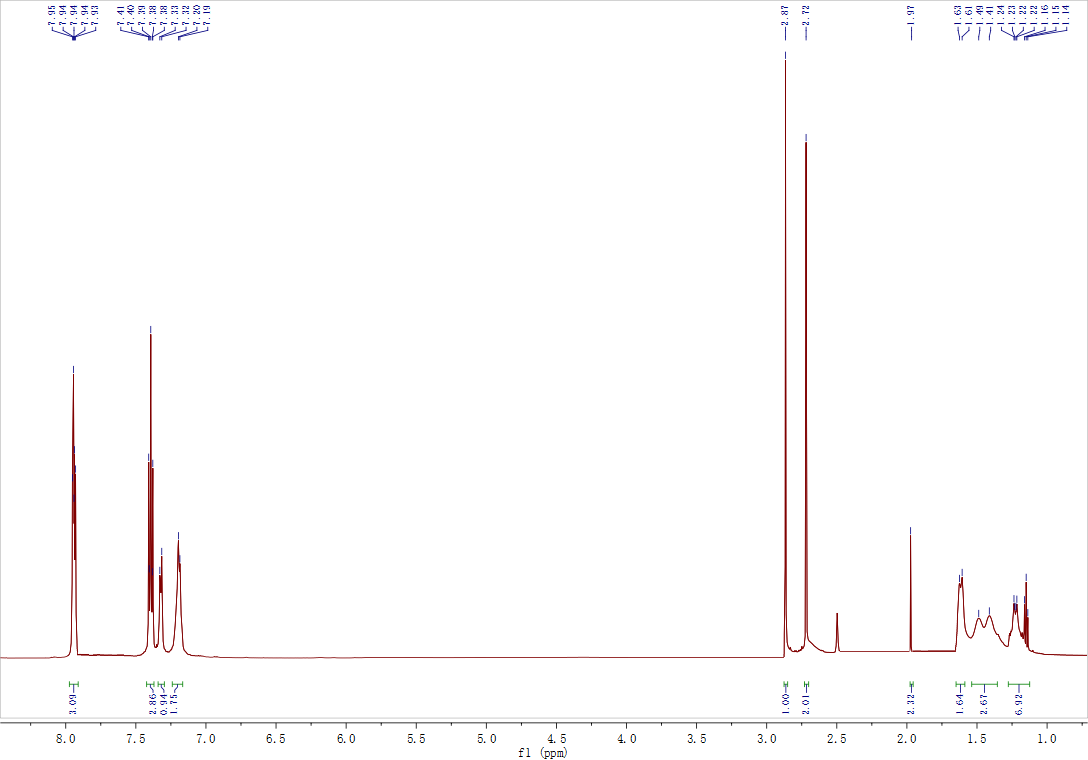
**

**
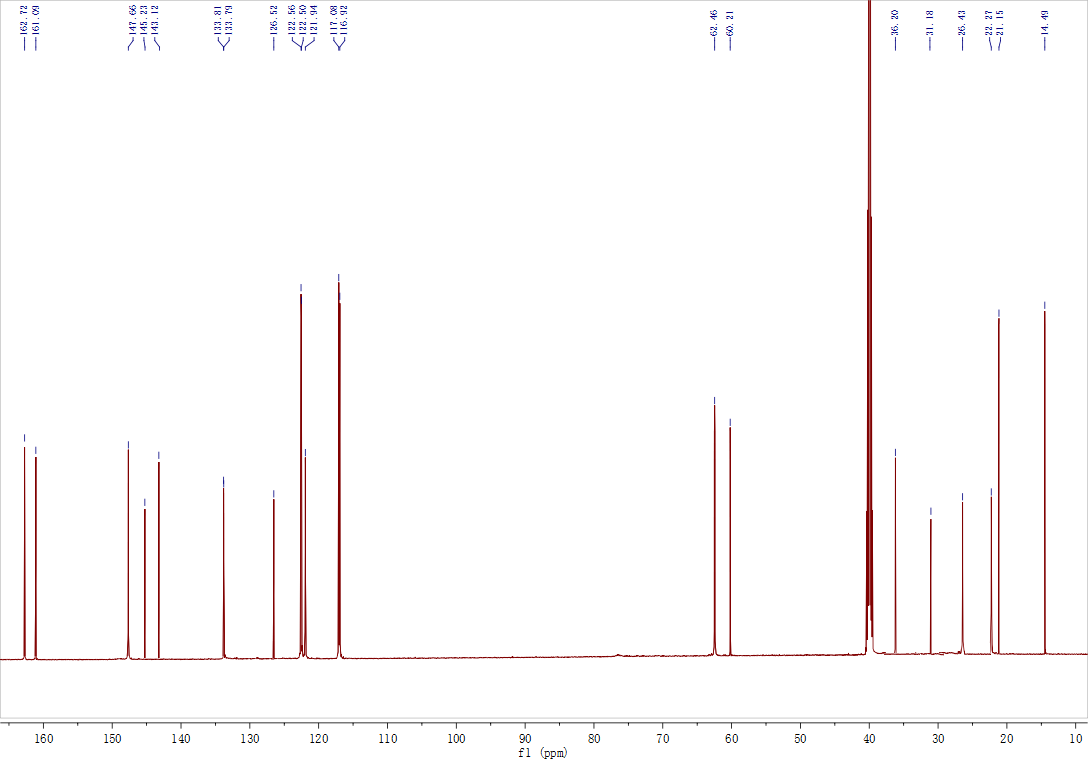
**

**
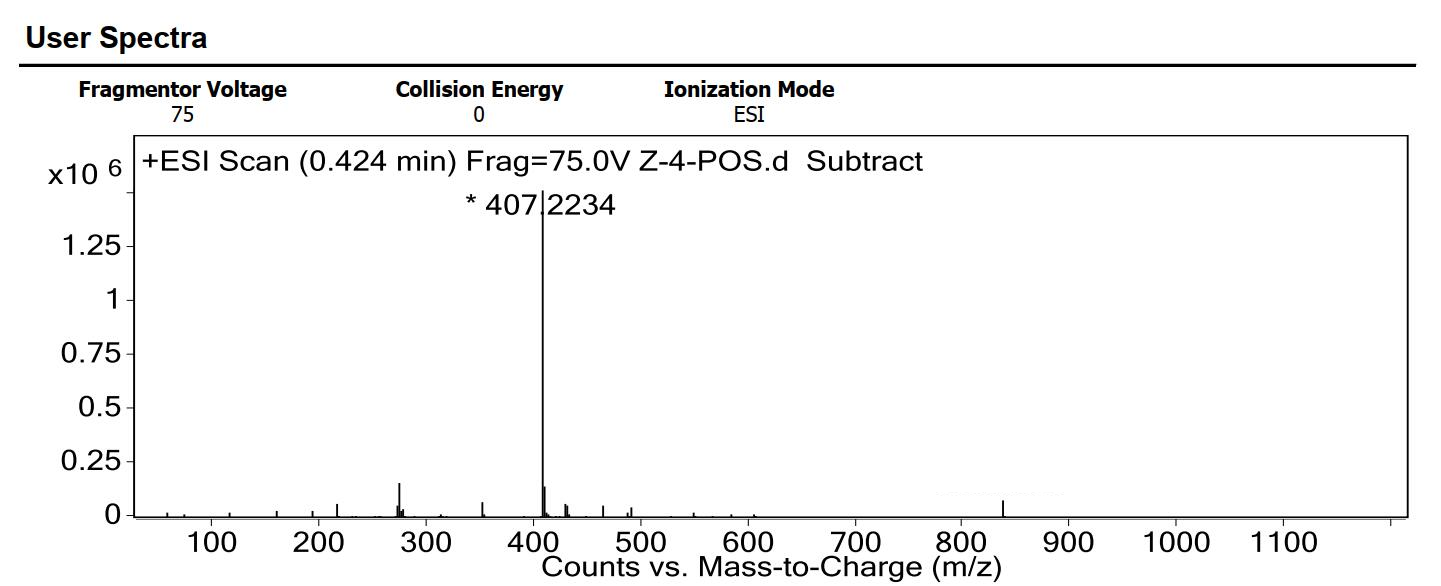
**

**11e**

**
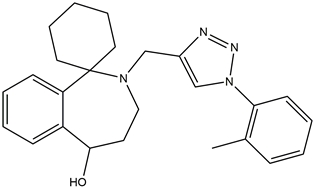
**

**
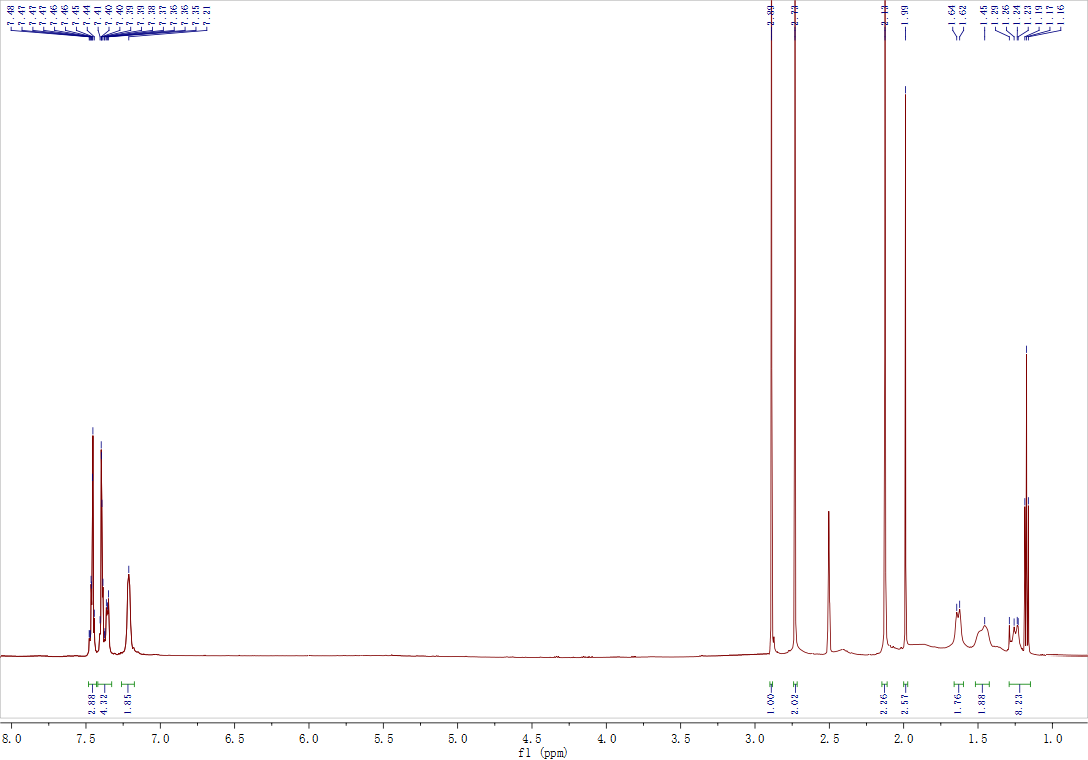
**

**
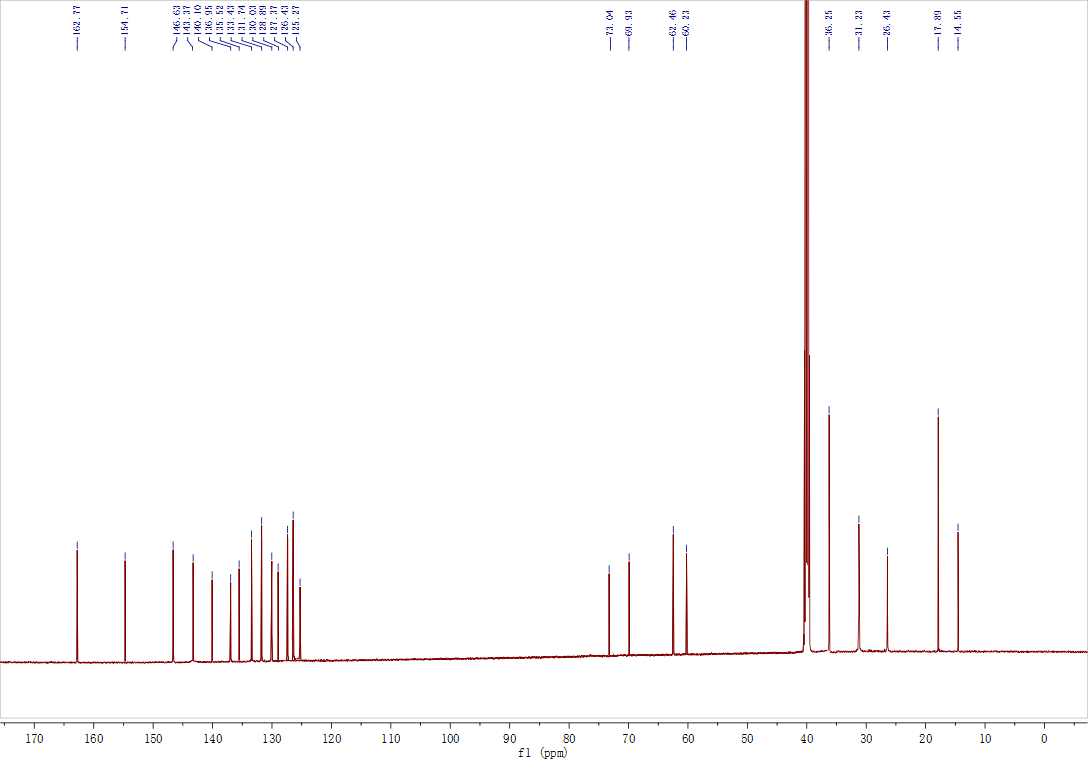
**

**
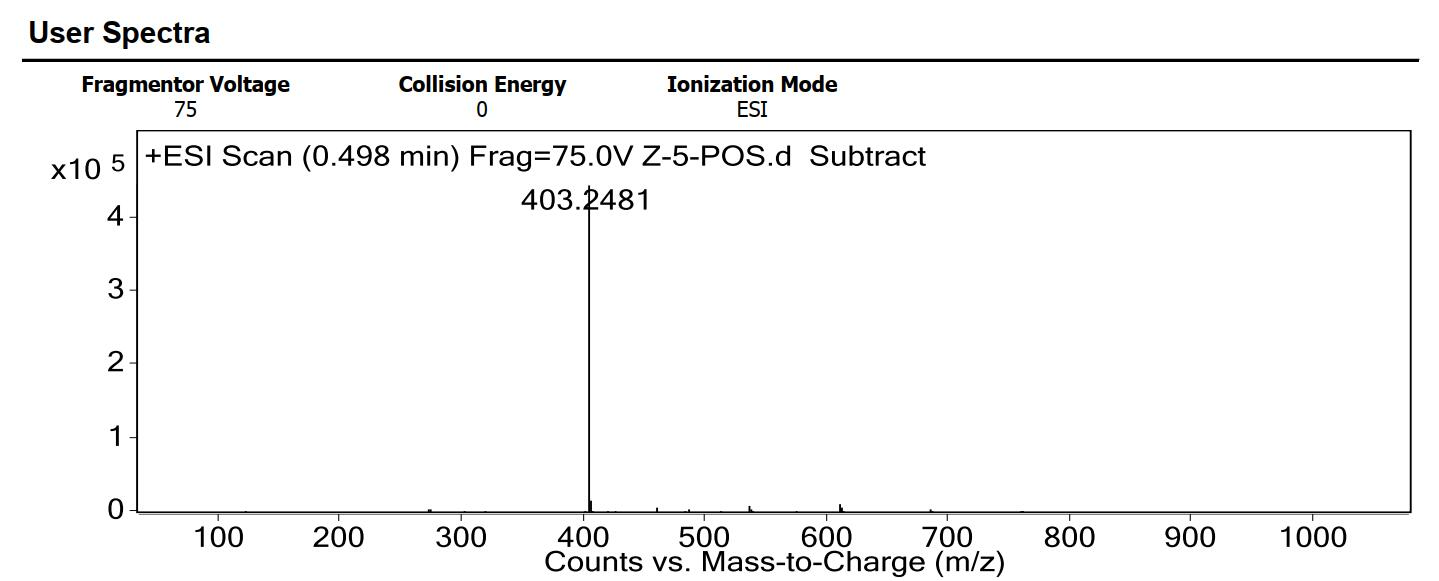
**

**11f**

**
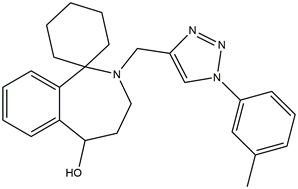
**

**
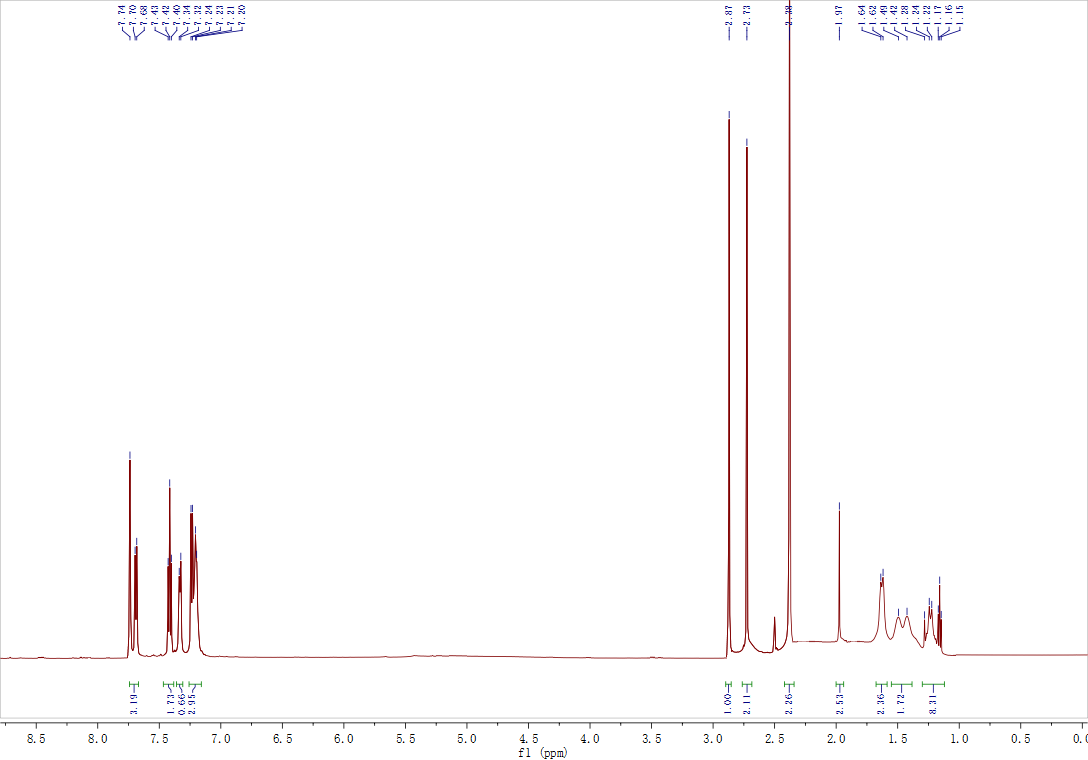
**

**
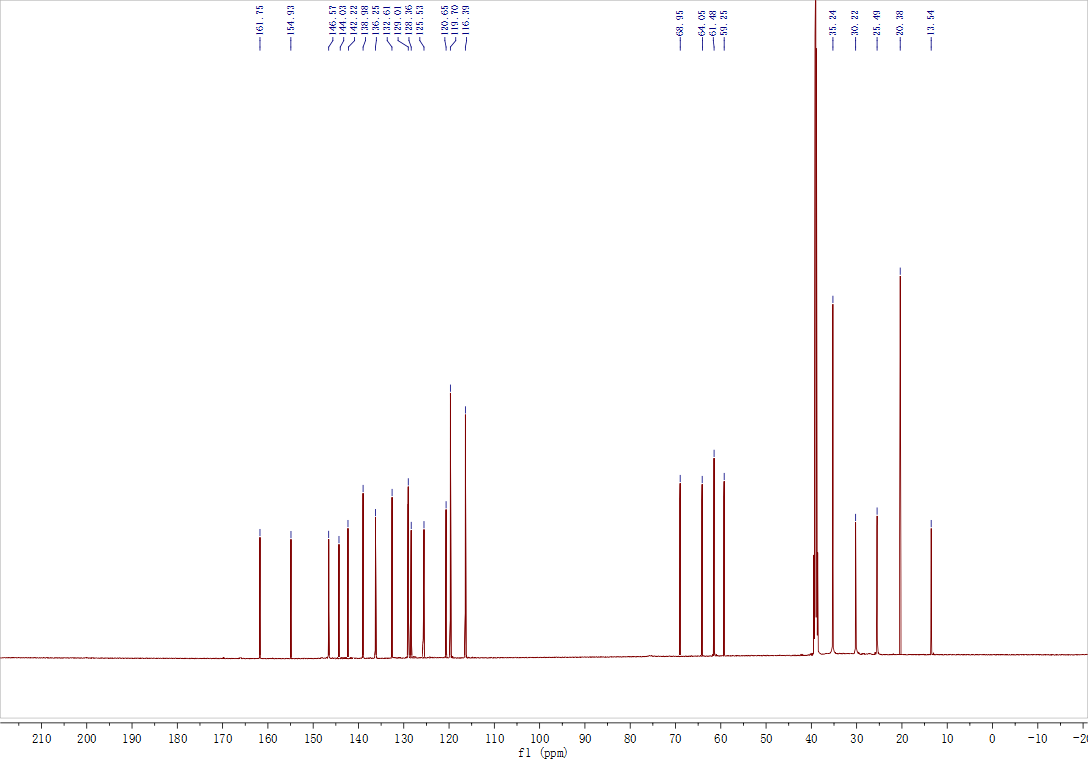
**


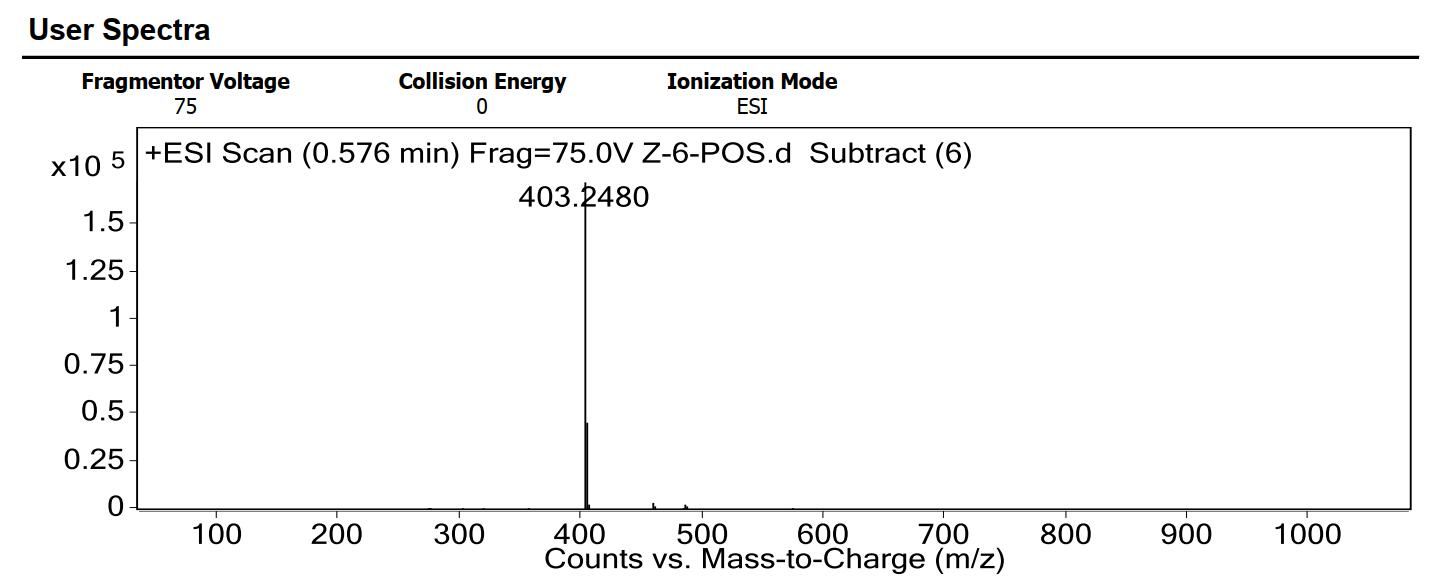


**11g**


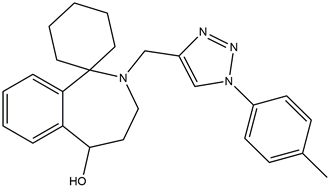


**
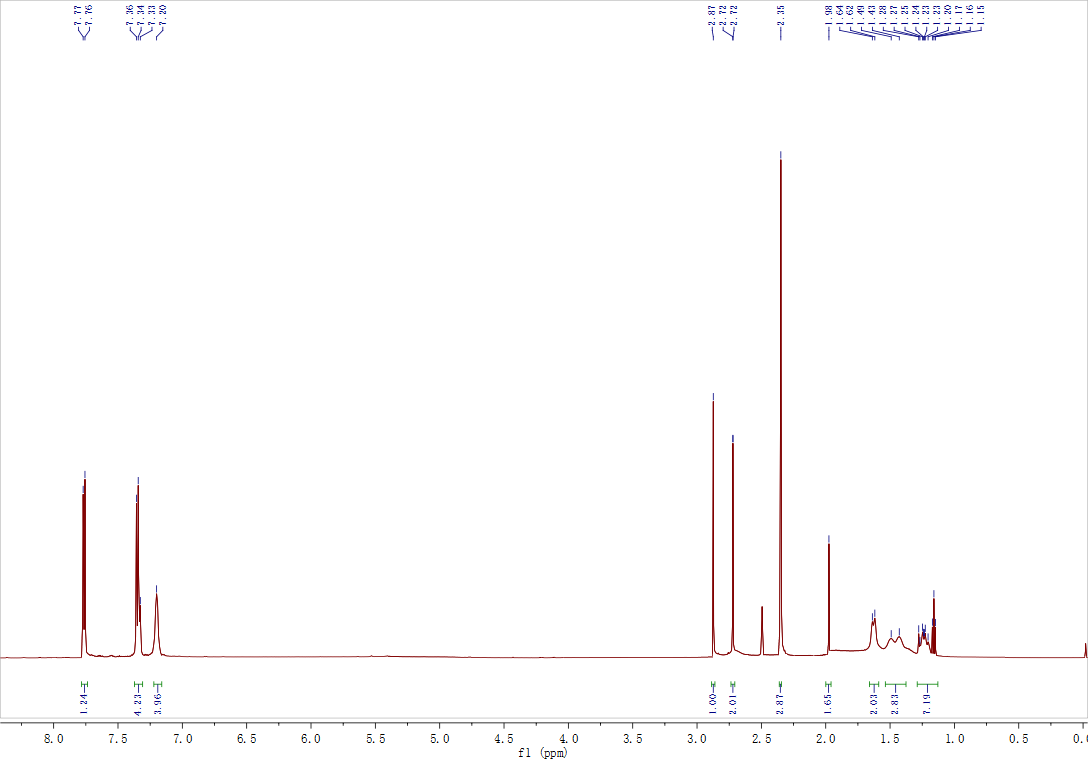
**

**
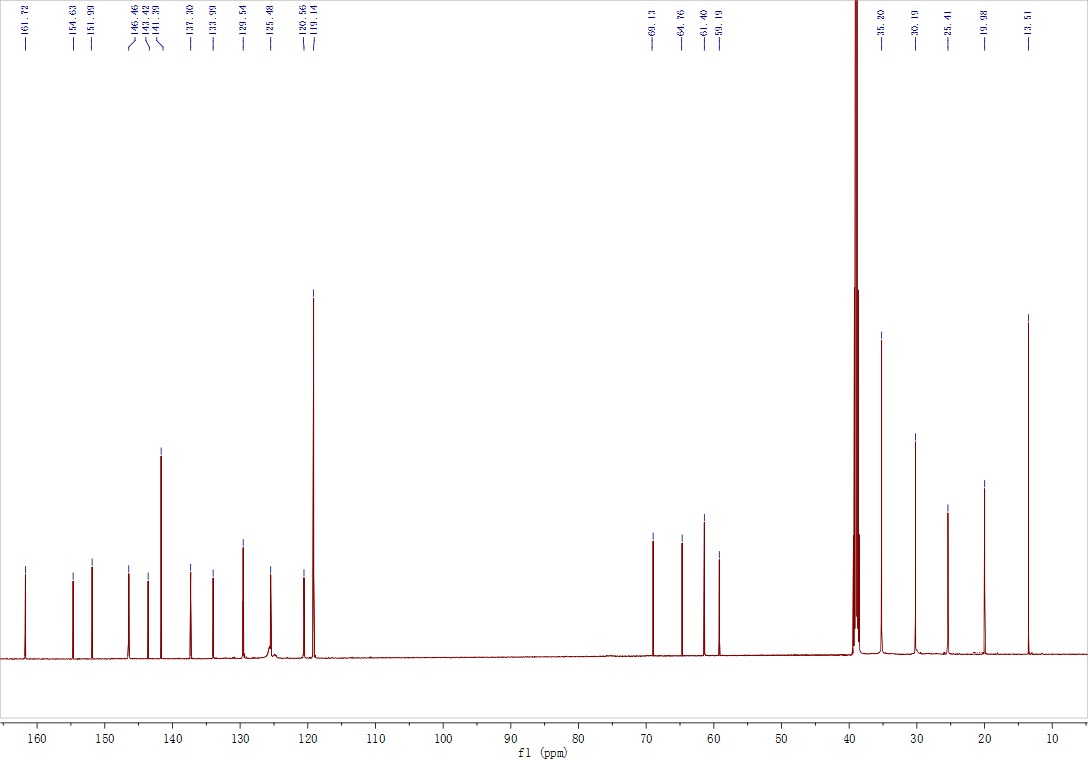
**

**
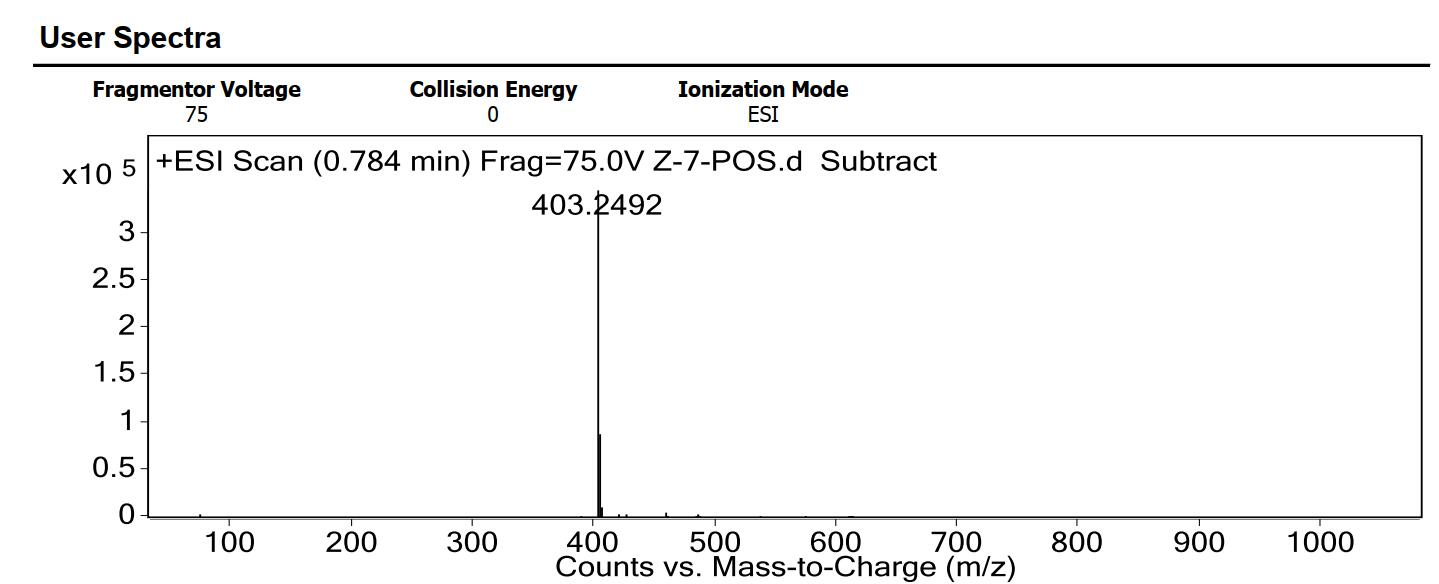
**

**11h**

**
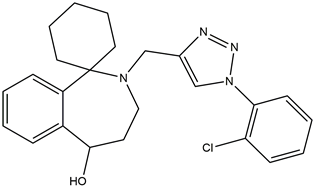
**

**
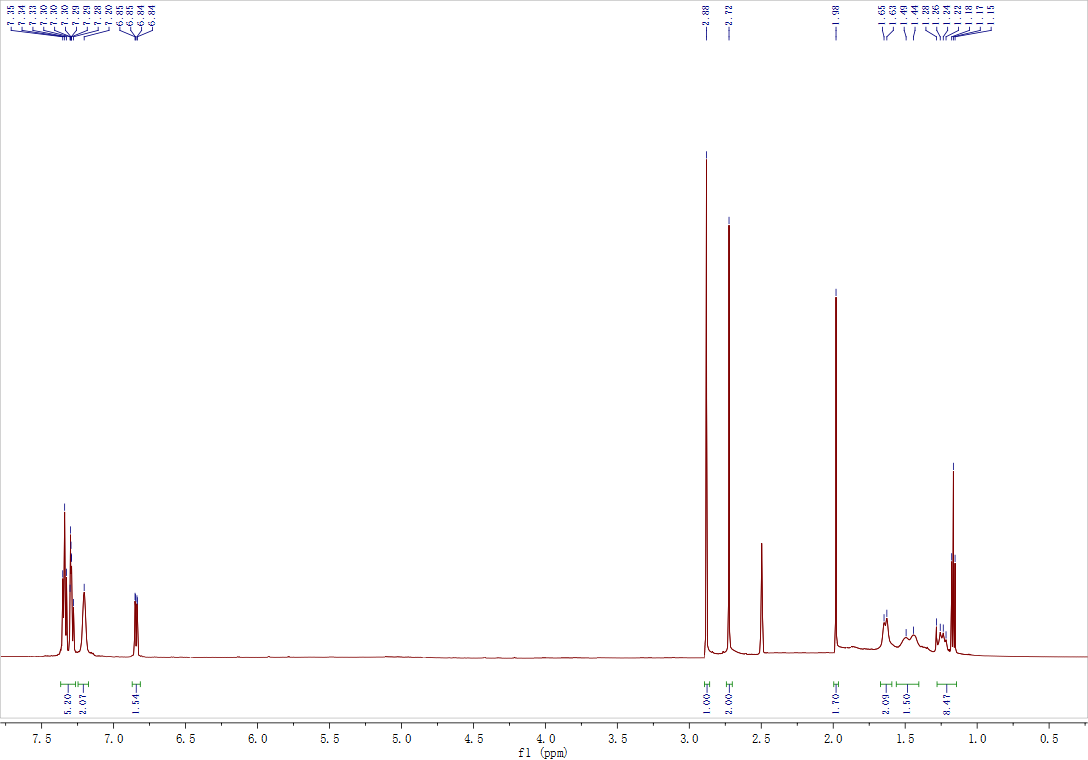
**

**
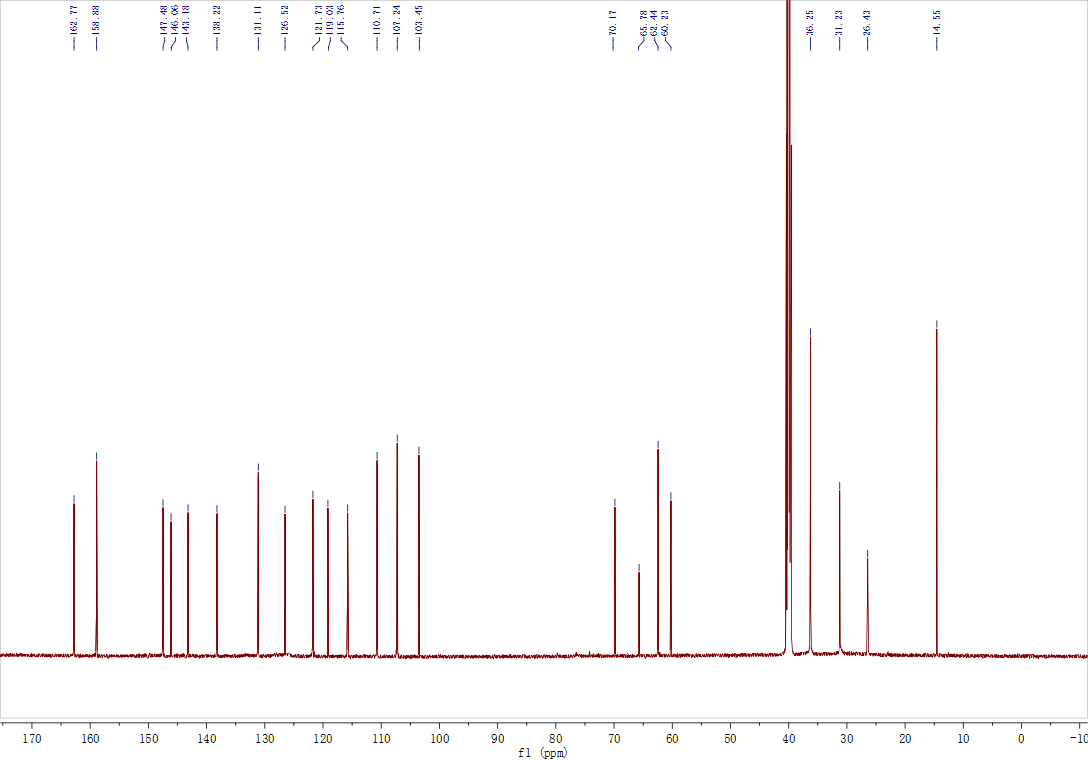
**

**
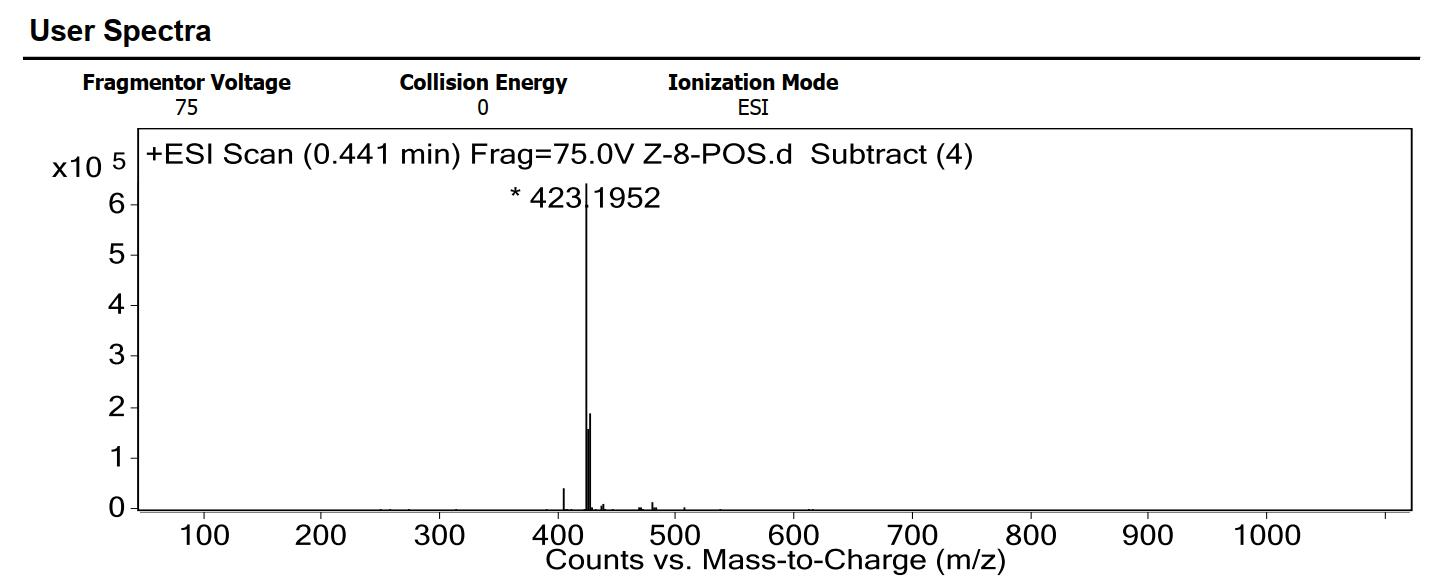
**

**11i**

**
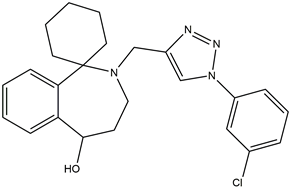
**

**
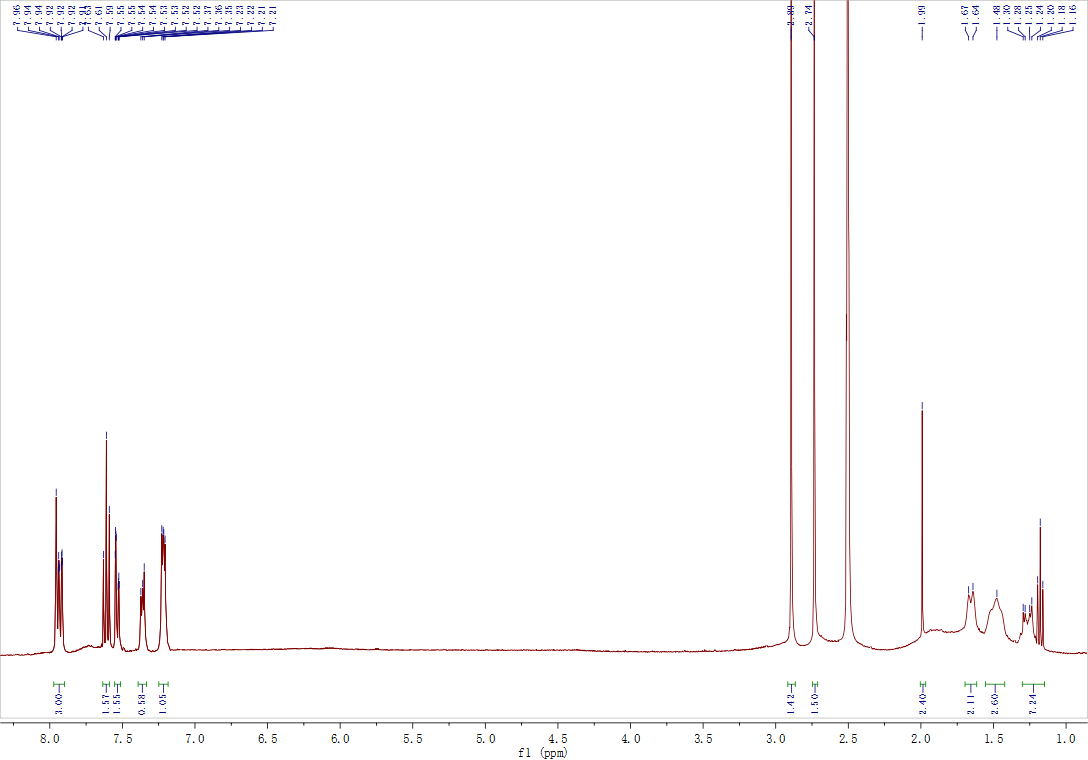
**

**
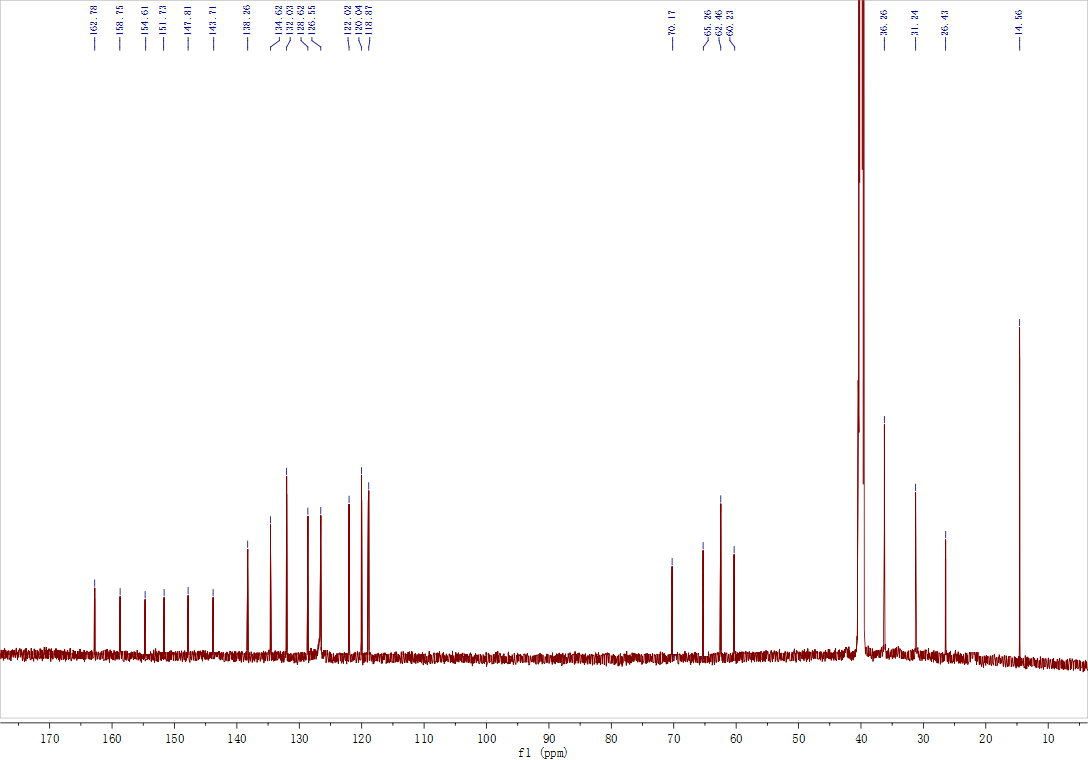
**

**
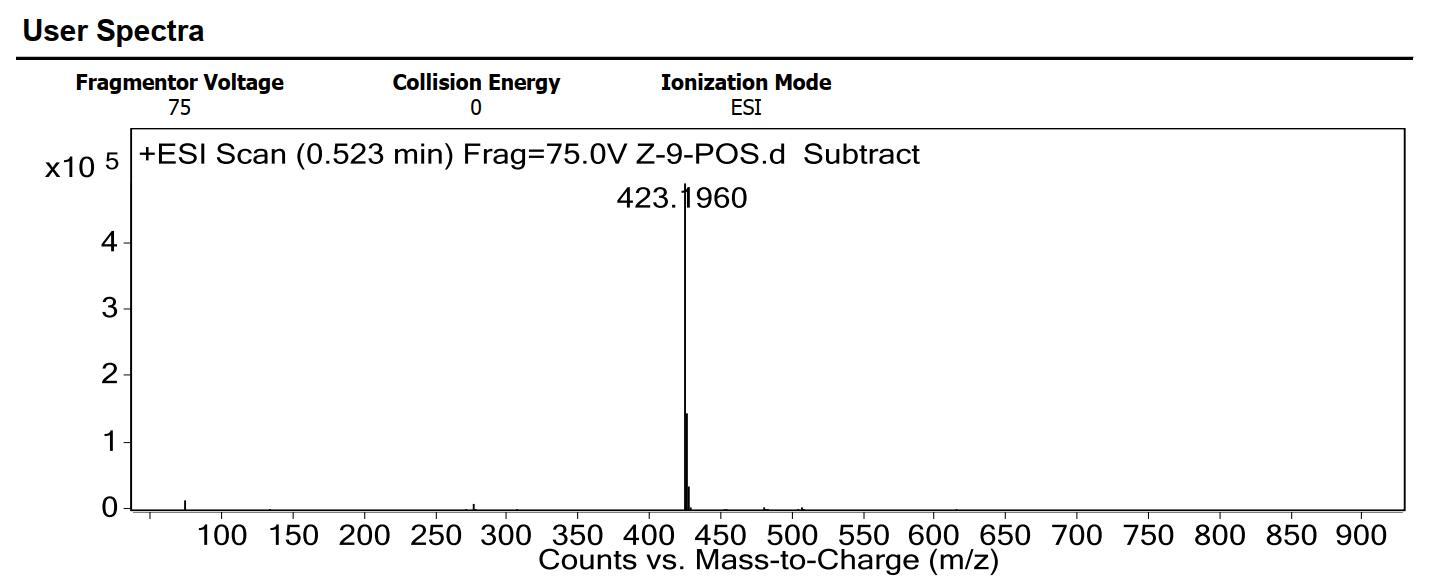
**

**11j**

**
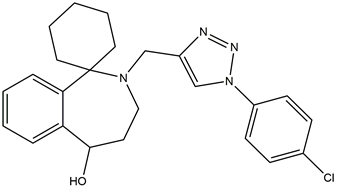

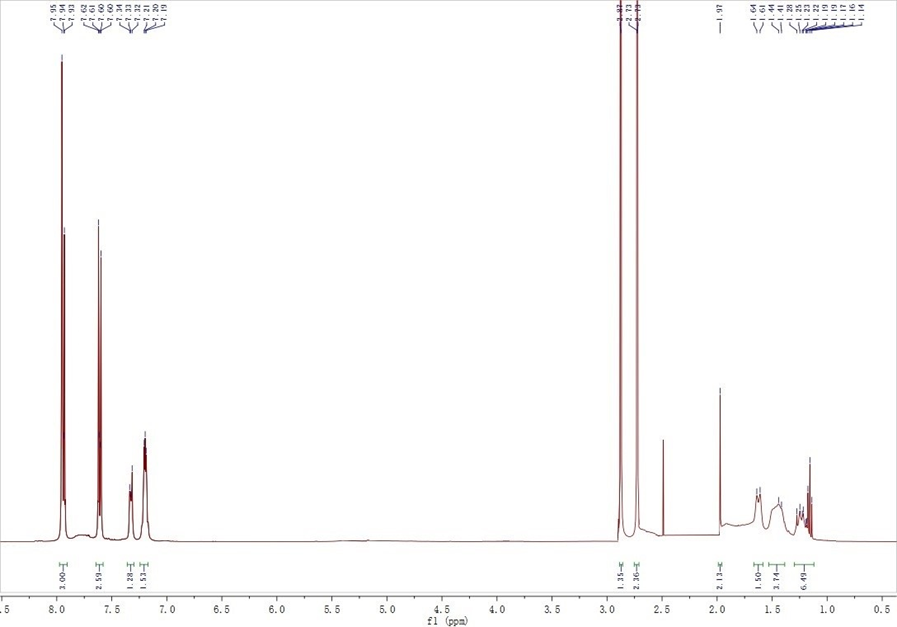
**


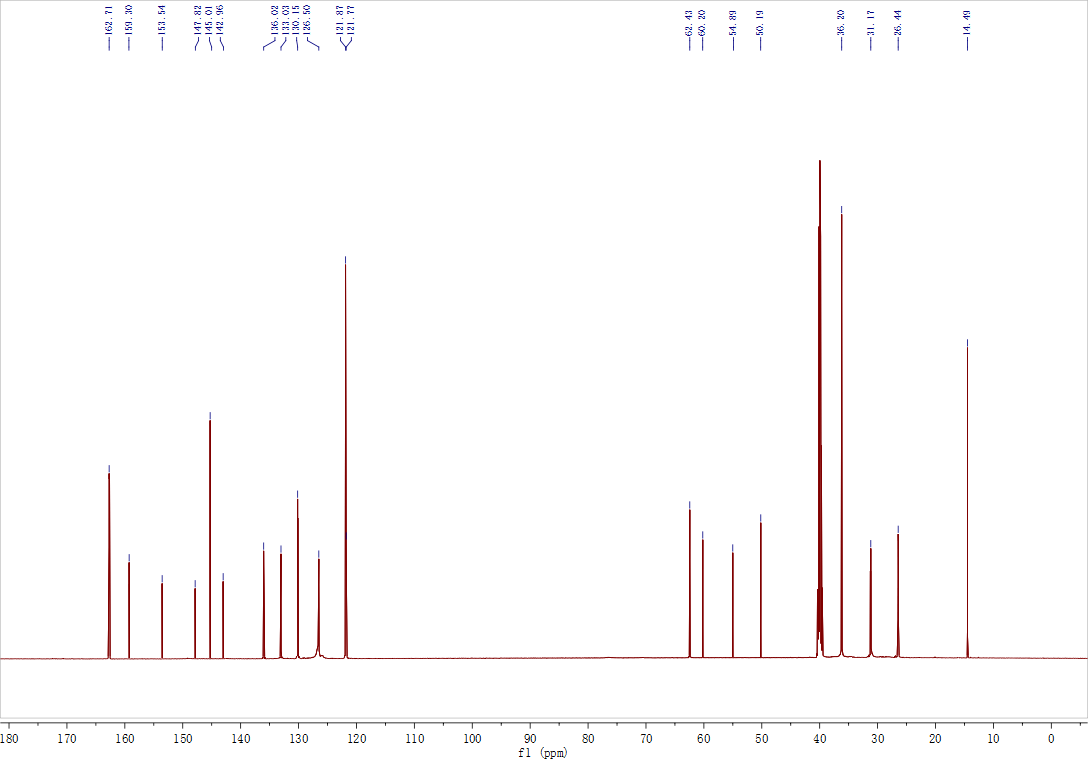


**
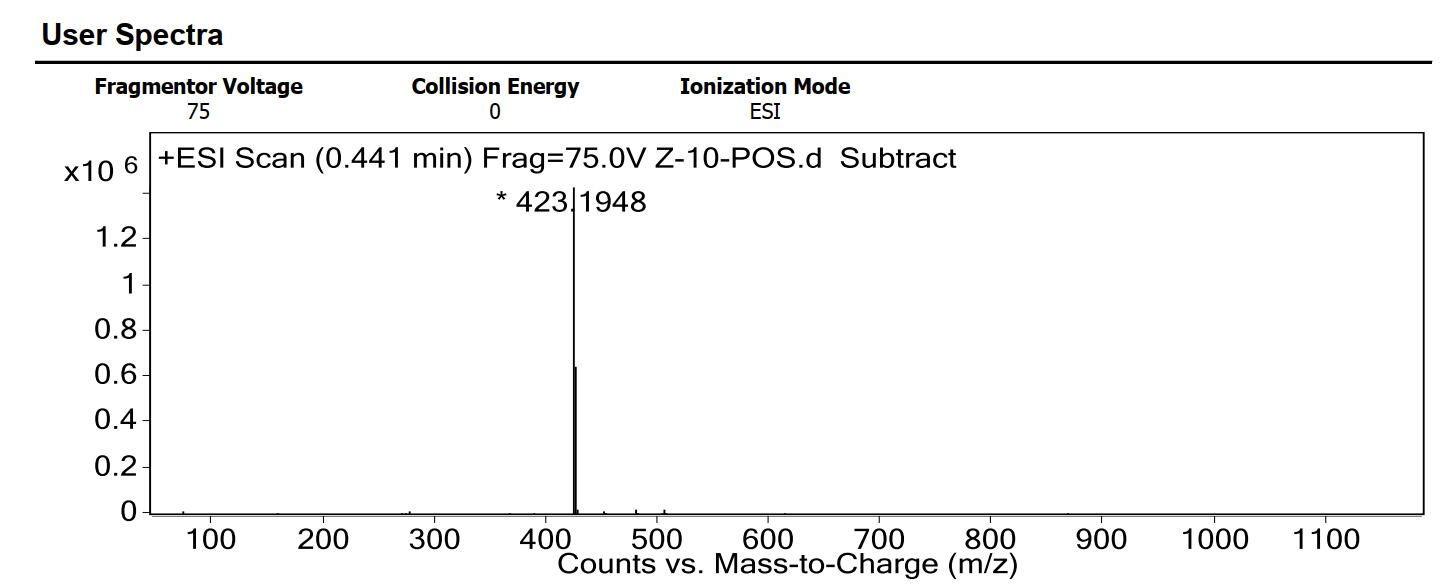
**

**11k**

**
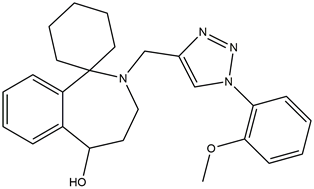
**

**
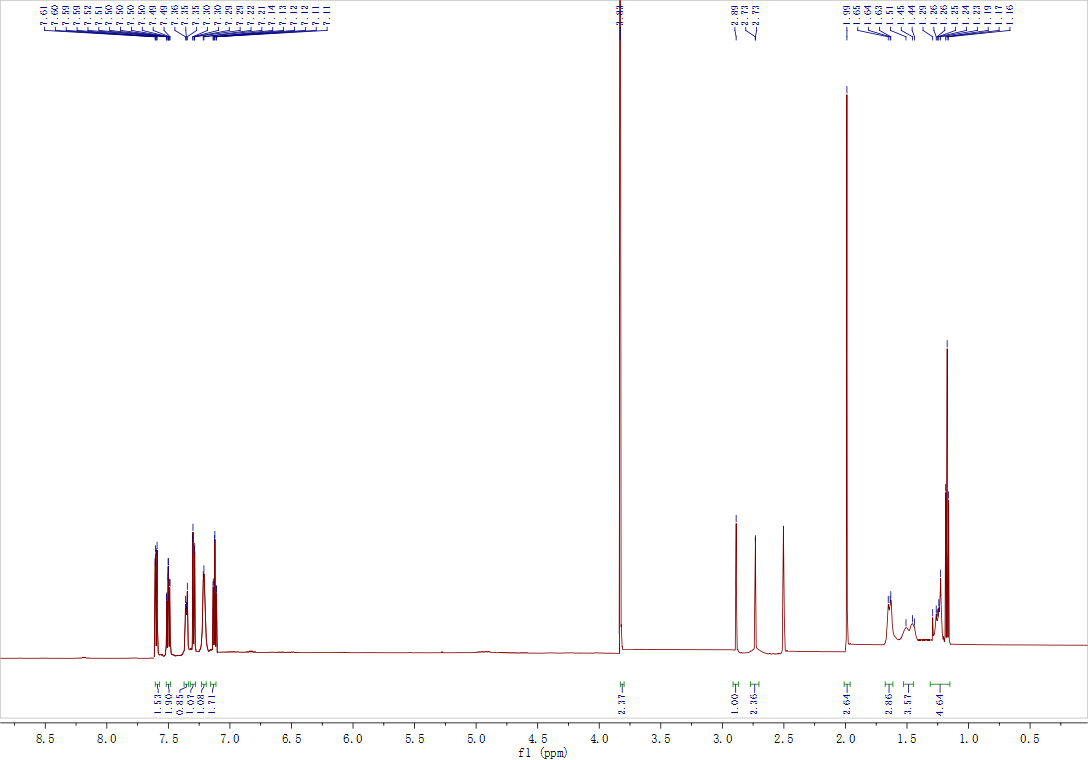
**

**
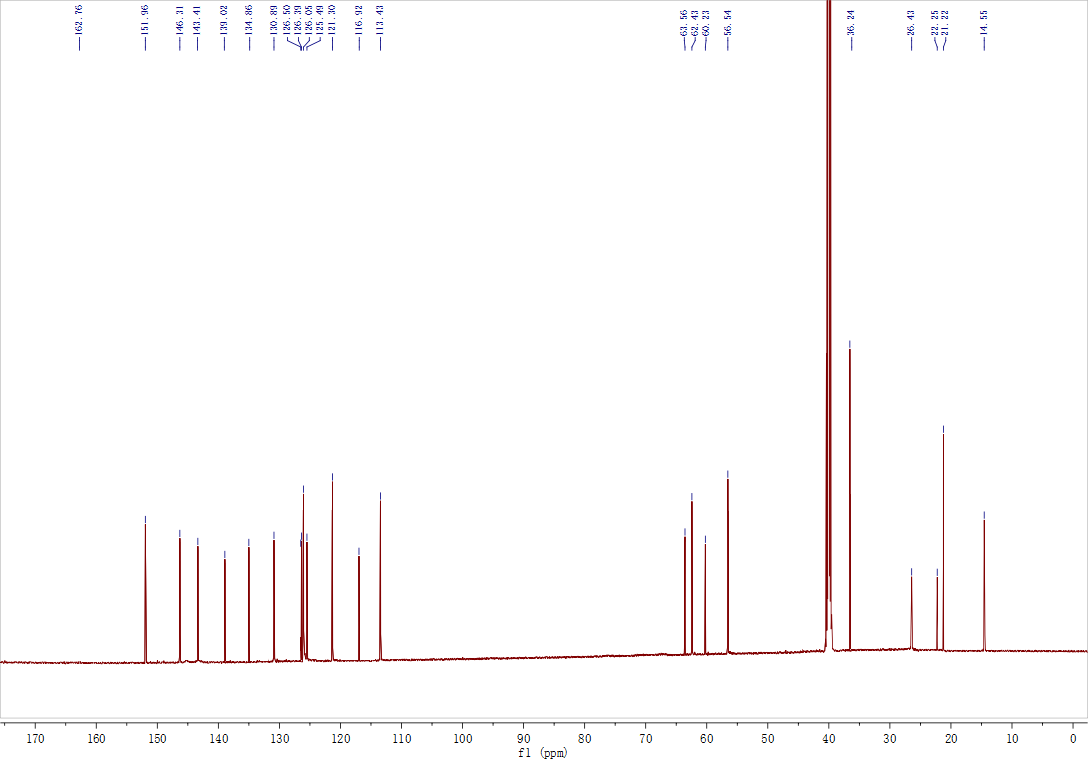
**

**
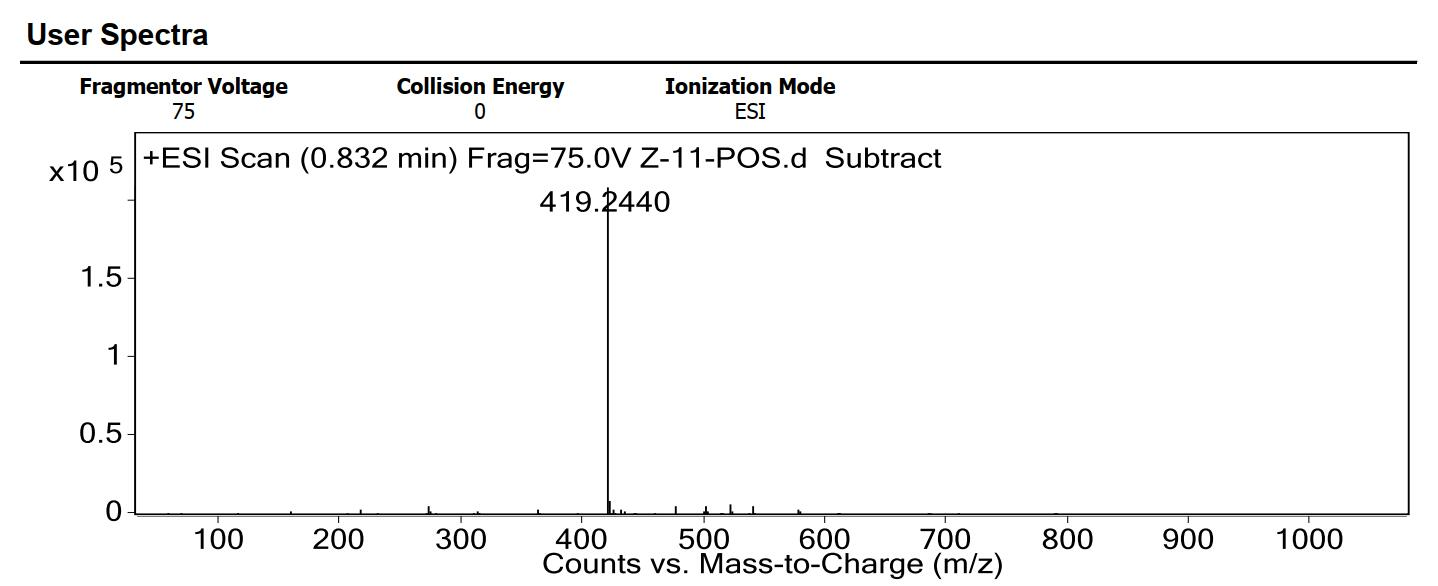
**

**11l**

**
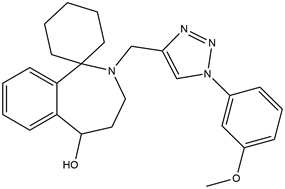
**

**
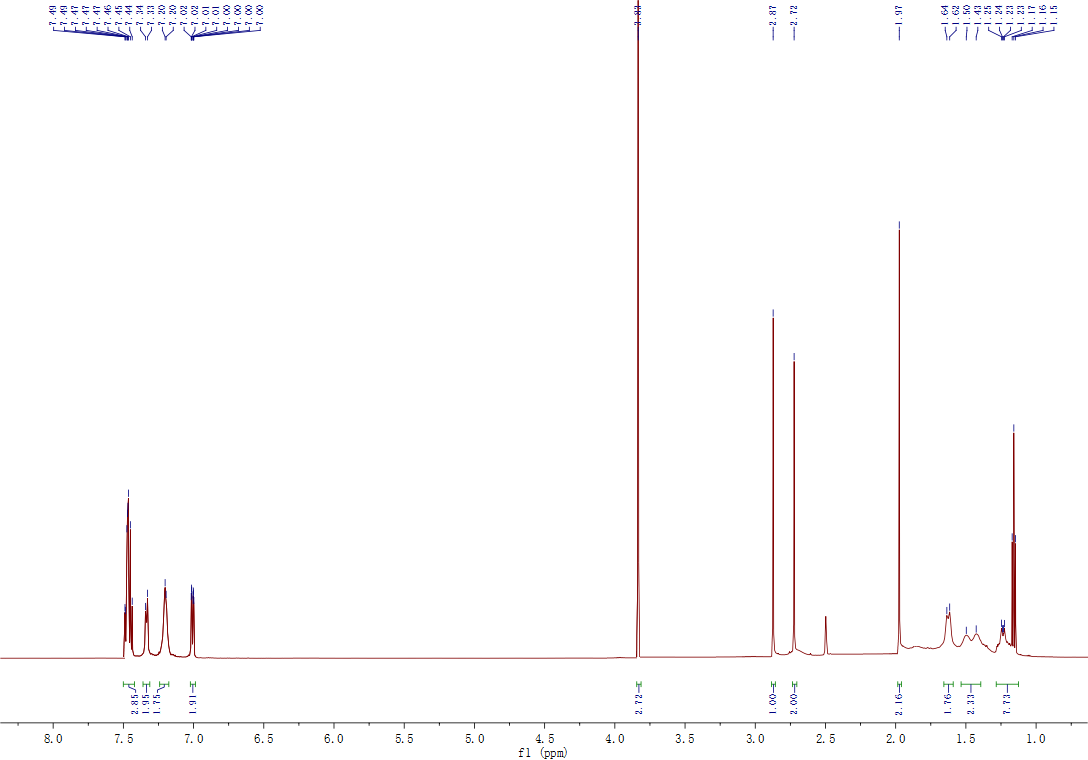
**

**
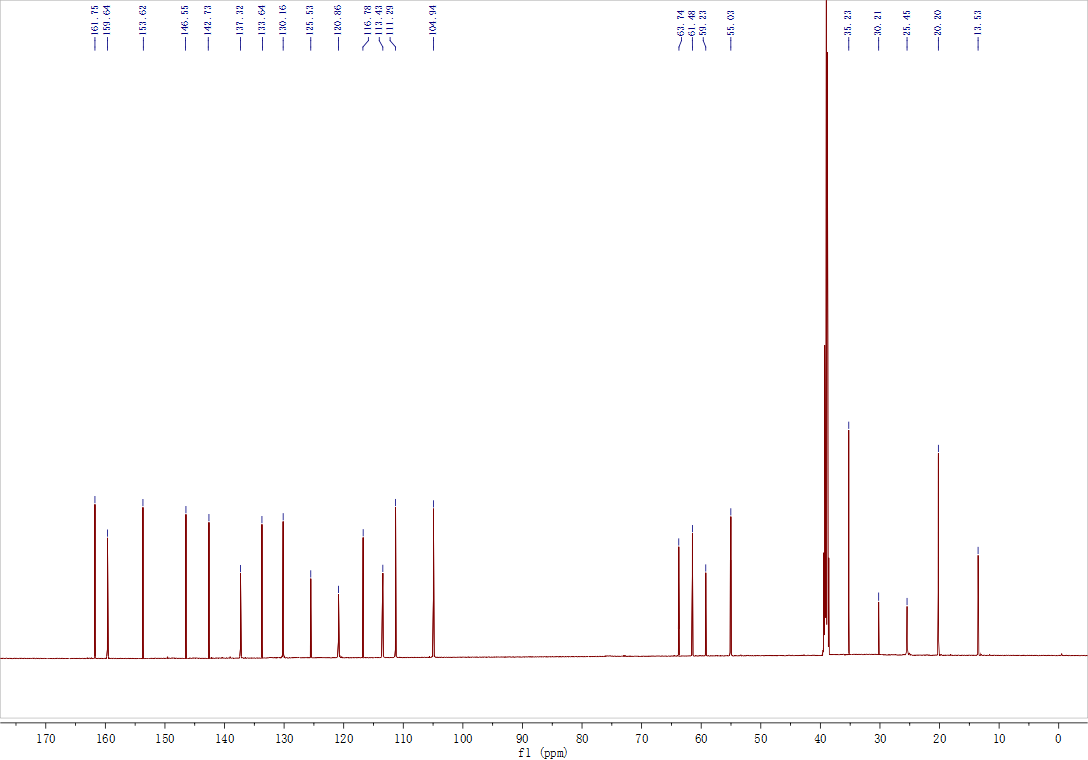
**

**
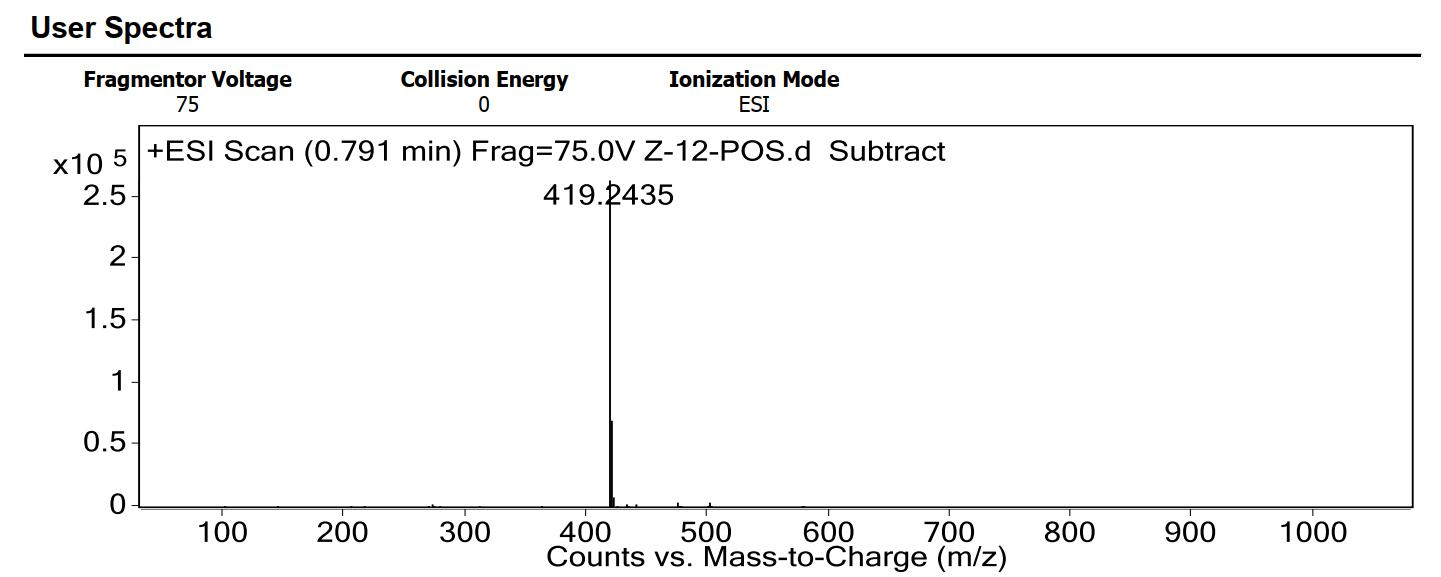
**

**11m**

**
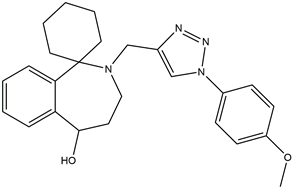
**

**
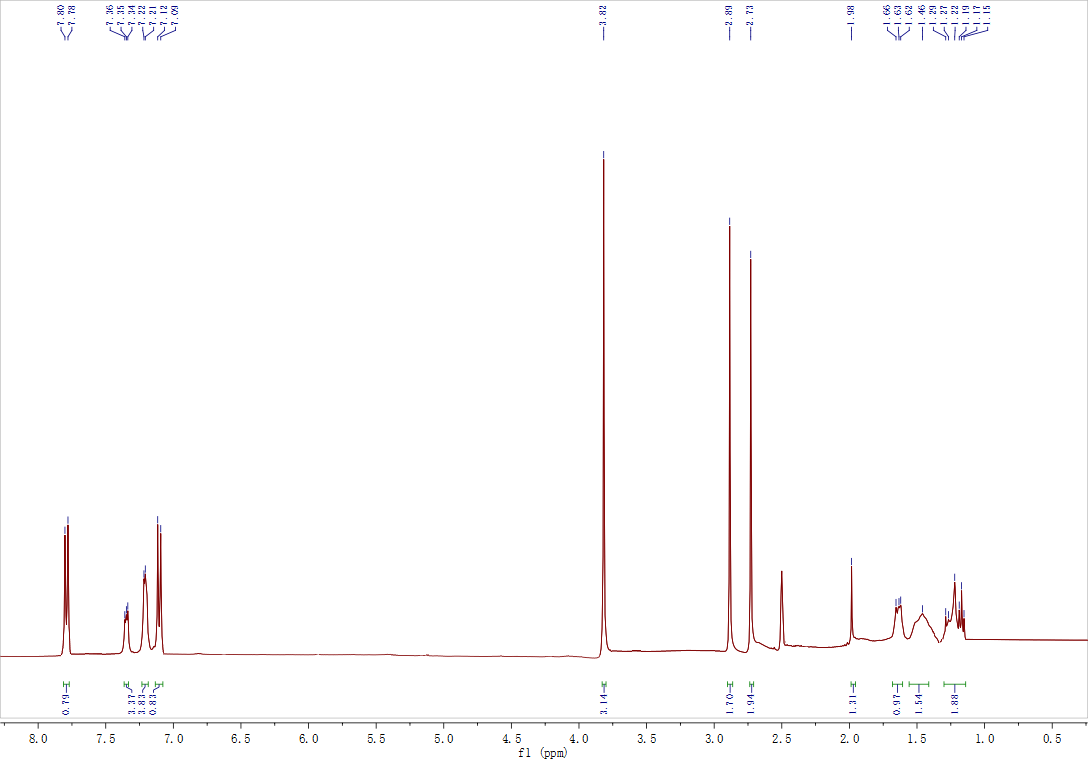
**

**
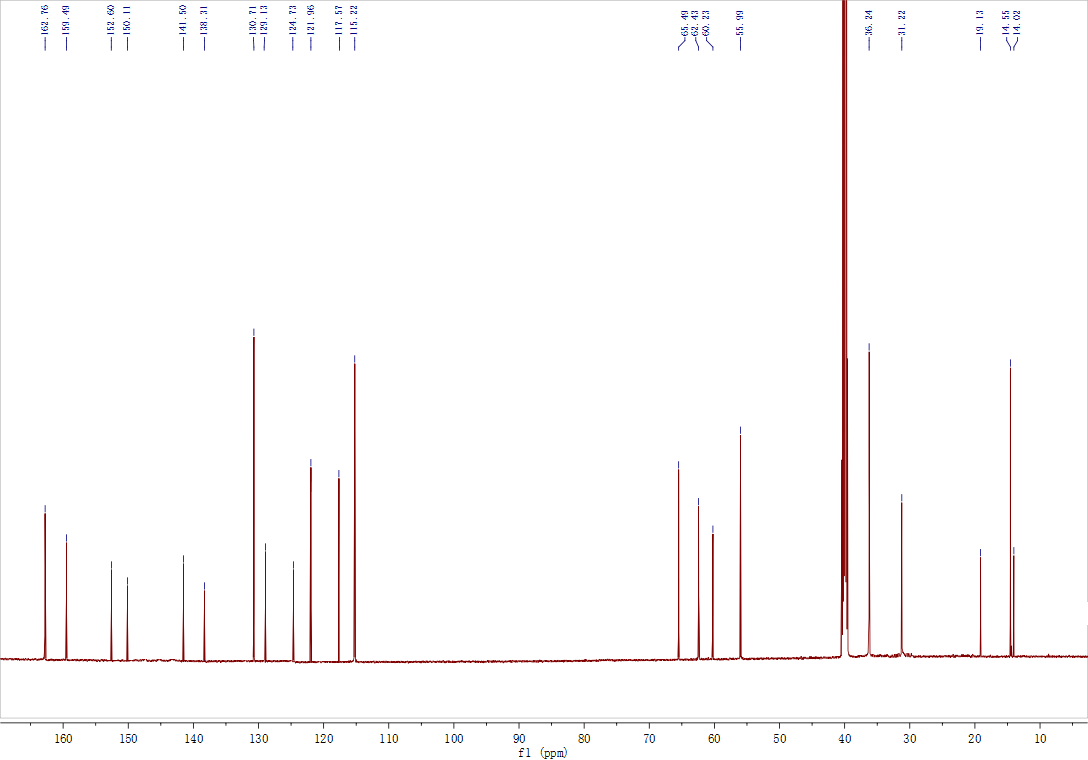
**

**
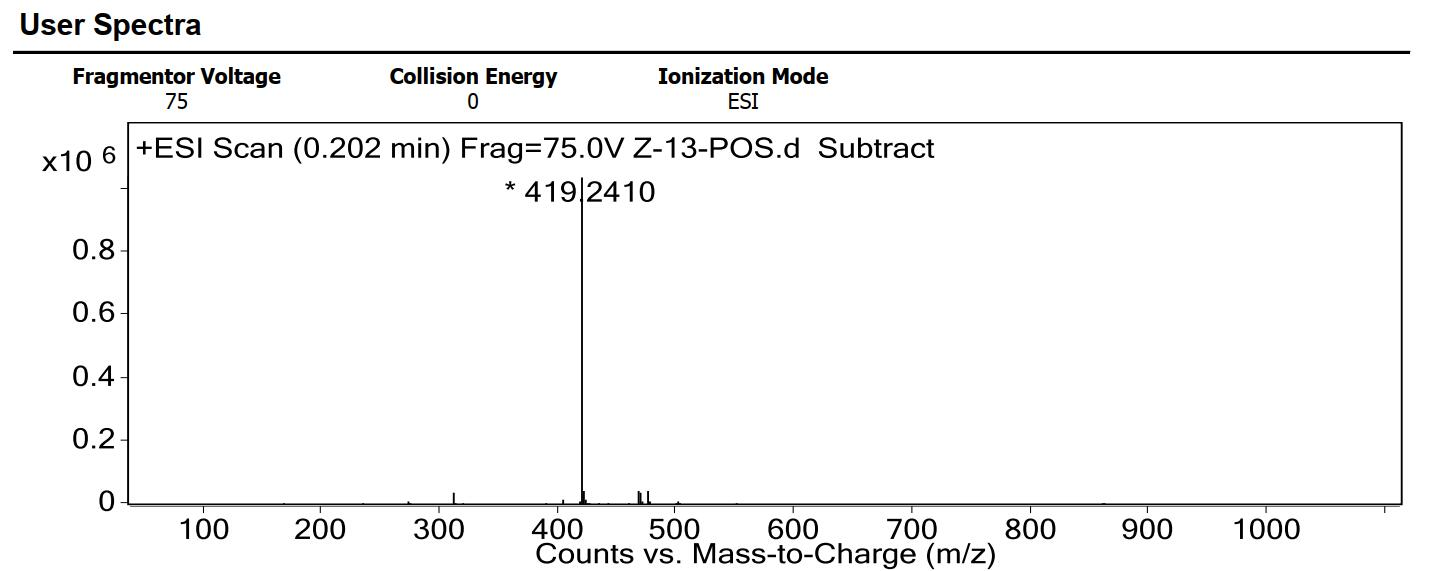
**

**11n**

**
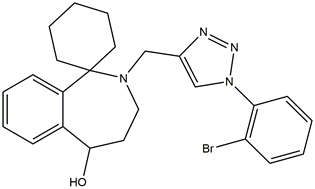
**

**
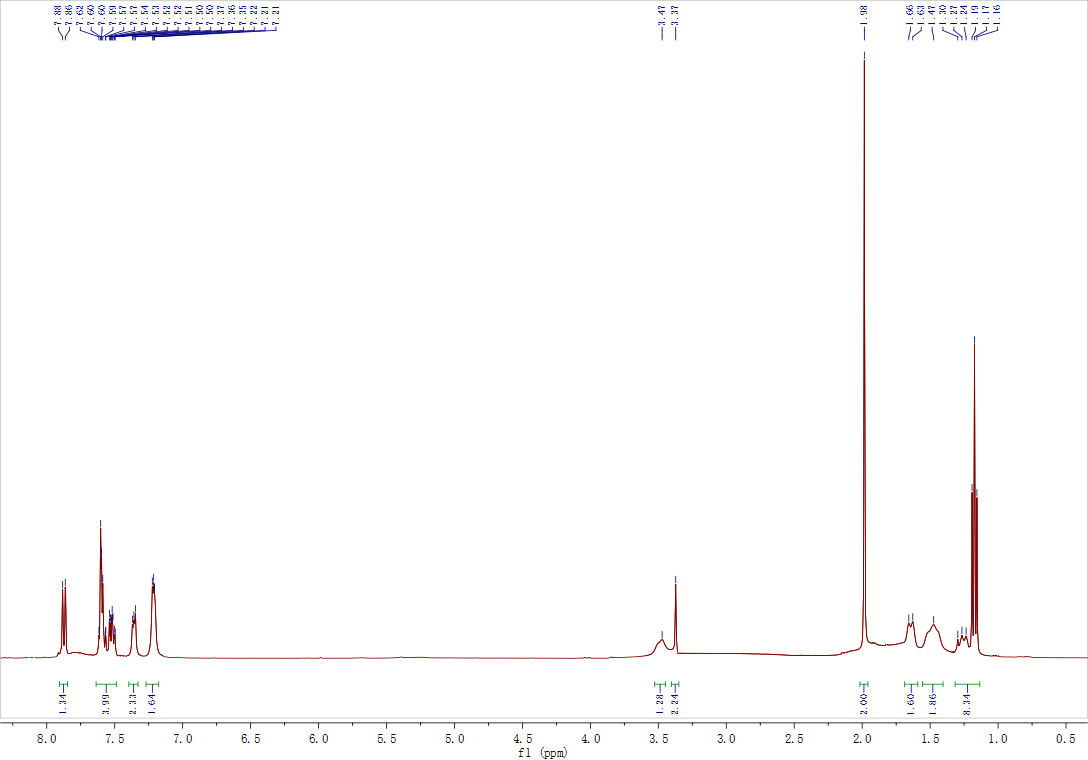
**

**
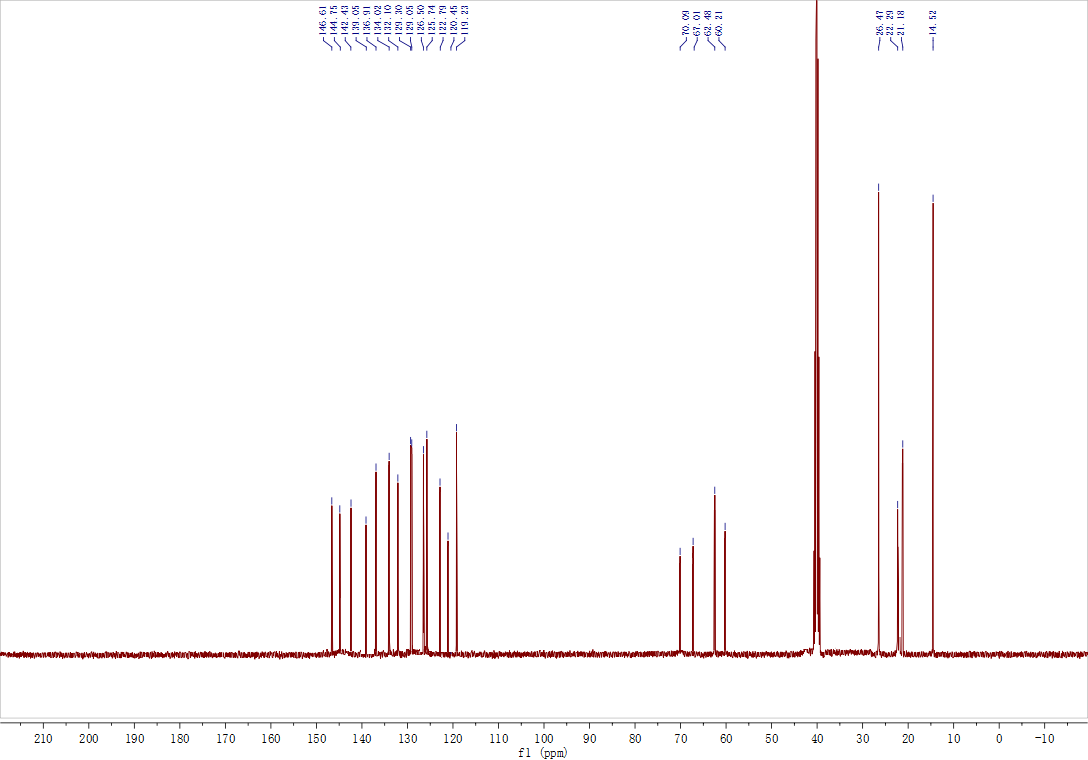
**

**
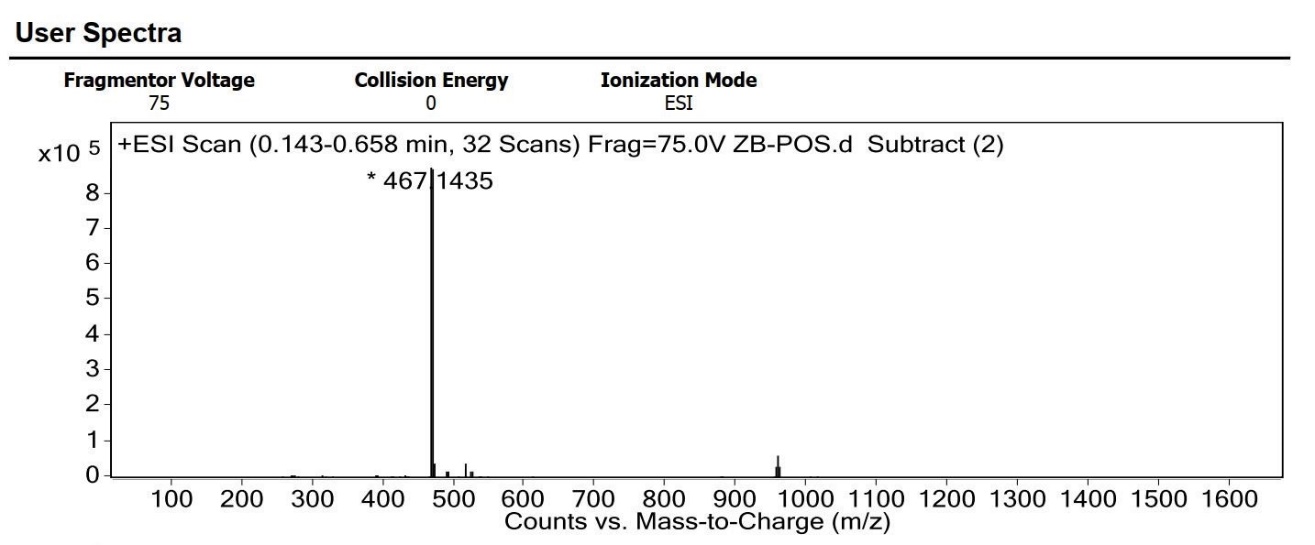
**

**11o**

**
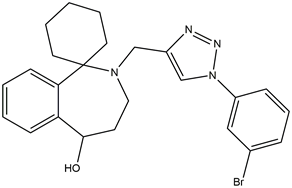
**

**
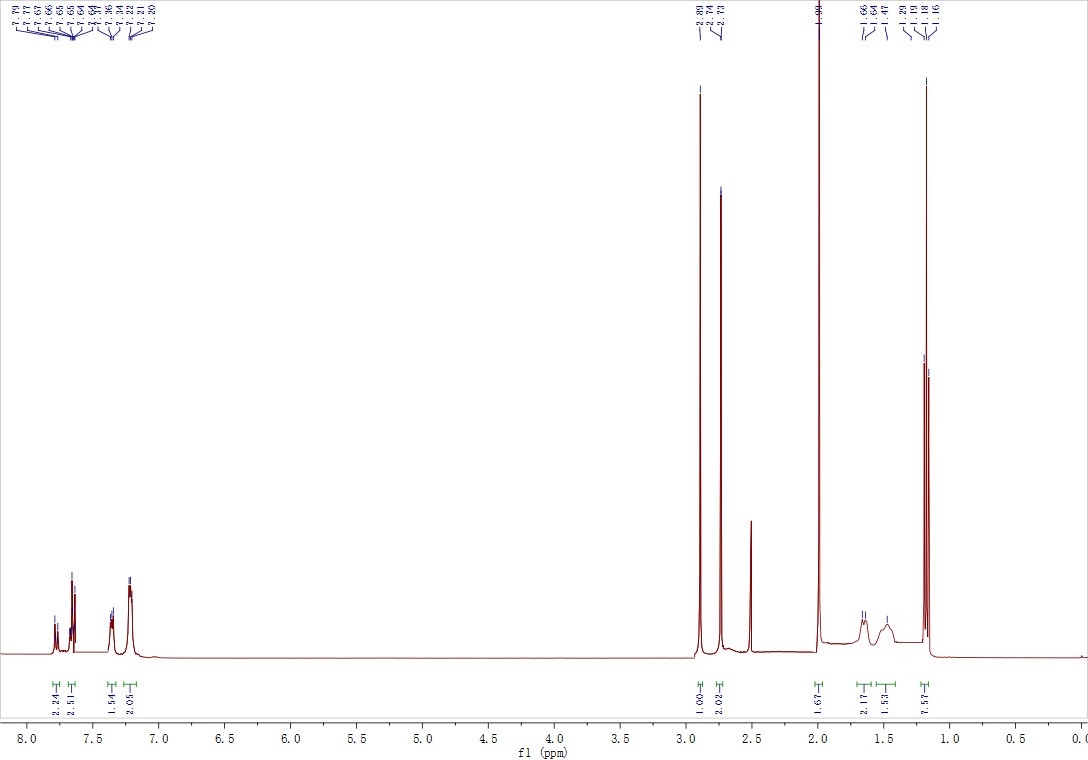
**

**
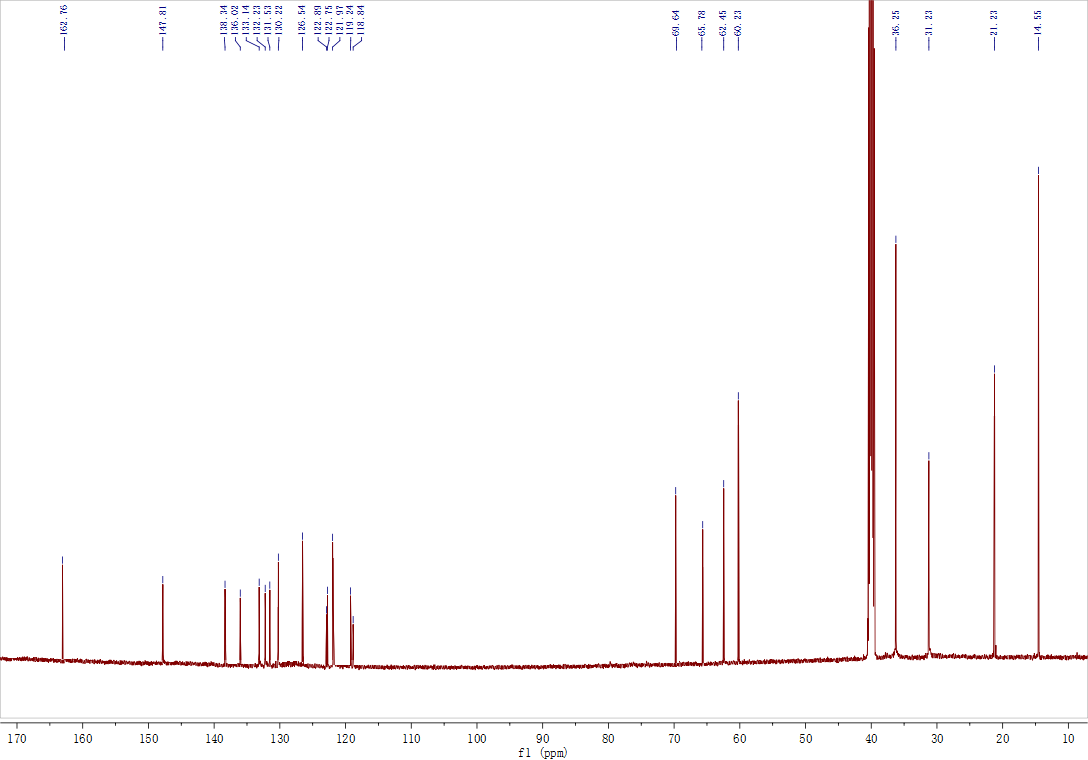
**

**
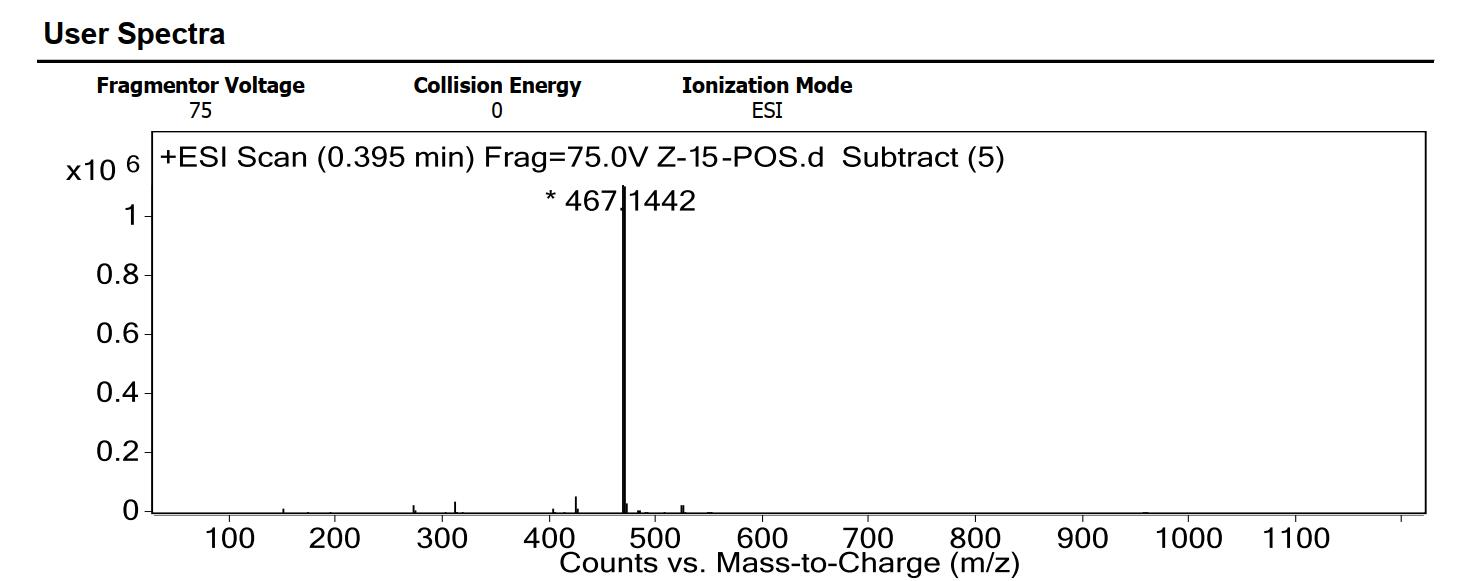
**

**11p**

**
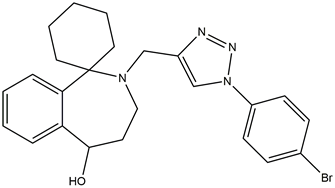
**

**
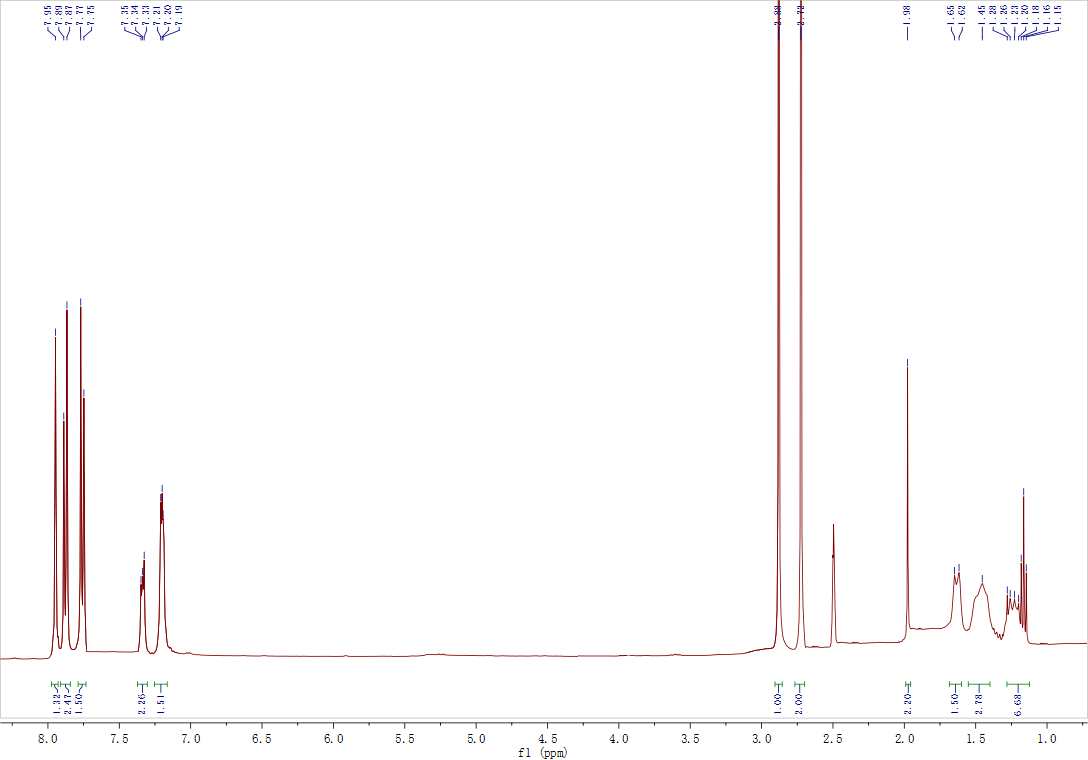
**

**
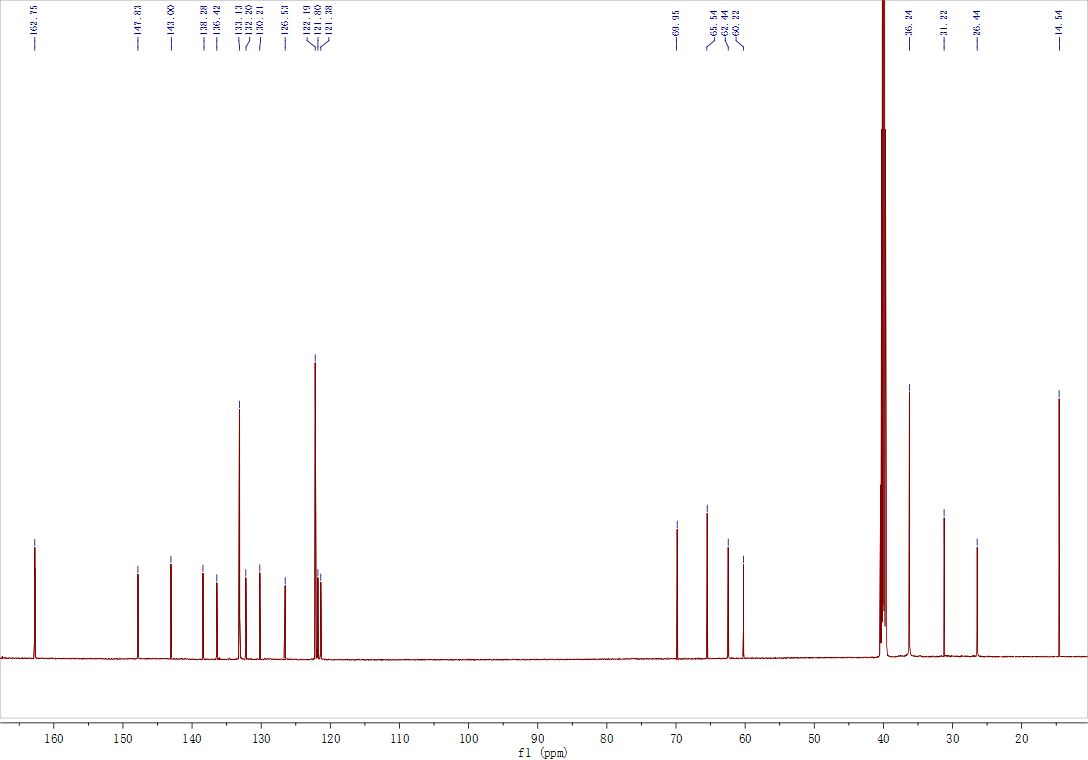
**

**
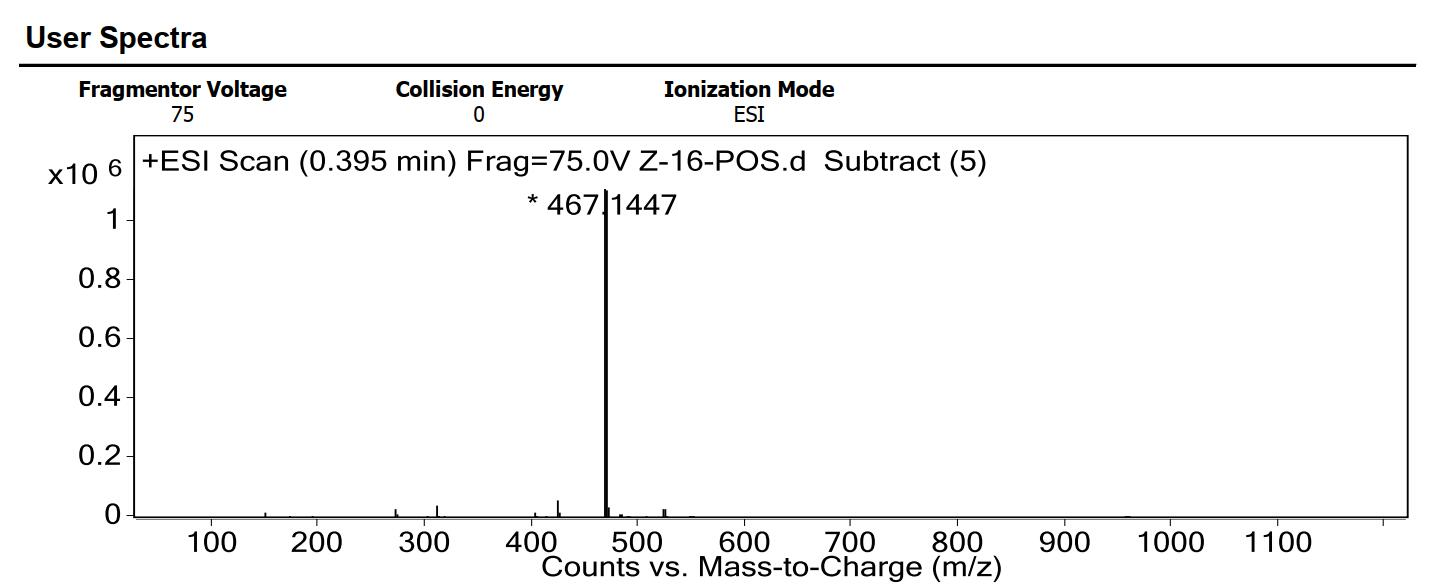
**

**11q**

**
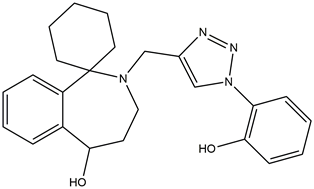
**

**
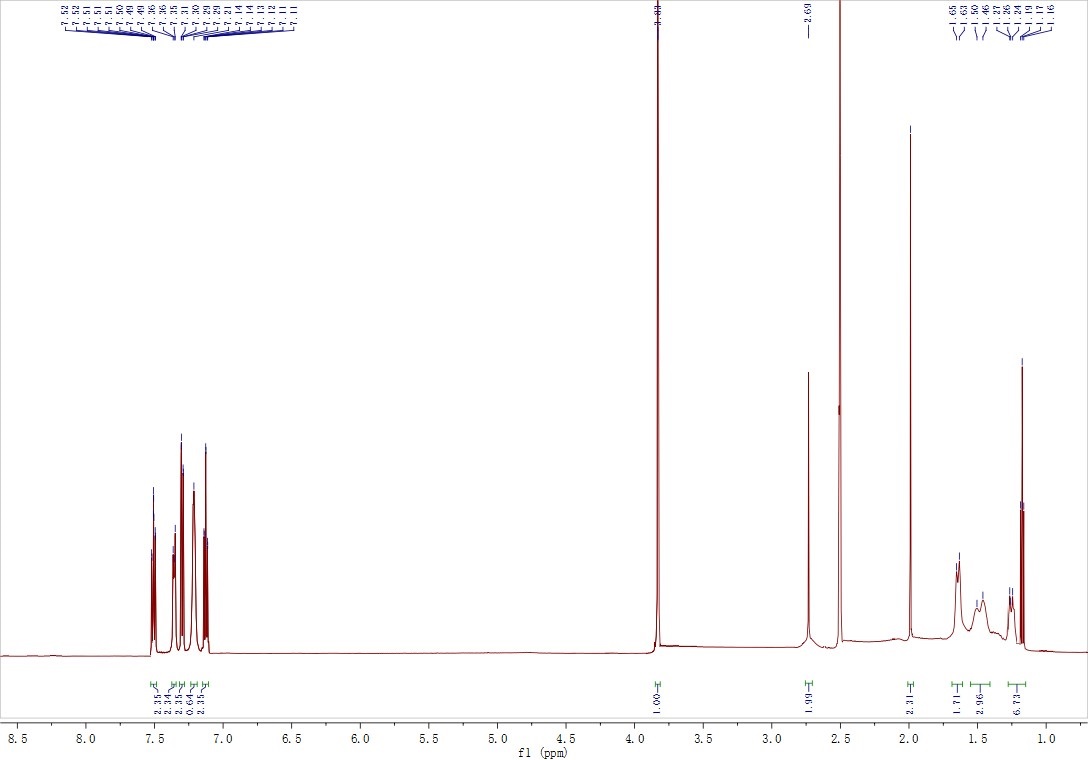
**


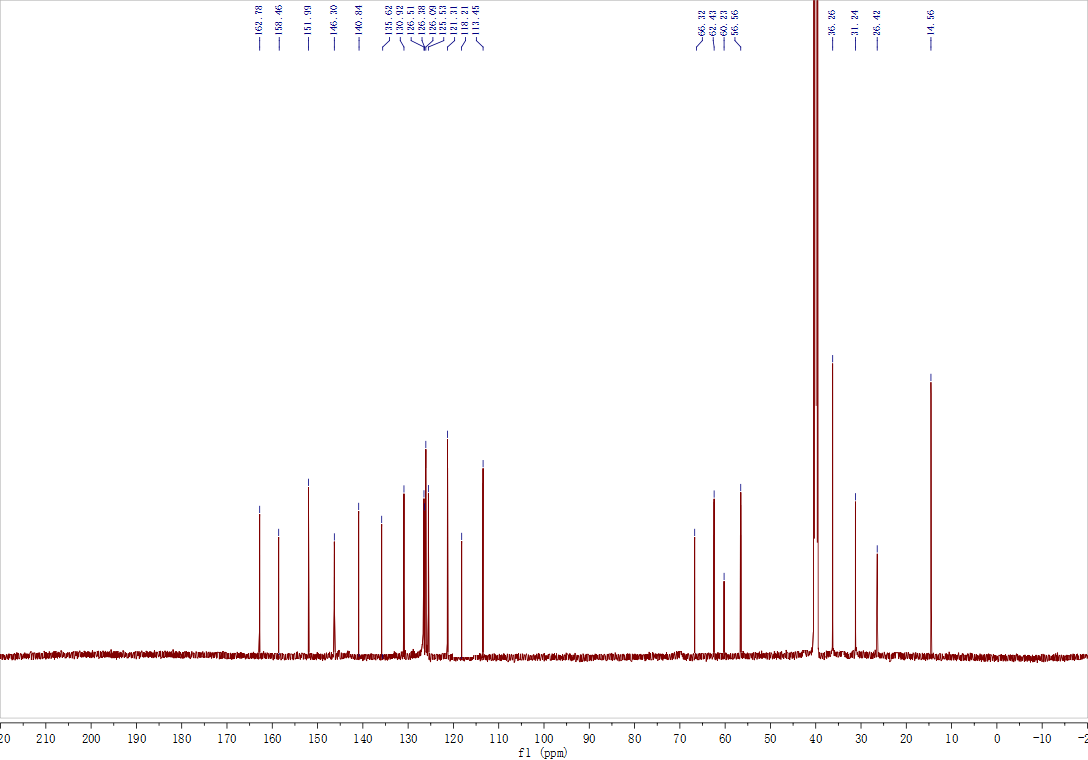


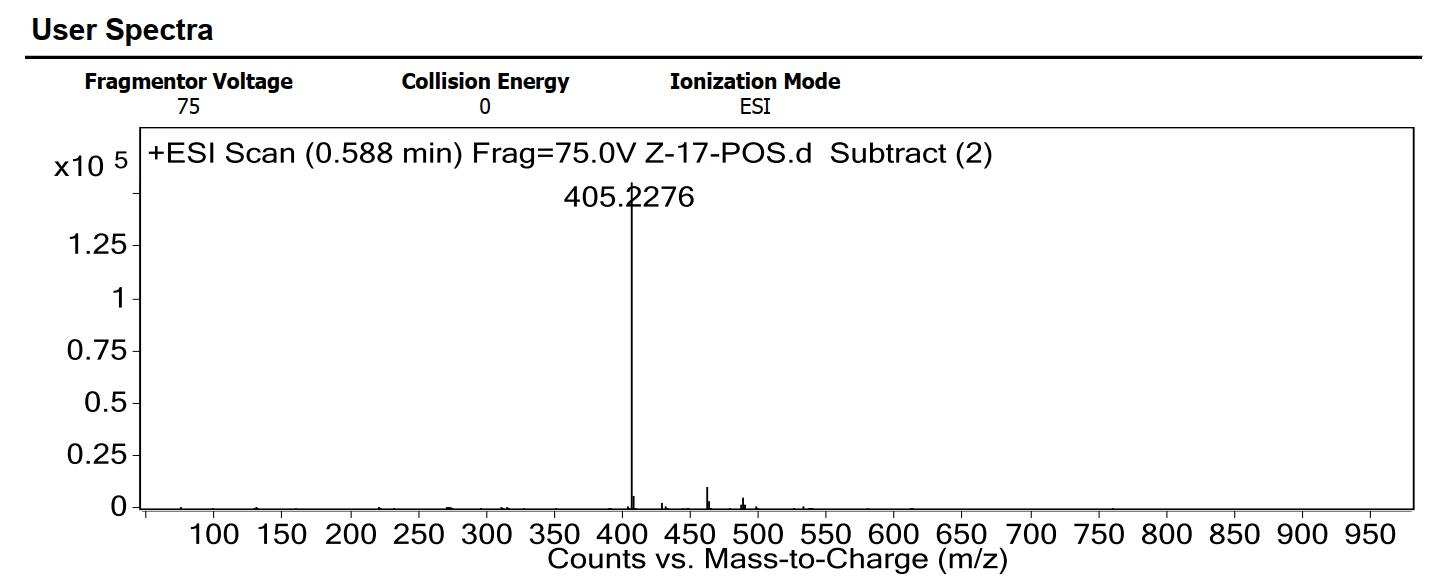


**11r**

**
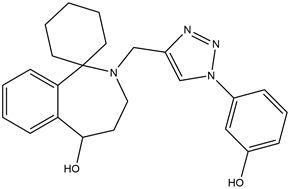
**

**
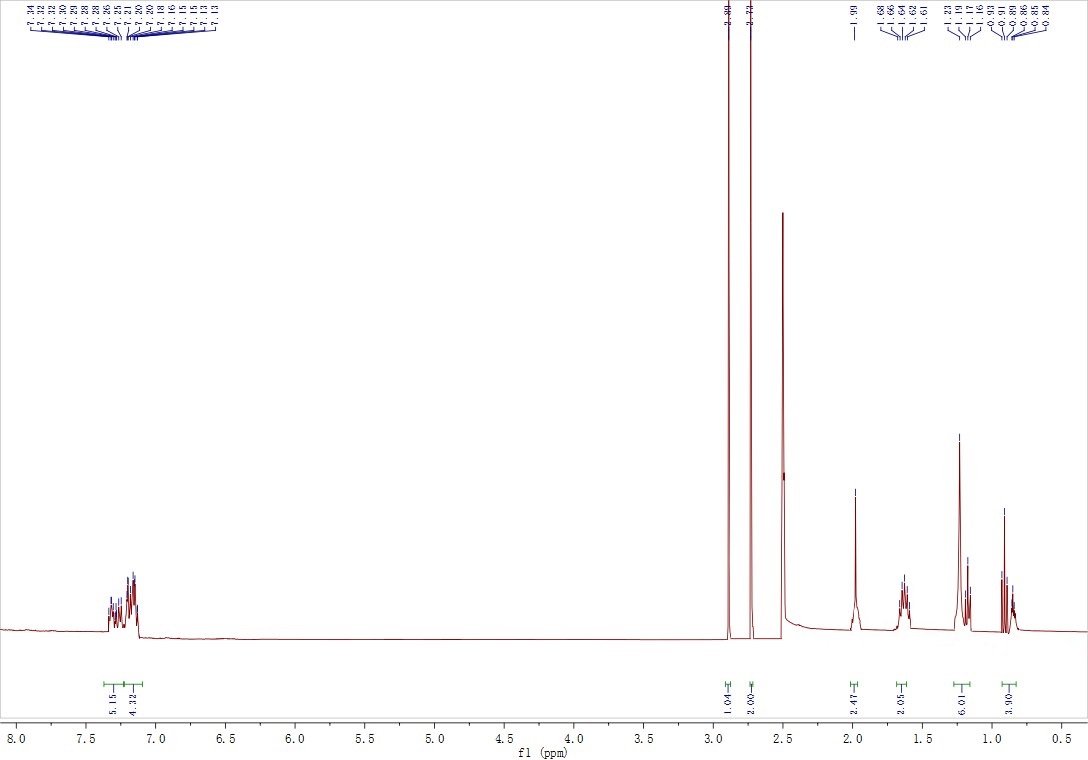
**

**
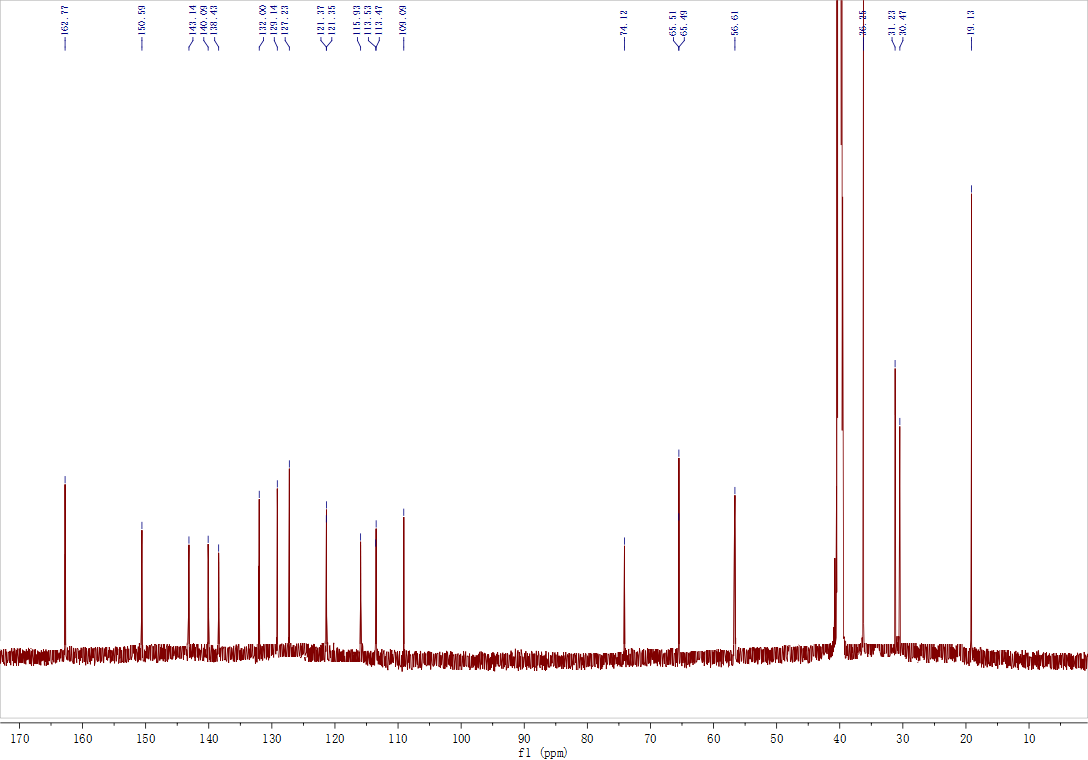
**

**
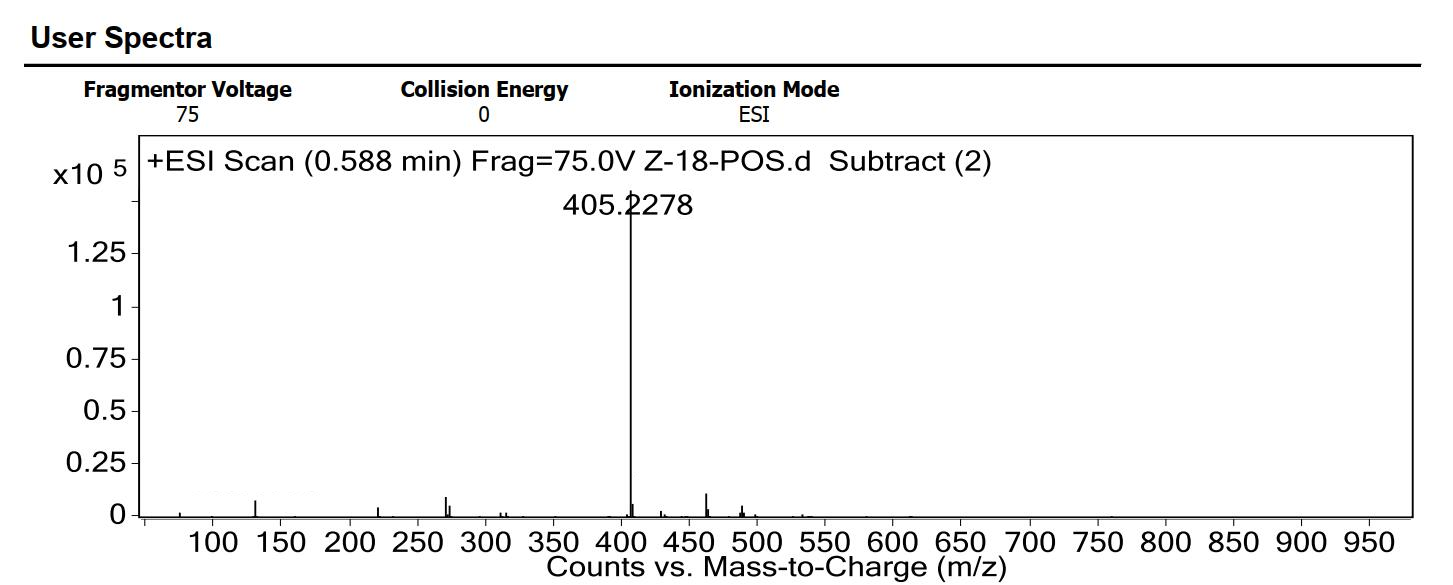
**

**11s**

**
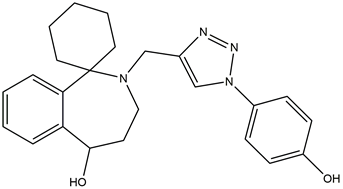
**

**
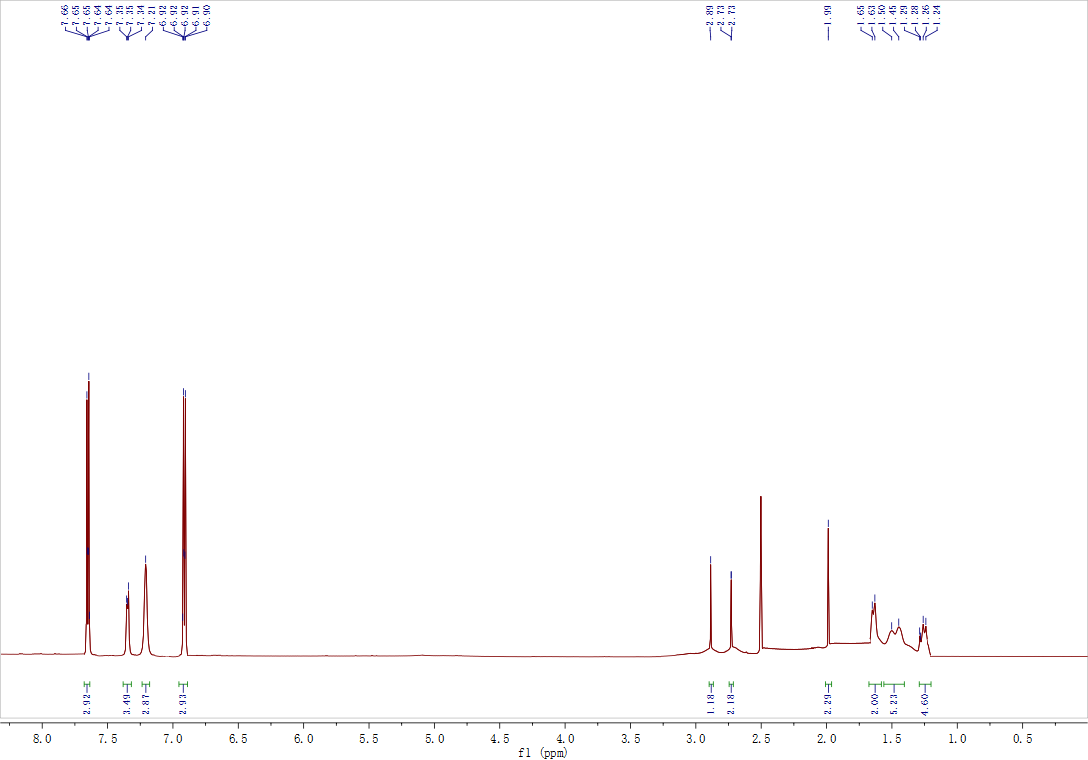
**

**
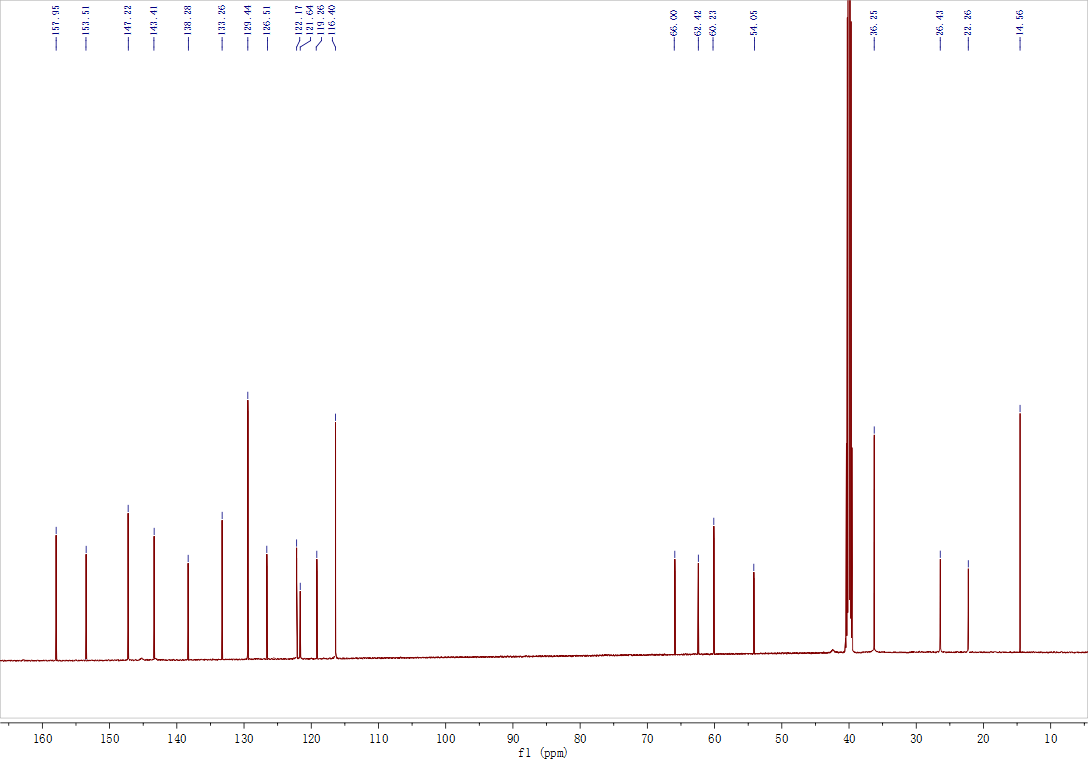
**


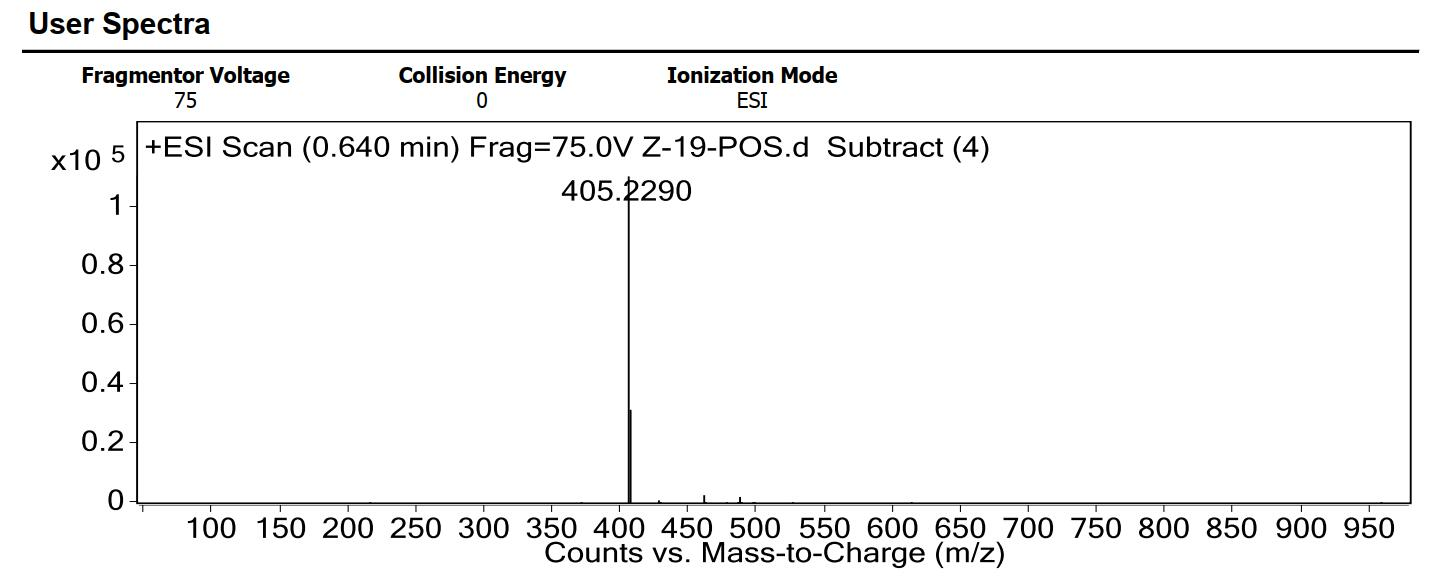


**11t**

**
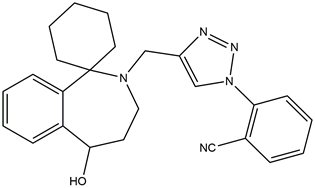
**

**
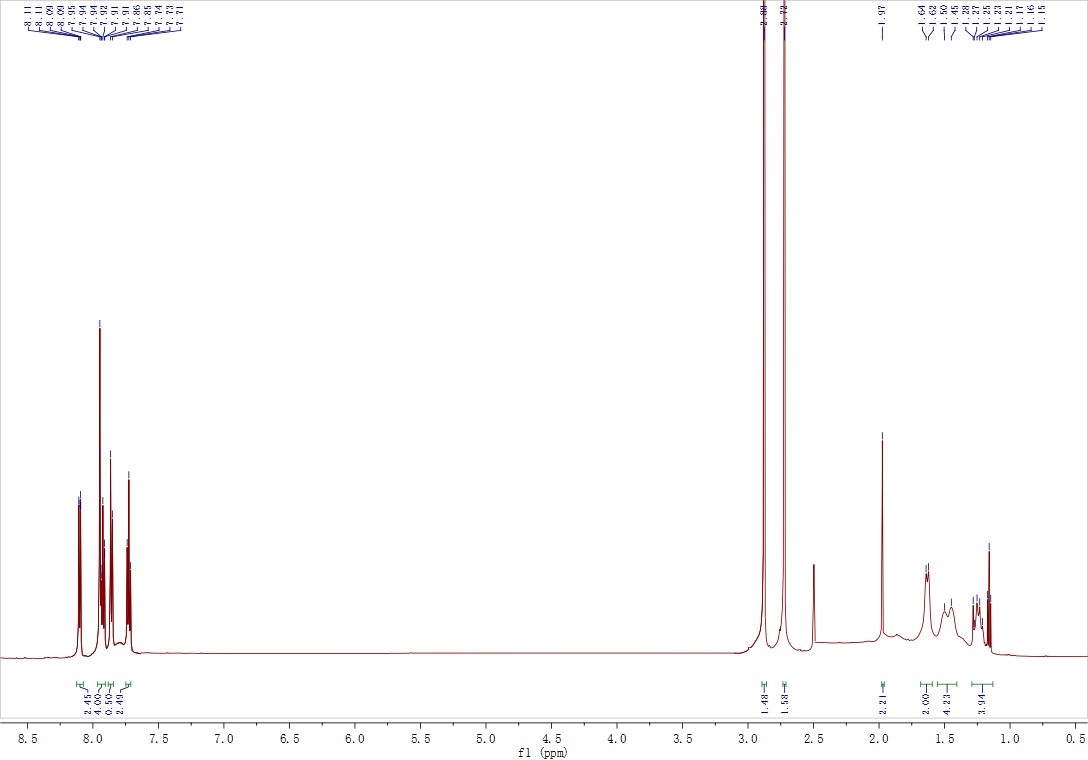
**

**
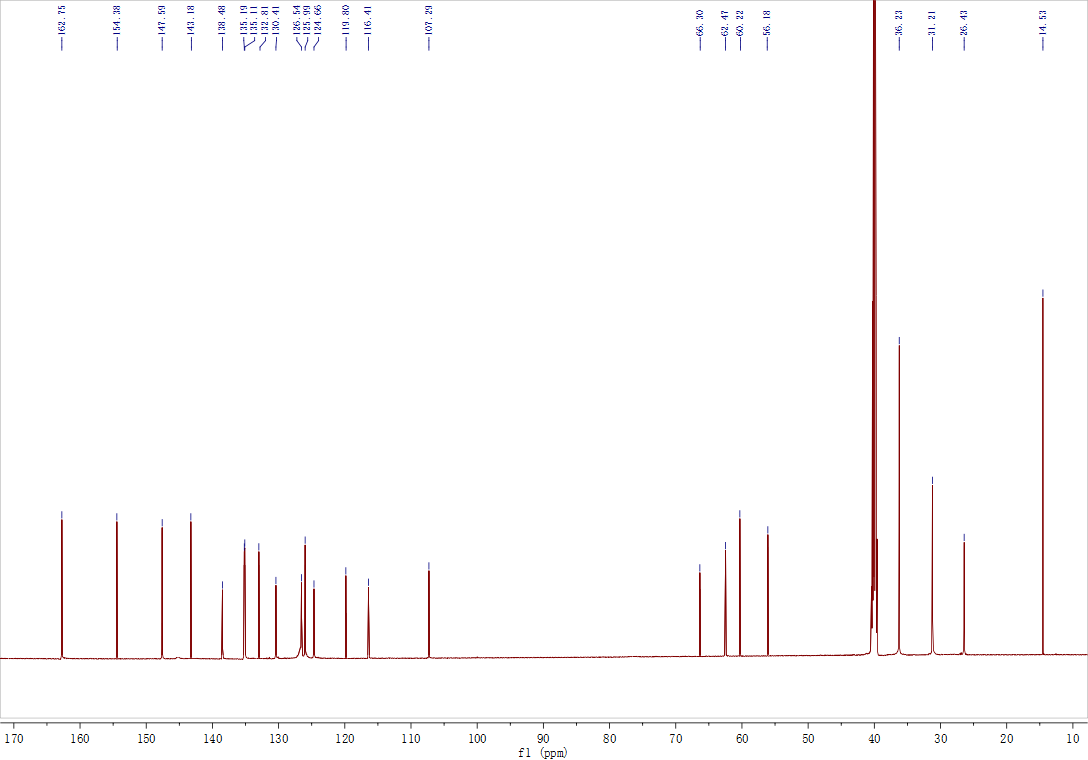
**

**
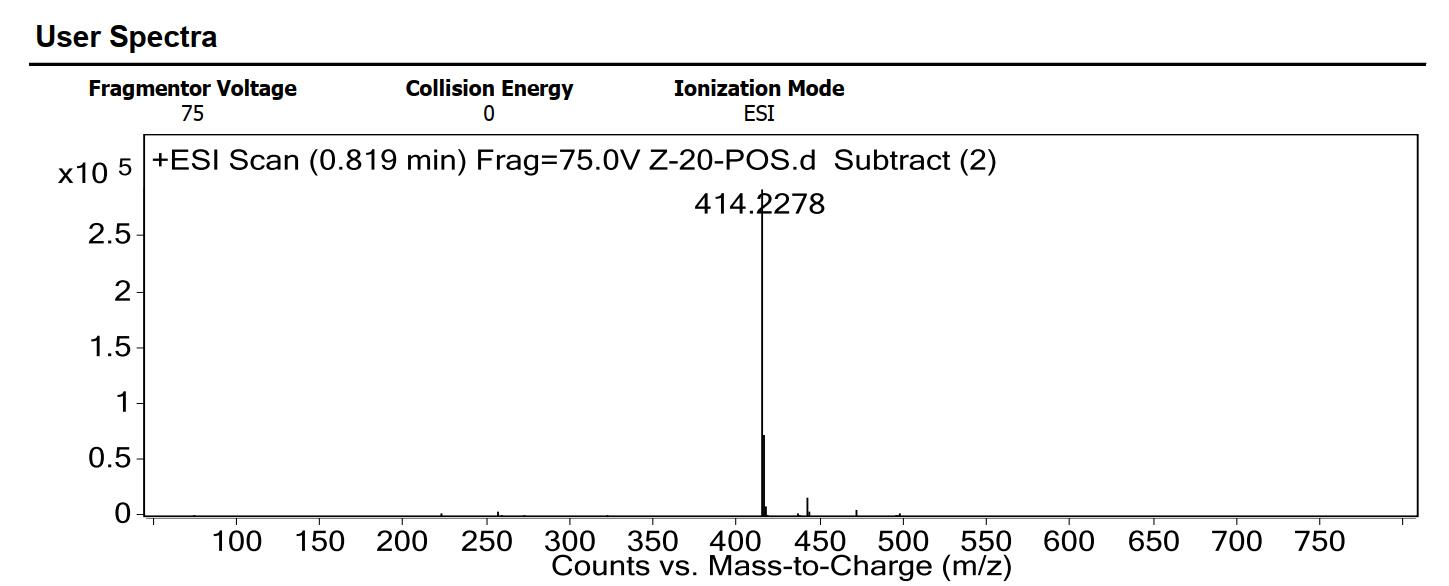
**

**11u**

**
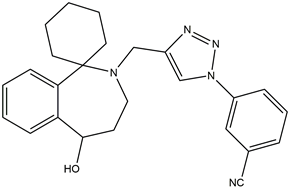
**

**
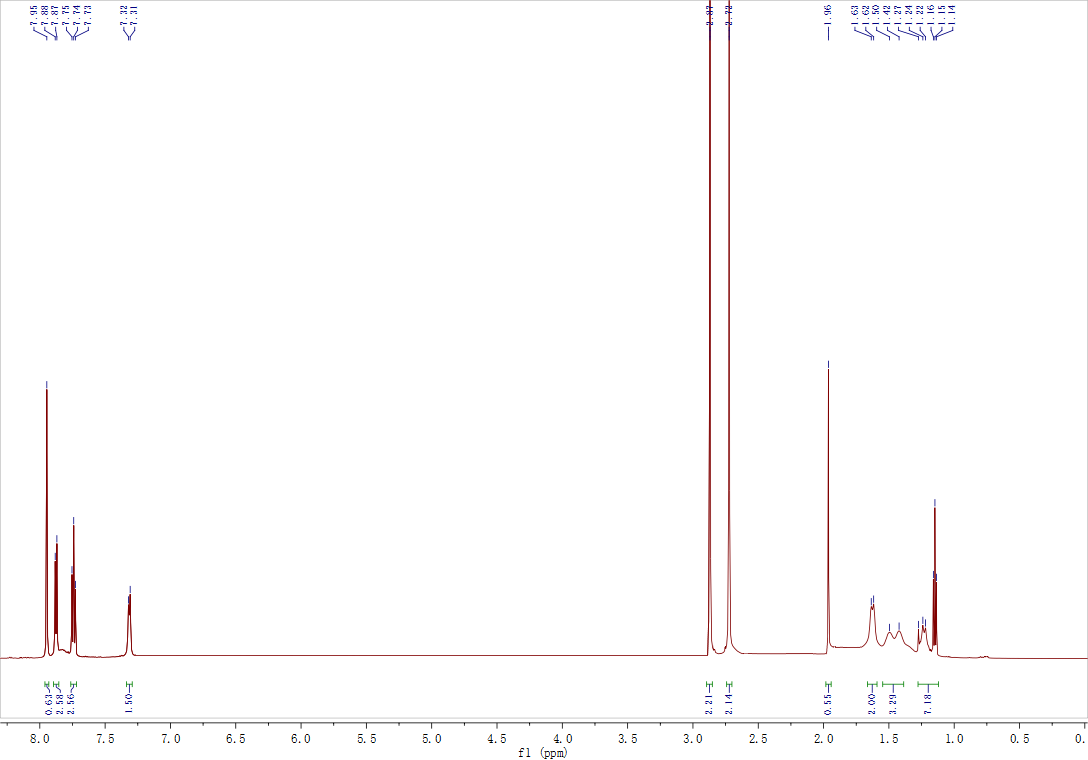
**


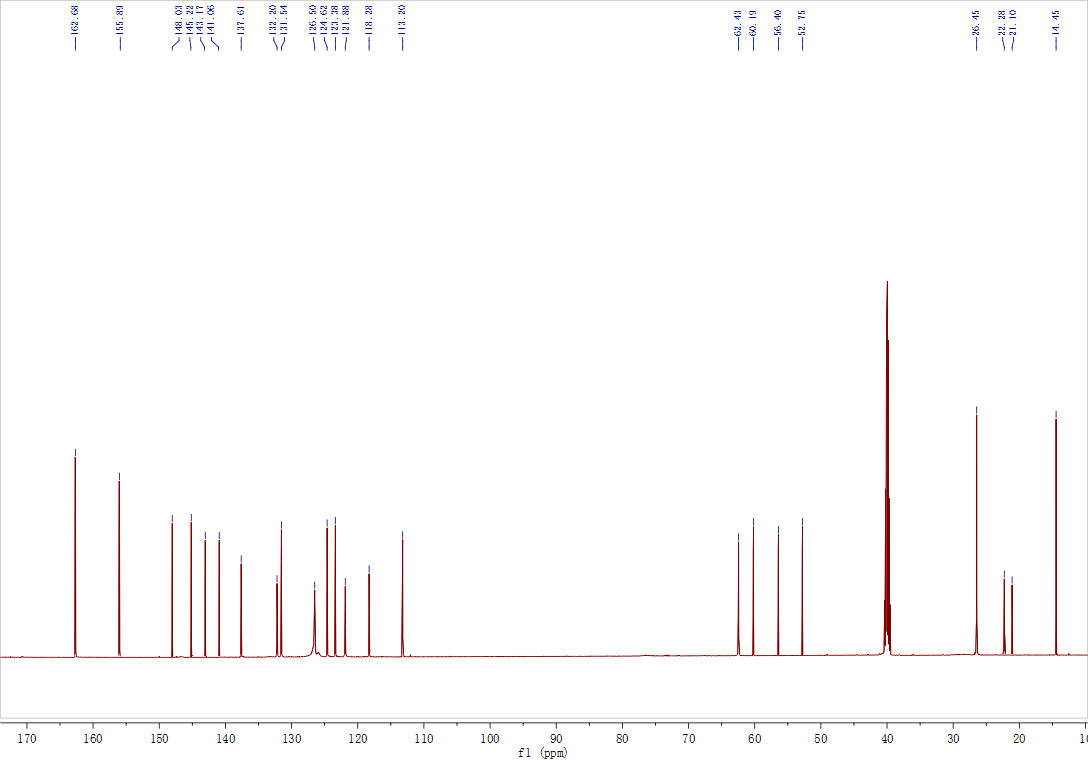


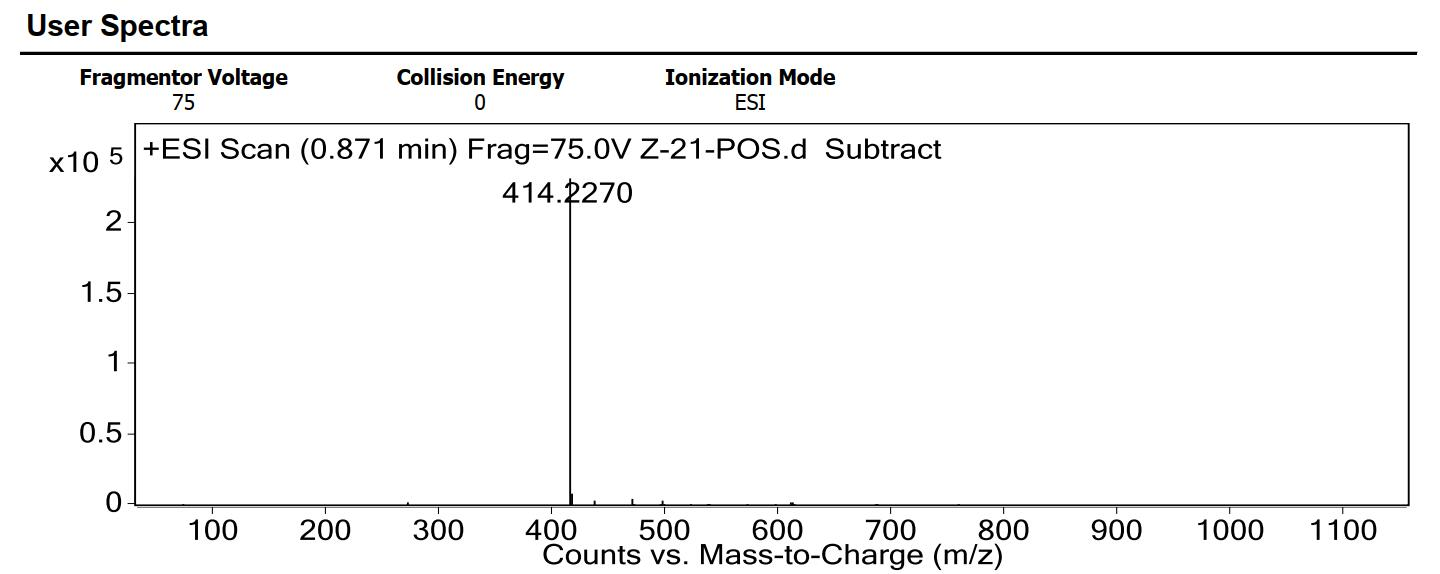


**11v**

**
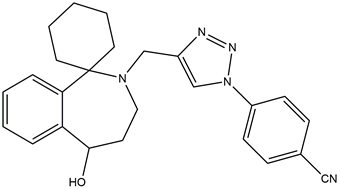
**

**
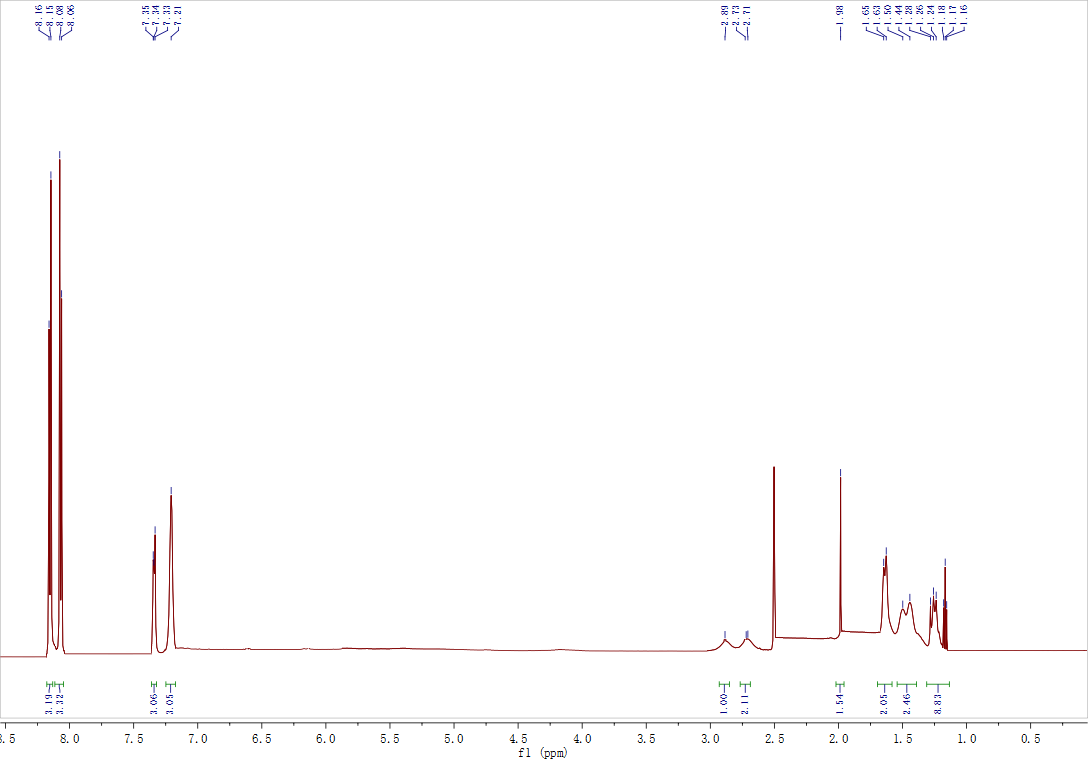
**

**
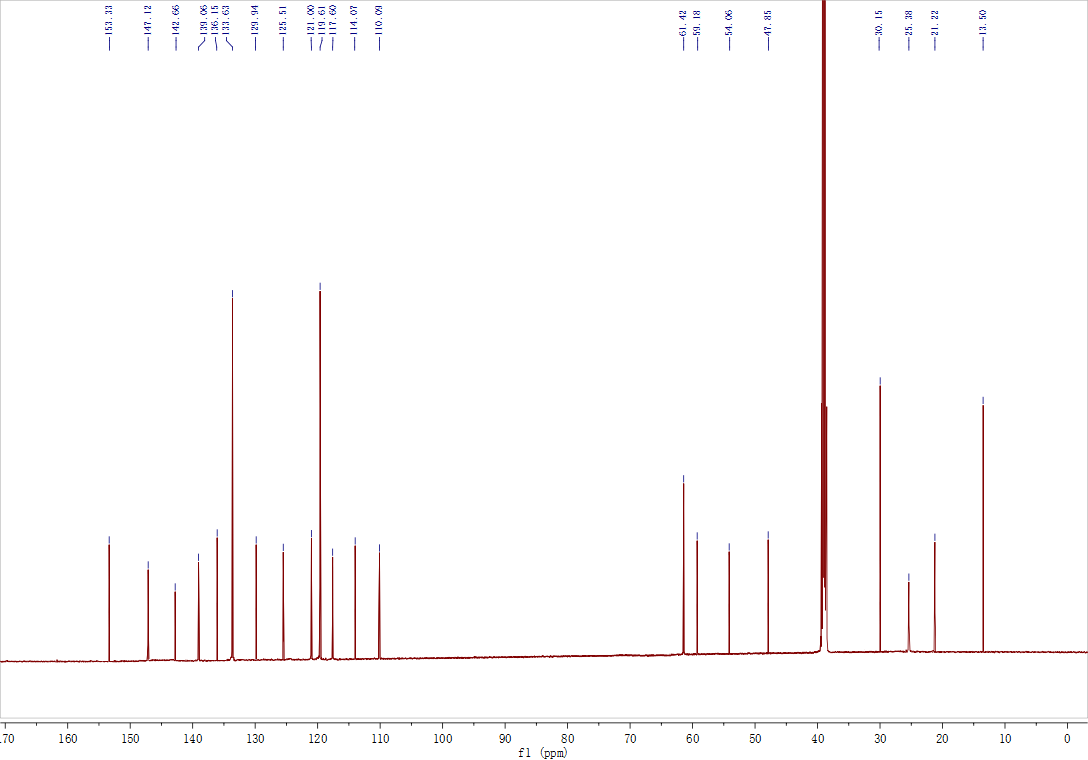
**

**
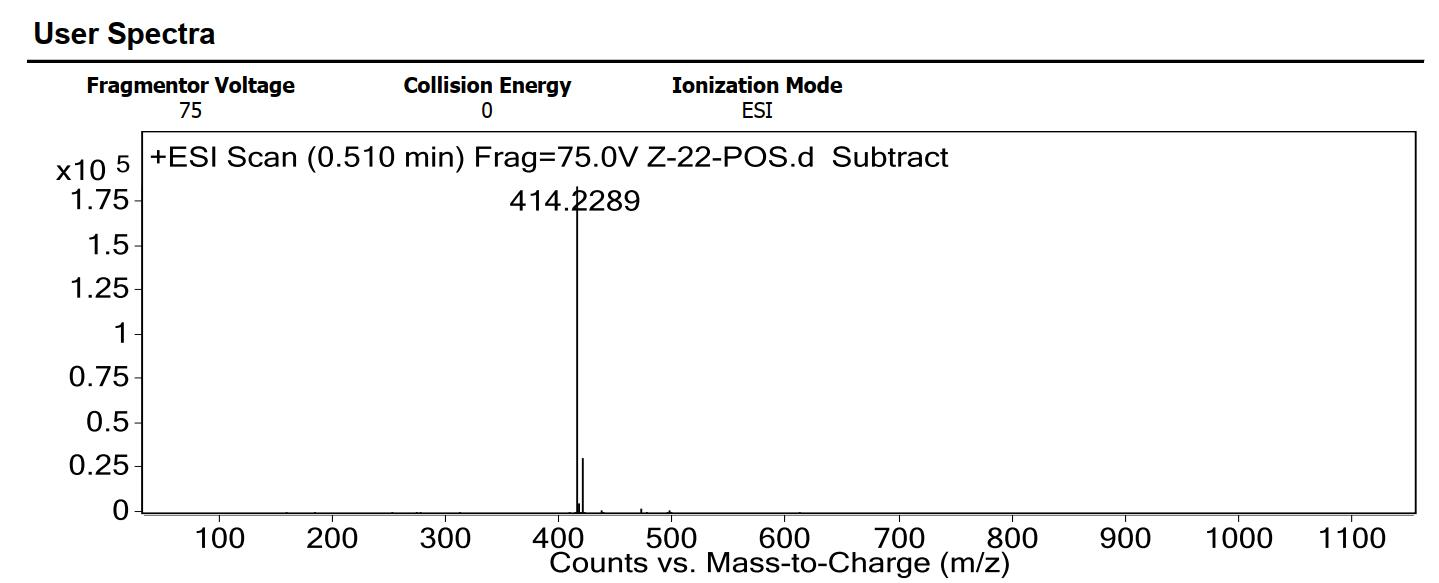
**
